# Effect of Extending the Duration of Prequit Treatment With Varenicline on Smoking Abstinence: A Randomized Clinical Trial

**DOI:** 10.1001/jamanetworkopen.2022.41731

**Published:** 2022-11-11

**Authors:** Larry W. Hawk, Stephen T. Tiffany, Craig R. Colder, Rebecca L. Ashare, Jennifer M. Wray, Rachel F. Tyndale, Thomas H. Brandon, Martin C. Mahoney

**Affiliations:** 1 Department of Psychology, University at Buffalo, Buffalo, New York; 2 Department of Psychiatry, Perelman School of Medicine, University of Pennsylvania, Philadelphia; 3 Department of Psychiatry, Military Sciences Division, Medical University of South Carolina, Charleston; 4 Department of Pharmacology and Toxicology, University of Toronto, Toronto, Ontario, Canada; 5 Department of Psychiatry, University of Toronto, Toronto, Ontario, Canada; 6 Campbell Family Mental Health Research Institute, The Center for Addiction and Mental Health, Toronto, Ontario, Canada; 7 Department of Health Outcomes and Behavior, Moffitt Cancer Center, Tampa, Florida; 8 Department of Psychology, University of South Florida, Tampa; 9 Department of Oncologic Sciences, University of South Florida, Tampa; 10 Department of Internal Medicine, Roswell Park Comprehensive Cancer Center, Buffalo, New York; 11 Department of Health Behavior, Roswell Park Comprehensive Cancer Center, Buffalo, New York

## Abstract

**Importance:**

Even with varenicline, the leading monotherapy for tobacco dependence, smoking abstinence rates remain low. Preliminary evidence suggests that extending the duration of varenicline treatment before quitting may increase abstinence.

**Objective:**

To test the hypotheses that, compared with standard run-in varenicline treatment (1 week before quitting), extended run-in varenicline treatment (4 weeks before quitting) reduces smoking exposure before the target quit date (TQD) and enhances abstinence, particularly among women.

**Design, Setting, and Participants:**

This double-blind, randomized, placebo-controlled clinical trial enrolled participants from October 2, 2017, to December 9, 2020, at a single-site research clinic in Buffalo, New York. Of 1385 people screened, 320 adults reporting smoking 5 or more cigarettes per day (CPD) were randomized and followed up for 28 weeks. Data were analyzed from August 2021 to June 2022.

**Interventions:**

In the pre-TQD period (weeks 1-4), the extended run-in group received 4 weeks of varenicline; the standard run-in group received 3 weeks of placebo followed by 1 week of varenicline. Both groups received open-label varenicline during weeks 5 to 15 and brief quit counseling at 6 clinic visits.

**Main Outcomes and Measures:**

The primary outcome consisted of cotinine-verified (at end of treatment [EOT]) self-reported continuous abstinence from smoking (in CPD) during the last 4 weeks of treatment. Secondary outcomes included bioverified self-report of continuous abstinence at the 6-month follow-up and percentage of reduction in self-reported smoking rate during the prequit period (week 1 vs week 4).

**Results:**

A total of 320 participants were randomized, including 179 women (55.9%) and 141 men (44.1%), with a mean (SD) age of 53.7 (10.1) years. Continuous abstinence during the final 4 weeks of treatment (weeks 12-15; EOT) was not greater in the extended run-in group (64 of 163 [39.3%]) compared with the standard run-in group (57 of 157 [36.3%]; odds ratio [OR], 1.13 [95% CI, 0.72-1.78]), nor was the hypothesized group × sex interaction significant (OR, 0.52 [95% CI, 0.21-1.28]). Similar nonsignificant results were obtained for continuous abstinence at the 6-month follow-up. The mean (SE) decrease in self-reported smoking rate during the prequit period was greater in the extended run-in group (−38.8% [2.8%]) compared with the standard run-in group (−17.5% [2.7%]).

**Conclusions and Relevance:**

Among adult daily smokers, extending the duration of prequit varenicline treatment beyond the standard 1-week run-in period reduced prequit smoking exposure but, more importantly, did not significantly improve continuous abstinence rates.

**Trial Registration:**

ClinicalTrials.gov Identifier: NCT03262662

## Key Points

**Question:**

Does extending the duration of prequit treatment with varenicline improve rates of smoking abstinence?

**Findings:**

In this randomized clinical trial that included 320 adults, the percentage of bioverified continuous 4-week abstinence among participants at the end of treatment was not significantly greater among participants receiving extended (39%) compared with standard (36%) run-in varenicline.

**Meaning:**

These findings show that among adult daily smokers, extending the duration of prequit varenicline treatment beyond the standard 1-week run-in period did not significantly improve continuous abstinence rates.

## Teaser

This randomized clinical trial tests the hypothesis that extended run-in varenicline treatment may produce better smoking abstinence rates than standard varenicline therapy among adults in a single research site.

## Introduction

Since 2006, varenicline has offered the most effective monotherapy for smoking cessation.^1,2,3^ However, long-term abstinence rates with varenicline remain low (20%-25% at 6 months or more).

One approach to improving outcomes is to target a treatment’s putative mechanisms of action.^4,5,6,7^ As a dual agonist and antagonist of nicotinic receptors, varenicline should decrease smoking even before a quit attempt; in learning theory terms, it should promote extinction of smoking behavior as a consequence of decreased reinforcement. Consistent with the hypothesis that varenicline reduces smoking reinforcement, varenicline has reduced nicotine self-administration in rats in a dose-dependent manner.^8,9^ However, extinction learning requires numerous trials and does not generalize well across contexts.^10,11,12,13,14^ Thus, the standard 1-week run-in period for varenicline (during which the dose is titrated) is likely insufficient to maximize extinction. Indeed, the results of placebo-controlled studies of people who are not actively trying to quit smoking suggest that it takes 2 to 3 weeks of varenicline administration to reduce smoking rate and biochemical exposure.^15,16,17^

These findings suggest that the efficacy of varenicline could be enhanced by preloading,^18,19^ or increasing the duration of medication provided before the target quit date (TQD). The results of 2 preliminary randomized clinical trials^20,21^ (RCTs) support this hypothesis. In both trials, extended run-in groups exhibited greater reductions in pre-TQD smoking (without accompanying increases in craving or withdrawal) and nominally higher rates of smoking abstinence at the end of treatment (EOT) relative to standard run-in conditions. Although the results were encouraging, both studies had critical limitations: they were small (100 and 60 participants), used lenient^21^ or no^20^ bioverification, and had only 3 months of follow-up. Results of a subsequent larger trial^22^ (242 participants) suggesting an advantage of extended run-in varenicline treatment are difficult to interpret because participants were instructed to reduce pre-TQD smoking by more than 50%, resulting in considerable pre-TQD attrition, particularly in the standard run-in condition.

The primary aim of the present RCT was to rigorously test the hypothesis that extended run-in varenicline treatment produces better smoking abstinence rates than standard varenicline therapy. The study used a substantially larger sample (N = 320) to provide adequate statistical power, used cotinine levels (which reflect past-week exposure to nicotine) to provide stringent bioverification of self-reported continuous abstinence, and followed up participants for 6 months after the TQD.

The study was also powered to evaluate candidate moderators, specifically sex. Although women generally have lower rates of long-term abstinence than men,^23,24^ women exhibit a greater response to varenicline (relative to placebo) than men.^25^ In a pilot study,^21^ only women showed greater pre-TQD reductions in smoking exposure and greater postquit abstinence rates with extended run-in varenicline treatment (relative to standard varenicline treatment). Replication of those preliminary findings could be of considerable practical importance for helping women quit smoking.

## Methods

The trial protocol and statistical analysis plan for this RCT are provided in Supplement 1. The study was conducted in accordance with the ethical principles of the Declaration of Helsinki^26^ and was approved by the institutional review boards of the University at Buffalo, Buffalo, New York (where data were collected) and the University of Toronto, Toronto, Ontario (where biological assays were performed). All participants provided written informed consent. The study followed the Consolidated Standards of Reporting Trials (CONSORT) reporting guideline.

### Study Design

This RCT used a 2-group, balanced, randomized, double-blind, placebo-controlled, parallel-group design (a trial design schematic is provided in the trial protocol, section 5, in Supplement 1). Groups were distinguished by duration of varenicline treatment before the TQD (ie, run-in duration). The group with extended run-in received 4 weeks of varenicline before TQD. The group with standard run-in received 3 weeks of placebo, followed by the standard 1-week pre-TQD varenicline run-in treatment. Both groups received 11 weeks of post-TQD varenicline treatment and brief quit counseling.

### Randomization

Participants were randomized by the statistician (C.R.C.) in small blocks (2:2) via prespecified tables implemented in REDCap.^27,28^ Randomization was stratified within self-reported sex (an a priori hypothesized moderator, men vs women) and race and ethnicity (non-Hispanic White vs all other racial and ethnic groups); both variables were collected per National Institutes of Health policy.^29^

### Recruitment and Participants

**Figure 1.**
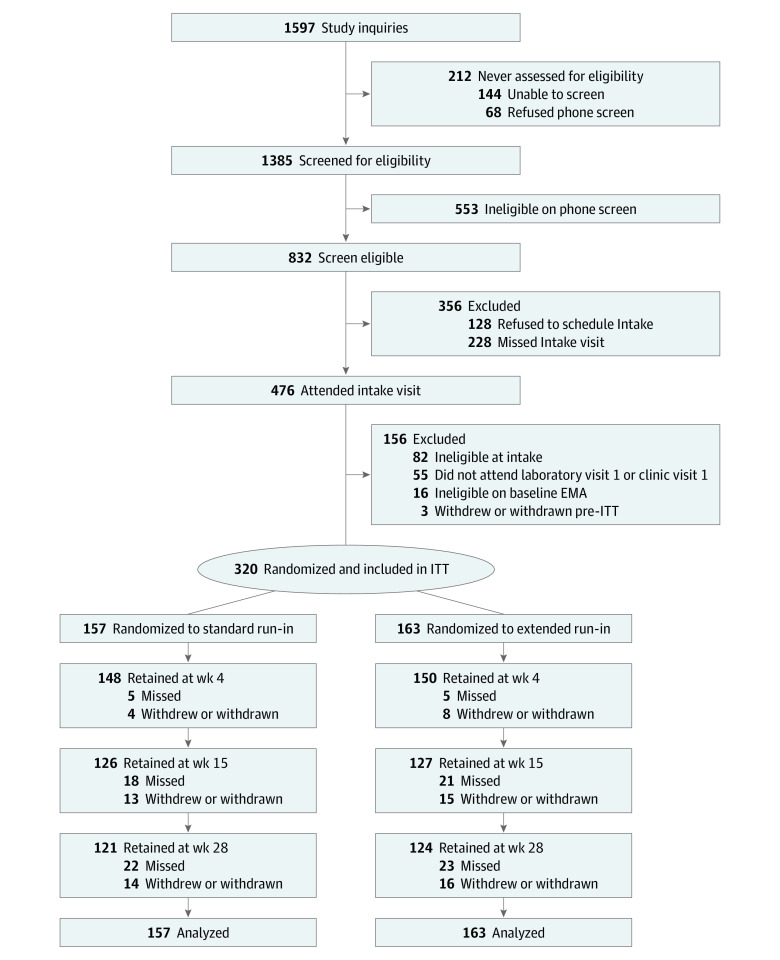
Flow Diagram of Trial Recruitment and Eligibility Evaluation, Intervention Randomization, Treatment and Follow-up, and Analysis EMA indicates ecological momentary assessment; ITT, intention to treat.

Adult smokers of combustible cigarettes who reported living within 50 miles from the University at Buffalo were recruited (beginning September 2017) through advertisements (radio, social media), recruitment databases (state Quitline, ResearchMatch.org), and a project website until the target sample size of 320 participants was enrolled. Inclusion criteria included smoking 10 or more cigarettes per day (CPD) for at least 6 months and an expired-air carbon monoxide level greater than 7 ppm at intake (to reduce exclusion of Black participants, the CPD criterion was reduced to ≥5 and the carbon monoxide criterion was eliminated in November 2019), motivation to quit smoking,^30^ and 18 to 70 years of age. Exclusion criteria included use of other tobacco and/or nicotine products in the past 7 days or use of cessation medication in the past 14 days, lifetime diagnosis of schizophrenia or bipolar disorder, current use of antipsychotic medication, suicidal ideation in the past year,^31^ current major depression,^32^ moderate-to-severe risk of involvement with illicit or nonmedical prescription drug use, and pregnancy. For additional details, see the trial protocol, section 6, in Supplement 1. Participant disposition is summarized in Figure 1, and demographic and smoking characteristics are presented in the Table. Participants were enrolled from October 2, 2017, to December 9, 2020.

**Table.** Baseline Participant Characteristics for Each Run-in Group and Each Sex × Group Condition

Characteristic	Participant group^a^					
	Men		Women		All	
	Standard run-in (n = 68)	Extended run-in (n = 73)	Standard run-in (n = 89)	Extended run-in (n = 90)	Standard run-in (n = 157)	Extended run-in (n = 163)
Age, mean (SD), y	52.7 (10.9)	53.2 (10.9)	54.2 (9.3)	54.2 (9.8)	53.5 (10.0)	53.8 (10.3)
Race						
Black or African American	10 (14.7)	9 (12.3)	24 (27.0)	24 (26.7)	34 (21.7)	33 (20.2)
White	55 (80.9)	59 (80.8)	63 (70.8)	63 (70.0)	118 (75.2)	122 (74.8)
Other^b^	3 (4.4)	5 (6.8)	2 (2.2)	3 (3.3)	5 (3.2)	8 (4.9)
Hispanic ethnicity	2 (2.9)	1 (1.4)	2 (2.2)	3 (3.3)	4 (2.5)	4 (2.5)
Educational level past high school^c^	47 (72.3)	47 (67.1)	67 (77.0)	68 (77.3)	114 (75.0)	115 (72.8)
Employed full-time^d^	34 (52.3)	33 (48.5)	37 (42.5)	38 (43.2)	71 (46.7)	71 (45.5)
Income ≥$50 000^e^	39 (63.9)	40 (60.6)	43 (51.8)	40 (49.4)	82 (56.9)	80 (54.4)
Married or living with partner^f^	37 (57.8)	41 (57.8)	47 (54.0)	45 (51.7)	84 (55.6)	86 (54.4)
BMI, mean (SD)	30.9 (5.7)	30.3 (6.3)	31.1 (8.0)	29.7 (6.7)	31.0 (7.1)	30.0 (6.5)
Body weight, mean (SD), kg	96.2 (20.1)	93.8 (22.5)	83.5 (23.2)	79.0 (18.4)	89.0 (22.7)	85.7 (21.6)
Age first smoked daily, mean (SD), y	18.7 (3.6)	17.9 (3.6)	18.5 (3.5)	18.8 (3.5)	18.6 (3.6)	18.4 (3.6)
Duration of daily smoking, mean (SD), y	33.7 (11.4)	35.1 (11.2)	35.8 (9.5)	35.3 (9.7)	34.9 (10.4)	35.2 (10.3)
CPD in past 6 mo, mean (SD)	20.3 (8.1)	19.5 (8.0)	17.0 (6.2)	16.5 (6.1)	18.4 (7.3)	17.9 (7.2)
FTCD score, mean (SD)^g^	5.5 (2.2)	5.5 (1.8)	5.6 (1.9)	5.4 (2.1)	5.6 (2.0)	5.4 (2.0)
Smokes menthol cigarettes^h^	25 (39.1)	25 (35.2)	50 (57.5)	55 (62.5)	75 (49.7)	80 (50.3)
Lives with another smoker^i^	16 (32.7)	14 (29.8)	23 (33.8)	30 (47.6)	39 (33.3)	44 (40.0)
Expired-air CO level, mean (SD), ppm	27.7 (14.0)	28.4 (12.7)	27.1 (15.6)	28.1 (13.2)	27.4 (14.9)	28.2 (13.0)
Salivary cotinine level, mean (SD), ng/mL	308.8 (169)	319.8 (172)	314.0 (160)	305.6 (146)	311.8 (164)	312.0 (158)
NMR, mean (SD)	0.42 (0.32)	0.47 (0.34)	0.52 (0.50)	0.62 (0.42)	0.48 (0.43)	0.55 (0.39)
No. of prior quit attempts, mean (SD)	4.2 (3.2)	4.6 (3.3)	4.6 (3.2)	4.1 (3.0)	4.4 (3.2)	4.4 (3.1)
Longest quit duration >8 wk^j^	37 (56.9)	35 (49.3)	51 (58.6)	53 (60.2)	88 (57.9)	88 (55.3)

Abbreviations: BMI, body mass index (calculated as weight in kilograms divided by height in meters squared); CO, carbon monoxide; CPD, cigarettes per day; FTCD, Fagerstrom Test of Cigarette Dependence; NMR, nicotine metabolite ratio.

^a^ Unless otherwise indicated, data are expressed as No. (%) of participants. To convert cotinine level to nmol/L, multiply by 5.675.

^b^ Includes American Indian or Alaska Native, more than 1 race or ethnicity, and other race or ethnicity.

^c^ Data were missing for 10 of 320 participants (3.1%).

^d^ Data were missing for 12 of 320 participants (3.8%).

^e^ Data were missing for 29 of 320 participants (9.1%).

^f^ Data were missing for 11 of 320 participants (3.4%).

^g^ Scores range from 0 to 10, with higher scores indicating greater dependence on cigarettes.

^h^ Data were missing for 10 of 320 participants (3.1%).

^i^ Data were missing for 93 of 320 participants (29.1%). This variable was added partway through the study.

^j^ Data were missing for 9 of 320 participants (2.8%).

### Study Procedures

After an initial telephone screen, participants completed an intake visit (at which participants provided written informed consent), a baseline laboratory visit (discontinued March 2020 owing to the SARS-CoV-2 pandemic; data are reported elsewhere^33^), and a baseline week of daily smartphone assessments. Participants made 6 clinic visits to the University of Buffalo–based research clinic at baseline (week 0), during the pre-TQD drug manipulation phase (weeks 1 and 3), on the TQD (week 4), and at weeks 6 and 8. Follow-up visits occurred at week 15 (EOT) and week 28 (6 months). All visits included assessment of vital signs; measurement of expired-air carbon monoxide level (Bedfont Inc); self-reports of smoking rate, craving, affect, withdrawal, and adverse events; and collection of a saliva sample for cotinine, trans-3′-hydroxycotinine (3HC), and varenicline assessment. Study medication and counseling (described below) were provided at each clinic visit. Participants were compensated as much as US $598 for completing study visits and measures.

### Interventions

#### Pharmacotherapy

Pfizer Inc provided varenicline and identically appearing placebo, which was dispensed at each clinic visit (except during the COVID-19 shutdown [April 1 to June 15, 2020], when dispensing occurred less frequently). Participants were initially dispensed a 1-week supply of tablets labeled 0.5 mg to be taken orally (1 morning tablet for days 1-3 and 1 morning and 1 evening tablet for days 4-7 consisting of varenicline for the extended run-in group and placebo for the standard run-in group). During weeks 2 and 3 of the medication manipulation phase, participants were given 1.0-mg tablets to be taken twice daily (varenicline for the extended run-in group and placebo for the standard run-in group). To maintain blinding and allow titration for participants assigned to the standard run-in group, during week 4 all participants were provided with 4 tablets/d (2 for morning and 2 for evening) marked 0.5 mg (varenicline for the extended run-in group; for the standard run-in group, 1 of 2 morning tablets was varenicline and, for days 4-7, 1 of 2 evening tablets was varenicline). Beginning with the TQD, all participants were dispensed open-label 1.0-mg varenicline twice daily.

#### Counseling

Participants received brief (approximately 15 minutes) behavioral counseling per a treatment manual adapted from prior studies.^21,34^ Counseling focused on honing quit motivation; preventing or managing adverse effects, stress, and smoking triggers; and relapse prevention. To promote continued smoking and foster extinction, participants were instructed to smoke as usual during weeks 1 to 3.^21,35,36^ Brief (approximately 5-minute) telephone check-ins occurred during weeks 5 and 11.

### Outcome Measures

Our prespecified primary outcome was continuous abstinence at EOT (no self-reported cigarette smoking [not even a puff] during the final 4 weeks of treatment [weeks 12-15]), bioverified with EOT salivary cotinine level of 15 ng/mL or less (to convert to nmol/L, multiply by 5.675). Continuous abstinence at the 6-month follow-up (no self-reported smoking during weeks 12-28, cotinine level ≤15 ng/mL at EOT and 6 months) and percentage reduction in smoking behavior (CPD) during the prequit phase of the study were prespecified secondary outcomes. Self-reported smoking was assessed via timeline follow-back interview.^37^ Salivary cotinine and 3HC (used for the ratio of 3HC to cotinine, or the nicotine metabolite ratio) were assayed using liquid chromatography–mass spectrometry (lower limit of quantification, 1 ng/mL).^38,39,40^ Unregistered outcomes included cotinine concentrations during the prequit period (weeks 0, 1, 3, and 4); bioverified 7-day point-prevalence abstinence at weeks 6, 8, 15 (EOT), and 28 (6 months); and the following measures at all clinic visits: self-report measures of current craving^41^ and past-week nicotine withdrawal,^42^ pill count adherence, and adverse events (assessed via a 32-item symptom checklist^21,36,43^ and open-ended queries).

### Sample Size Calculation

A priori analysis used Monte Carlo simulations and abstinence rates from the pilot study^21^ and focused on the primary outcome of continuous abstinence at EOT with 2-tailed α = .05 and power of 0.90. With 320 participants, the study was powered to detect an increase in abstinence rate of at least 13% in the extended run-in group, compared with an expected 40% abstinence rate in the standard run-in group. With respect to sex moderation, the study was powered to detect a difference of increased abstinence of 32% in the extended run-in group for women compared with a decline in abstinence rate of 10% in extended run-in group for men, as previously observed.^21^

### Statistical Analysis

Data were analyzed from August 2021 to June 2022. Analyses (conducted in SPSS, version 28 [IBM Corp] and Mplus, version 8.8 [Muthén & Muthén]) included run-in group, sex, and their interaction. Significance level was 2-tailed α = .05. The primary outcome, continuous smoking abstinence during the final 4 weeks of treatment (bioverified at EOT), was analyzed based on intention-to-treat (N = 320) via logistic regression; participants with missing data were considered smokers (results of supplemental multiple imputation analyses are described below). Continuous abstinence at 6 months (a secondary outcome) was analyzed similarly. Percentage reduction in self-reported smoking rate (CPD) during the prequit period (a secondary outcome) was analyzed in a group × sex analysis of variance.

## Results

### Baseline Characteristics

A total of 320 participants were randomized, including 179 women (55.9%) and 141 men (44.1%), with a mean (SD) age of 53.7 (10.1) years. The Table provides demographic and smoking characteristics for all treatment groups and groups by sex (eAppendix 1, eTable A1, in Supplement 2). Participants reported smoking a mean (SD) of 18.1 (7.2) CPD and were moderately nicotine dependent (mean [SD] Fagerstrom Test of Cigarette Dependence score, 5.5 [2.0]). Women had a faster nicotine metabolite ratio (mean [SD], 0.57 [0.46] vs 0.44 [0.33]), lower body weight (mean [SD], 81.2 [21.0] vs 95.0 [21.3] kg), fewer CPD (mean [SD], 16.8 [6.2] vs 19.9 [8.1]), and more years of education (educational attainment beyond high school, 135 [75.4%] vs 94 [66.7%]) than men.

### Participants

As shown in Figure 1, retention was comparable between the extended and standard run-in groups at EOT (127 of 163 [77.9%] and 126 of 157 [80.3%]) and at 6 months (124 of 163 [76.1%] and 121 of 157 [77.1%]; *P* > .71). Retention at 6 months was somewhat lower among men in the extended run-in group compared with men in the standard run-in group (48 of 73 [65.8%] vs 53 of 68 [77.9%]) and compared with women in the extended run-in group (76 of 90 [84.4%]) and women in the standard run-in group (68 of 89 [76.4%]) (group × sex *P* = .17 at EOT and *P* = .04 at 6 months).

### Primary Outcome

**Figure 2.**
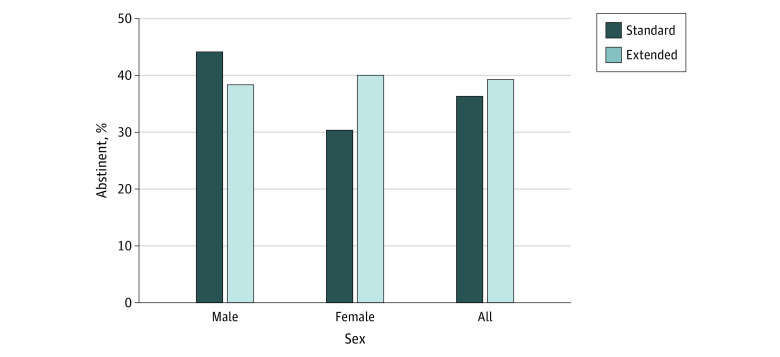
Bioverified Continuous Abstinence Rates for Each Run-in Group and Each Group × Sex Condition at End of Treatment Bioverification used continine levels to verify rates at end of treatment (primary outcome).

Contrary to our hypothesis, continuous abstinence at EOT (Figure 2) was not significantly greater in the extended compared with the standard run-in group (64 of 163 [39.3%] vs 57 of 157 [36.3%]; odds ratio [OR], 1.13 [95% CI, 0.72-1.78]; *P* = .60) and did not significantly differ between women (63 of 179 [35.2%]) and men (58 of 141 [41.1%]) (OR, 1.29 [95% CI, 0.82-2.02]; *P* = .28). Most importantly, the hypothesized group × sex interaction was not significant overall (OR, 0.52 [95% CI, 0.21-1.28]; *P* = .15), nor was the post-hoc test of the run-in group effect among women (OR, 1.53 [95% CI, 0.83-2.84]; *P* = .18) or men (OR, 0.79 [95% CI, 0.40-1.54]; *P* = .49).

### Secondary Outcomes

####  Abstinence at 6 Months

**Figure 3.**
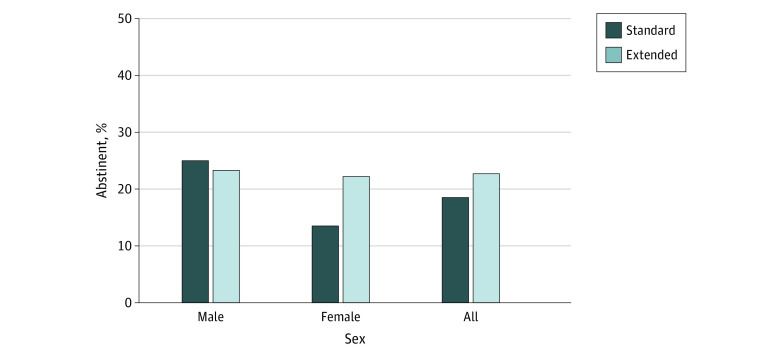
Bioverified Continuous Abstinence Rates for Each Run-in Group and Each Sex × Group Condition at 6-Month Follow-up Bioverification used continine levels to verify rates at 6-month follow-up (secondary outcome).

The results for continuous abstinence at 6 months were similar to those obtained at EOT (Figure 3). We observed no significant effects in run-in group (OR, 1.29 [95% CI, 0.75-2.23]; *P* = .36), sex (OR, 1.46 [95% CI, 0.85-2.51]; *P* = .18), or group × sex interaction (OR, 0.50 [95% CI, 0.17-1.49]; *P* = .21). Similarly, post-hoc tests of the run-in group effect among women (OR, 1.83 [95% CI, 0.84-4.02]; *P* = .13) and men (OR, 0.91 [95% CI, 0.42-1.97]; *P* = .81) were not significant.

Supplemental analyses of continuous abstinence at EOT and 6 months that incorporated covariates, considered moderators, and used multiple imputation for missingness all yielded similar results; there were no statistically significant main effects or interactions involving the run-in group (eAppendix 2 in Supplement 2). The same was true in analyses of 7-day point prevalence abstinence at EOT and 6 months (eAppendix 2 in Supplement 2).

#### Percentage Reduction in Pre-TQD Smoking Rate

**Figure 4.**
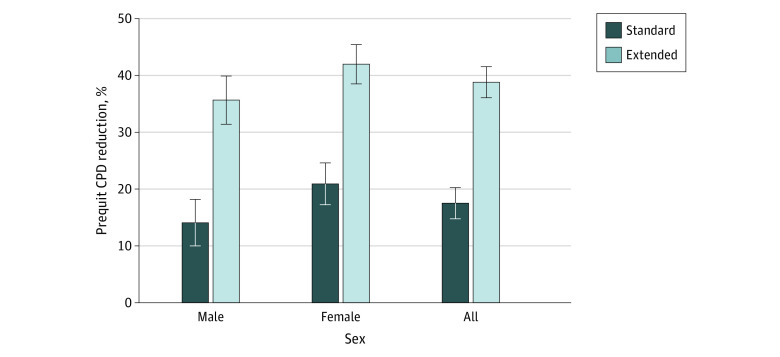
Percentage Reduction in Self-reported Smoking Rate for Each Run-in Group and Each Sex × Group Condition Reduction is measured as self-reported cigarettes smoked per day (secondary outcome). Error bars indicate 1 SE. CPD indicates cigarettes per day.

As hypothesized, although both groups self-reported reducing their smoking rate (in CPD) during the prequit period (Figure 4), the reduction was greater in the extended run-in group compared with the standard run-in group (mean [SE], −38.8% [2.8%] vs −17.5% [2.7%]; *P* < .001). Although women tended to report greater reduction in pre-TQD CPD compared with men (mean [SE], −31.4% [2.5%] vs −24.9% [2.9%]; *P* = .09), there was no evidence that the group difference varied between men (means [SE], −35.6% [4.2%] for extended and −14.1% [4.1%] for standard groups) and women (mean [SE], −42.0% [3.5%] for extended and −20.9% [3.7%] for standard groups; group × sex interaction; *P* = .95). Supplemental analyses of week-to-week changes in prequit CPD and cotinine levels yielded comparable results (eAppendix 3 in Supplement 2).

### Unregistered Outcomes

#### Craving and Withdrawal

Consistent with our hypothesis, craving at week 4 (TQD) was greater in the standard compared with the extended run-in group, a difference that was stronger among women than men. From weeks 4 to 8, withdrawal increased in the standard run-in group but declined in the extended run-in group, a pattern that tended to be stronger among women than men (details in eAppendix 4 in Supplement 2).

#### Adherence

Pill count adherence (mean [SD], 82.5% [29.2%]; 244 of 318 [76.7%] of the sample were ≥80% adherent) was assessed during the 15-week treatment period. Results did not vary as a function of group, sex, or their interaction (eAppendix 5 in Supplement 2).

#### Adverse Events

Symptom reports were generally as expected for varenicline (details are provided in eAppendix 6 in Supplement 2). During the run-in manipulation period (weeks 1-3), nausea and abnormal dreams were more common among participants in the extended run-in group vs those in the standard run-in group; these differences were not significant once the standard run-in group began active varenicline therapy. Similar patterns were observed for multiple gastrointestinal tract symptoms, other sleep problems, and dizziness. Consistent with our hypotheses, the group difference in nausea was primarily driven by women. Serious adverse events were rare (3 in each run-in group) and deemed unexpected and unrelated to study medication (details in eAppendix 6 in Supplement 2).

## Discussion

Previous large RCTs have demonstrated that both standard and extended run-in varenicline treatments enhance smoking abstinence relative to placebo treatment,^2,44,45^ but these trials do not address the question of whether extended run-in varenicline treatment is better than standard varenicline treatment. From the perspective of extinction learning, a longer duration of pre-TQD varenicline treatment would allow numerous prequit opportunities for participants to experience a reduction of reinforcement while smoking, resulting in reduced prequit smoking exposure (without increasing craving or withdrawal) and improved postquit abstinence rates.

As hypothesized in the present RCT, the extended run-in group exhibited reduced smoking exposure during the prequit period (ie, lower self-reported smoking rate and cotinine levels) and attenuated peri-TQD craving and withdrawal relative to the standard run-in group. Rates of attrition were typical, albeit somewhat lower at 6 months for men in the extended run-in group. Overall, from a mechanism and process perspective, the trial results were largely as expected and supported the internal validity of the study.

However, rates of bioverified, self-reported continuous smoking abstinence at the 3-month (EOT; the primary outcome) and 6-month follow-ups were not significantly higher for extended run-in varenicline treatment. Thus, preliminary support for extended prequit varenicline treatment observed in a pair of pilot RCTs funded by Pfizer Inc,^20,21^ was not substantiated in the present, much larger trial (320 participants vs 100 for Hajek et al^20^ and 60 for Hawk et al^21^). Although abstinence rates in the standard run-in condition were somewhat lower than estimated based on the pilot study,^21^ they were similar to rates observed in recent large-scale varenicline trials.^43,46^

Based on the pilot RCT^21^ and other evidence of sex differences in the neurobiology and behavioral pharmacology of smoking and of smoking cessation,^23,24,25,47^ we hypothesized that the extended run-in varenicline treatment would outperform standard run-in treatment, specifically among women. Although the rates of abstinence were in the hypothesized direction, the interaction was not statistically significance at EOT or at 6 months. Moreover, there was no evidence that sex moderated the run-in group effect on pre-TQD changes in smoking behavior (CPD) or exposure (cotinine level). These data argue against the a priori hypothesis that pre-TQD varenicline treatment would extinguish smoking to a greater extent among women than men.^33^

Craving and withdrawal (unregistered but commonly reported outcomes in varenicline trials^1,20,21,48,49^) were moderated by sex in a manner consistent with the hypothesized greater benefit for women. It is possible that extended run-in varenicline treatment potentiates, at least among women, the mechanisms of action recently reported^50^ for standard varenicline (reduced craving and negative affect). However, in the present study, this did not translate into a significant enhancement of smoking abstinence. The present study may have been underpowered to detect the effect on dichotomous abstinence, because our a priori power analysis was based on a preliminary study^21^ with a small sample size, limiting the precision of the estimate. Given that women generally have more difficulty maintaining long-term abstinence than men,^23^ it may be worthwhile to test these hypotheses in the context of a larger, women-only multisite trial.

### Limitations

In addition to the concern about statistical power just mentioned, additional limitations merit consideration. First, internal validity could have been compromised by the confounding of the duration of prequit varenicline treatment and total duration of varenicline treatment. That is, as in prior studies of medication preloading,^18,20,21,22,35,36,51^ the extended run-in group received a longer total duration of varenicline treatment (15 weeks) than did the standard run-in group (12 weeks). However, because even doubling the duration of postquit varenicline treatment from 11 to 23 weeks did not enhance long-term abstinence in a large, recent trial,^52^ any effects of extended prequit varenicline on abstinence—had they been statistically significant—were unlikely to be accounted for by the total duration of treatment. Second, although we followed recommendations to relax enrollment eligibility criteria in comparison with earlier trials, external validity remained limited by the exclusion of participants with conditions that are comorbid with smoking, including depression, schizophrenia, and problematic use of illicit and prescription drugs.^53^ Third, the a priori focus on a relatively short follow-up period (3 months) for the primary abstinence outcome, although common in varenicline trials^1,45,48,54,55^ and supplemented by a 6-month follow-up, is also a limitation.

## Conclusions

In this RCT, although extended run-in varenicline treatment reduced pre-TQD smoking rate to a greater extent than standard run-in varenicline treatment, it did not significantly enhance rates of smoking abstinence at the 3- or the 6-month follow-up. Thus, on average, adult daily smokers did not significantly benefit from extending the duration of prequit treatment with varenicline beyond the standard 1-week run-in period.

## Notes

Supplement 1.
Trial Protocol and Statistical Analysis Plan

Supplement 2. **eAppendix 1.** Supplemental Information on Categorical Participant Characteristics **eAppendix 2.** Supplemental Abstinence Analyses **eAppendix 3.** Supplemental Analyses of Reduction in Smoking Exposure Across the Prequit Period **eAppendix 4.** Craving and Withdrawal **eAppendix 5.** Pill Count Medication Adherence **eAppendix 6.** Symptom Report Data

Supplement 3.
Data Sharing Statement

## References

[1] Gonzales D, Rennard SI, Nides M, ; Varenicline Phase 3 Study Group. Varenicline, an alpha4beta2 nicotinic acetylcholine receptor partial agonist, vs sustained-release bupropion and placebo for smoking cessation: a randomized controlled trial. JAMA. 2006;296(1):47-55. doi:10.1001/jama.296.1.47 16820546

[2] Cahill K, Stevens S, Perera R, Lancaster T. Pharmacological interventions for smoking cessation: an overview and network meta-analysis. Cochrane Database Syst Rev. 2013;(5):CD009329. doi:10.1002/14651858.CD009329.pub2 23728690 PMC8406789

[3] Baker TB, McCarthy DE. Smoking treatment: a report card on progress and challenges. Annu Rev Clin Psychol. 2021;17:1-30. doi:10.1146/annurev-clinpsy-081219-090343 33962535

[4] Kraemer HC, Wilson GT, Fairburn CG, Agras WS. Mediators and moderators of treatment effects in randomized clinical trials. Arch Gen Psychiatry. 2002;59(10):877-883. doi:10.1001/archpsyc.59.10.877 12365874

[5] MacKinnon DP. Introduction to Statistical Mediation Analysis. Routledge; 2008.

[6] Rigotti NA. Improving the success of treating tobacco smokers. JAMA Intern Med. 2015;175(2):272-273. doi:10.1001/jamainternmed.2014.6921 25545347

[7] Gritz ER, ed. *Tobacco Research Implementation Plan: Priorities for Tobacco Research Beyond the Year 2000*. National Cancer Institute, National Institutes of Health; 1998.

[8] O’Connor EC, Parker D, Rollema H, Mead AN. The alpha4beta2 nicotinic acetylcholine-receptor partial agonist varenicline inhibits both nicotine self-administration following repeated dosing and reinstatement of nicotine seeking in rats. Psychopharmacology (Berl). 2010;208(3):365-376. doi:10.1007/s00213-009-1739-5 19967529

[9] Rollema H, Chambers LK, Coe JW, . Pharmacological profile of the alpha4beta2 nicotinic acetylcholine receptor partial agonist varenicline, an effective smoking cessation aid. Neuropharmacology. 2007;52(3):985-994. doi:10.1016/j.neuropharm.2006.10.016 17157884

[10] Bouton ME. Context and behavioral processes in extinction. Learn Mem. 2004;11(5):485-494. doi:10.1101/lm.78804 15466298

[11] Bouton ME. Context, ambiguity, and unlearning: sources of relapse after behavioral extinction. Biol Psychiatry. 2002;52(10):976-986. doi:10.1016/S0006-3223(02)01546-9 12437938

[12] Bouton ME, Winterbauer NE, Todd TP. Relapse processes after the extinction of instrumental learning: renewal, resurgence, and reacquisition. Behav Processes. 2012;90(1):130-141. doi:10.1016/j.beproc.2012.03.004 22450305 PMC3355659

[13] Collins BN, Brandon TH. Effects of extinction context and retrieval cues on alcohol cue reactivity among nonalcoholic drinkers. J Consult Clin Psychol. 2002;70(2):390-397. doi:10.1037/0022-006X.70.2.390 11952197

[14] Conklin CA, Tiffany ST. Applying extinction research and theory to cue-exposure addiction treatments. Addiction. 2002;97(2):155-167. doi:10.1046/j.1360-0443.2002.00014.x 11860387

[15] Ashare RL, Tang KZ, Mesaros AC, Blair IA, Leone F, Strasser AA. Effects of 21 days of varenicline versus placebo on smoking behaviors and urges among non–treatment seeking smokers. J Psychopharmacol. 2012;26(10):1383-1390. doi:10.1177/0269881112449397 22695488 PMC3526838

[16] Poling J, Rounsaville B, Gonsai K, Severino K, Sofuoglu M. The safety and efficacy of varenicline in cocaine using smokers maintained on methadone: a pilot study. Am J Addict. 2010;19(5):401-408. doi:10.1111/j.1521-0391.2010.00066.x 20716302 PMC2966972

[17] Brandon TH, Drobes DJ, Unrod M, . Varenicline effects on craving, cue reactivity, and smoking reward. Psychopharmacology (Berl). 2011;218(2):391-403. doi:10.1007/s00213-011-2327-z 21559801 PMC4667942

[18] Preloading Investigators. Effects on abstinence of nicotine patch treatment before quitting smoking: parallel, two arm, pragmatic randomised trial. BMJ. 2018;361:k2164. 29899061 10.1136/bmj.k2164 PMC5998048

[19] Lindson N, Aveyard P. An updated meta-analysis of nicotine preloading for smoking cessation: investigating mediators of the effect. Psychopharmacology (Berl). 2011;214(3):579-592. doi:10.1007/s00213-010-2069-3 21060996

[20] Hajek P, McRobbie HJ, Myers KE, Stapleton J, Dhanji AR. Use of varenicline for 4 weeks before quitting smoking: decrease in ad lib smoking and increase in smoking cessation rates. Arch Intern Med. 2011;171(8):770-777. doi:10.1001/archinternmed.2011.138 21518946

[21] Hawk LW Jr, Ashare RL, Lohnes SF, . The effects of extended pre-quit varenicline treatment on smoking behavior and short-term abstinence: a randomized clinical trial. Clin Pharmacol Ther. 2012;91(2):172-180. doi:10.1038/clpt.2011.317 22130118 PMC3325094

[22] Bohadana A, Freier-Dror Y, Peles V, Babai P, Izbicki G. Extending varenicline preloading to 6 weeks facilitates smoking cessation: a single-site, randomised controlled trial. EClinicalMedicine. 2020;19:100228. doi:10.1016/j.eclinm.2019.11.021 32055787 PMC7005428

[23] Smith PH, Bessette AJ, Weinberger AH, Sheffer CE, McKee SA. Sex/gender differences in smoking cessation: a review. Prev Med. 2016;92:135-140. doi:10.1016/j.ypmed.2016.07.013 27471021 PMC5085924

[24] Piper ME, Cook JW, Schlam TR, . Gender, race, and education differences in abstinence rates among participants in two randomized smoking cessation trials. Nicotine Tob Res. 2010;12(6):647-657. doi:10.1093/ntr/ntq067 20439385 PMC2878731

[25] McKee SA, Smith PH, Kaufman M, Mazure CM, Weinberger AH. Sex differences in varenicline efficacy for smoking cessation: a meta-analysis. Nicotine Tob Res. 2016;18(5):1002-1011. doi:10.1093/ntr/ntv207 26446070 PMC5942618

[26] World Medical Association. WMA Declaration of Helsinki—ethical principles for medical research involving human subjects. September 6, 2022. Accessed October 13, 2022. https://www.wma.net/policies-post/wma-declaration-of-helsinki-ethical-principles-for-medical-research-involving-human-subjects/

[27] Harris PA, Taylor R, Thielke R, Payne J, Gonzalez N, Conde JG. Research electronic data capture (REDCap)—a metadata-driven methodology and workflow process for providing translational research informatics support. J Biomed Inform. 2009;42(2):377-381. doi:10.1016/j.jbi.2008.08.010 18929686 PMC2700030

[28] Harris PA, Taylor R, Minor BL, ; REDCap Consortium. The REDCap Consortium: building an international community of software platform partners. J Biomed Inform. 2019;95:103208. doi:10.1016/j.jbi.2019.103208 31078660 PMC7254481

[29] National Institutes of Health. NIH policy on reporting race and ethnicity data: subjects in clinical research. August 8, 2001. Accessed September 15, 2022. https://grants.nih.gov/grants/guide/notice-files/not-od-01-053.html

[30] Kotz D, Brown J, West R. Predictive validity of the Motivation to Stop Scale (MTSS): a single-item measure of motivation to stop smoking. Drug Alcohol Depend. 2013;128(1-2):15-19. doi:10.1016/j.drugalcdep.2012.07.012 22943961

[31] Posner K, Brent D, Lucas C, . Columbia-Suicide Severity Rating Scale (C-SSRS). Columbia University Medical Center; 2008.

[32] Kroenke K, Spitzer RL, Williams JB. The PHQ-9: validity of a brief depression severity measure. J Gen Intern Med. 2001;16(9):606-613. doi:10.1046/j.1525-1497.2001.016009606.x 11556941 PMC1495268

[33] Lawson SC, Gass JC, Cooper RK Jr, . The impact of three weeks of pre-quit varenicline on reinforcing value and craving for cigarettes in a laboratory choice procedure. Psychopharmacology (Berl). 2021;238(2):599-609. doi:10.1007/s00213-020-05713-7 33219852 PMC10031567

[34] Brandon TH, Unrod M, Drobes DJ, . Facilitated extinction training to improve pharmacotherapy for smoking cessation: a pilot feasibility trial. Nicotine Tob Res. 2018;20(10):1189-1197. doi:10.1093/ntr/ntx203 29059409 PMC7868958

[35] Rose JE, Behm FM, Westman EC. Nicotine-mecamylamine treatment for smoking cessation: the role of pre-cessation therapy. Exp Clin Psychopharmacol. 1998;6(3):331-343. doi:10.1037/1064-1297.6.3.331 9725117

[36] Hawk LW Jr, Ashare RL, Rhodes JD, Oliver JA, Cummings KM, Mahoney MC. Does extended pre quit bupropion aid in extinguishing smoking behavior? Nicotine Tob Res. 2015;17(11):1377-1384. doi:10.1093/ntr/ntu347 25589680 PMC4612343

[37] Brown RA, Burgess ES, Sales SD, Whiteley JA, Evans DM, Miller IW. Reliability and validity of a smoking timeline follow-back interview. Psychol Addict Behav. 1998;12(2):101-112. doi:10.1037/0893-164X.12.2.101

[38] Peng AR, Morales M, Wileyto EP, . Measures and predictors of varenicline adherence in the treatment of nicotine dependence. Addict Behav. 2017;75:122-129. doi:10.1016/j.addbeh.2017.07.006 28728040 PMC5581992

[39] Novalen M, Chenoweth MJ, Zhao B, Hawk LW, Tyndale RF. Stability of varenicline concentration in saliva over 21 days at three storage temperatures. Nicotine Tob Res. 2022;24(2):270-274. doi:10.1093/ntr/ntab173 34460924 PMC9013001

[40] Tanner JA, Novalen M, Jatlow P, . Nicotine metabolite ratio (3-hydroxycotinine/cotinine) in plasma and urine by different analytical methods and laboratories: implications for clinical implementation. Cancer Epidemiol Biomarkers Prev. 2015;24(8):1239-1246. doi:10.1158/1055-9965.EPI-14-1381 26014804 PMC4526326

[41] Cox LS, Tiffany ST, Christen AG. Evaluation of the Brief Questionnaire of Smoking Urges (QSU-brief) in laboratory and clinical settings. Nicotine Tob Res. 2001;3(1):7-16. doi:10.1080/14622200020032051 11260806

[42] Hughes JR. Background on the Minnesota Tobacco Withdrawal Scale–Revised (MTWS-R). March 1, 2017. Accessed October 6, 2019. http://contentmanager.med.uvm.edu/docs/background/behavior-and-health-documents/background.pdf?sfvrsn=2

[43] Lerman C, Schnoll RA, Hawk LW Jr, ; PGRN-PNAT Research Group. Use of the nicotine metabolite ratio as a genetically informed biomarker of response to nicotine patch or varenicline for smoking cessation: a randomised, double-blind placebo-controlled trial. Lancet Respir Med. 2015;3(2):131-138. doi:10.1016/S2213-2600(14)70294-2 25588294 PMC4480925

[44] Ebbert JO, Hughes JR, West RJ, . Effect of varenicline on smoking cessation through smoking reduction: a randomized clinical trial. JAMA. 2015;313(7):687-694. doi:10.1001/jama.2015.280 25688780 PMC4883651

[45] Rennard S, Hughes J, Cinciripini PM, ; Flexible Quit Date Study Group. A randomized placebo-controlled trial of varenicline for smoking cessation allowing flexible quit dates. Nicotine Tob Res. 2012;14(3):343-350. doi:10.1093/ntr/ntr220 22080588 PMC3281242

[46] Anthenelli RM, Benowitz NL, West R, . Neuropsychiatric safety and efficacy of varenicline, bupropion, and nicotine patch in smokers with and without psychiatric disorders (EAGLES): a double-blind, randomised, placebo-controlled clinical trial. Lancet. 2016;387(10037):2507-2520. doi:10.1016/S0140-6736(16)30272-0 27116918

[47] Verplaetse TL, Morris ED, McKee SA, Cosgrove KP. Sex differences in the nicotinic acetylcholine and dopamine receptor systems underlying tobacco smoking addiction. Curr Opin Behav Sci. 2018;23:196-202. doi:10.1016/j.cobeha.2018.04.004 31341936 PMC6656369

[48] Jorenby DE, Hays JT, Rigotti NA, ; Varenicline Phase 3 Study Group. Efficacy of varenicline, an alpha4beta2 nicotinic acetylcholine receptor partial agonist, vs placebo or sustained-release bupropion for smoking cessation: a randomized controlled trial. JAMA. 2006;296(1):56-63. doi:10.1001/jama.296.1.56 16820547

[49] Niaura R, Hays JT, Jorenby DE, . The efficacy and safety of varenicline for smoking cessation using a flexible dosing strategy in adult smokers: a randomized controlled trial. Curr Med Res Opin. 2008;24(7):1931-1941. doi:10.1185/03007990802177523 18513462

[50] Tonkin SS, Colder C, Mahoney MC, . Evaluating treatment mechanisms of varenicline: mediation by affect and craving. Nicotine Tob Res. 2022;ntac138. Published online May 26, 2022. doi:10.1093/ntr/ntac138 35639828 PMC9596996

[51] Rose JE, Herskovic JE, Behm FM, Westman EC. Precessation treatment with nicotine patch significantly increases abstinence rates relative to conventional treatment. Nicotine Tob Res. 2009;11(9):1067-1075. doi:10.1093/ntr/ntp103 19567826

[52] Baker TB, Piper ME, Smith SS, Bolt DM, Stein JH, Fiore MC. Effects of combined varenicline with nicotine patch and of extended treatment duration on smoking cessation: a randomized clinical trial. JAMA. 2021;326(15):1485-1493. doi:10.1001/jama.2021.15333 34665204 PMC8527361

[53] Motschman CA, Gass JC, Wray JM, . Selection criteria limit generalizability of smoking pharmacotherapy studies differentially across clinical trials and laboratory studies: a systematic review on varenicline. Drug Alcohol Depend. 2016;169:180-189. doi:10.1016/j.drugalcdep.2016.10.018 27863344

[54] Rigotti NA, Pipe AL, Benowitz NL, Arteaga C, Garza D, Tonstad S. Efficacy and safety of varenicline for smoking cessation in patients with cardiovascular disease: a randomized trial. Circulation. 2010;121(2):221-229. doi:10.1161/CIRCULATIONAHA.109.869008 20048210 PMC4096941

[55] Tashkin DP, Rennard S, Hays JT, Ma W, Lawrence D, Lee TC. Effects of varenicline on smoking cessation in patients with mild to moderate COPD: a randomized controlled trial. Chest. 2011;139(3):591-599. doi:10.1378/chest.10-0865 20864613

