# Supplementary material for: Effect of Extending the Duration of Prequit Treatment With Varenicline on Smoking Abstinence: A Randomized Clinical Trial
Source: JAMA Netw Open. 2022 Nov 11;5(11):e2241731. doi: 10.1001/jamanetworkopen.2022.41731 (PMC9652761; doi:10.1001/jamanetworkopen.2022.41731)
Supplement: Supplement 1. — Trial Protocol and Statistical Analysis Plan [file jamanetwopen-e2241731-s001.pdf]

Trial protocol/Statistical Analysis Plan

University at Buffalo Institutional Review Board (UBIRB)
Office of Research Compliance | Clinical and Translational Research Center Room 5018
875 Ellicott St. | Buffalo, NY 14203
UB Federalwide Assurance ID#: FWA00008824

Complete Research Protocol (HRP-503)
HRP-503-Protocol-EVarQuit-Rev-2020-06-30.docx

Table of Contents

Template Instructions 1
1.0 Objectives 3
2.0 Scientific Endpoints 4
3.0 Background 4
4.0 Study Design 6
5.0 Local Number of Subjects 6
6.0 Inclusion and Exclusion Criteria 7
7.0 Vulnerable Populations 9
8.0 Eligibility Screening 10
9.0 Recruitment Methods 10
10.0 Procedures Involved 12
11.0 Study Timelines 22
12.0 Setting 22
13.0 Community-Based Participatory Research 23
14.0 Resources and Qualifications 24
15.0 Other Approvals 26
16.0 Provisions to Protect the Privacy Interests of Subjects 26
17.0 Data Management and Analysis 27
18.0 Confidentiality 29
A. Confidentiality of Study Data 29
B. Confidentiality of Study Specimens 31
19.0 Provisions to Monitor the Data to Ensure the Safety of Subjects 31
20.0 Withdrawal of Subjects 33
21.0 Risks to Subjects 34
22.0 Potential Benefits to Subjects 36
23.0 Compensation for Research-Related Injury 36
24.0 Economic Burden to Subjects 36
25.0 Compensation for Participation 36
26.0 Consent Process 38
27.0 Waiver or Alteration of Consent Process 41
28.0 Process to Document Consent 41
29.0 Multi-Site Research (Multisite/Multicenter Only) 42
30.0 Banking Data or Specimens for Future Use 42
31.0 Drugs or Devices 43
32.0 Humanitarian Use Devices 44

Template Instructions

Sections that do not apply:

- *In several sections, the addition of checkboxes for* ***Not Applicable*** *have been added to the template as responses.*
    - *If an N/A checkbox is present, select the appropriate justification from the list.*
    - *If an N/A checkbox is not present, or if none of the existing checkboxes apply to your study, you must write in your own justification.*
- *In addition:*
    - *For research where the only study procedures are records/chart review: Sections 19, 20, 22, 23, 24, 25, 31, and 32 do not apply.*
    - *For exempt research: Sections 31 and 32 do not apply.*

Studies with multiple participant groups:

- *If this study involves multiple participant groups (e.g. parents and children), provide information in applicable sections for each participant group. Clearly label responses when they differ. For example:*

Response:

Intervention Group:

Control Group:

Formatting:

- *Do not remove template instructions or section headings when they do not apply to your study.*

If you are pasting information from other documents using the "Merge Formatting" Paste option will maintain the formatting of the response boxes.

Amendments:

- *When making modifications or revisions to this and other documents, use the* ***Track Changes*** *function in Microsoft Word.*
- *Update the version date or number* ***on Page 3.***

PROTOCOL TITLE:

Include the full protocol title.

Response:

EVarQuit: Extinguishing cigarette smoking via extended pre-quit varenicline

PRINCIPAL INVESTIGATOR:

Name
Department
Telephone Number
Email Address

Response:
Larry W. Hawk, Jr., PhD
Department of Psychology
716-645-0192
lhawk@buffalo.edu

VERSION:

Include the version date or number.

Response: 2020-06-30

GRANT APPLICABILITY:

Indicate whether this protocol is funded by a grant (e.g. NIH, foundation grant). For a grant with multiple aims, indicate which aims are covered by this research proposal.

NOTE: This question does not apply to studies funded by a sponsor contract.

Include a copy of the grant proposal with your submission.

Response: All aims of NCI/NIH grant CA206193 are covered by this proposal.

App00-Grant-ExtinctionR01A1-NIHCompleteGrantDownload-2016-03-07.pdf.

RESEARCH REPOSITORY:

Indicate where the research files will be kept, including when the study has been closed. The repository should include, at minimum, copies of IRB correspondence (approval, determination letters) as well as signed consent documents. This documentation should be maintained for 3 years after the study has been closed.

Response:

Location: 3rd floor, Diefendorf Hall in locked cabinets within locked offices
Address: 311 Diefendorf Hall, S Campus, University at Buffalo
Department: Psychology

1.0 Objectives

1.1 *Describe the purpose, specific aims, or objectives of this research.*

Response:

Aim 1: Evaluate the impact of extended pre-quit varenicline therapy on smoking cessation.

Aim 2: Evaluate the impact of extended pre-quit varenicline therapy on smoking behavior and related processes prior to cessation.

Aim 3: Evaluate the degree to which changes in pre-quit smoking behavior (Aim 2) truly account for, or mediate, the impact of extended pre-quit varenicline on smoking cessation (Aim 1).

UPDATE 2020-06-30 – OPTIONAL SALIVA SAMPLE FOR GENETIC ANALYSIS: As detailed in our administrative supplement request (EvarQuit Admin Supp Application – 2020-04-21.pdf), "…to enhance this R01's ability to inform and link to precision medicine approaches, we propose to evaluate the role of variants in two families of genes previously associated with varenicline concentrations, nausea, and/or smoking cessation" (select SNPs related to drug transport [OCT2] and nicotinic receptor subunits [CHRNA4/CHRNB2]).

1.2 *State the hypotheses to be tested, if applicable.*

NOTE: A hypothesis is a specific, testable prediction about what you expect to happen in your study that corresponds with your above listed objectives.

Response:

We hypothesize that extended run-in varenicline will improve bio-verified continuous abstinence rates at end-of-treatment and at long-term follow-up (6- months), compared to the standard run-in. Because extended pre-quit treatment may be particularly helpful for women (Becker et al., 2008; Hawk et al., 2012), the study is powered to evaluate moderation of treatment by gender.

Consistent with extinction theory, we predict that the extended run-in group will exhibit greater pre-quit reductions in smoking (cigarettes per day, CO) than the standard run-in group. Effects on other biological (cotinine, total nicotine exposure), self-report (subjective effects of smoking, craving, withdrawal, nausea, expectancies), and behavioral (laboratory reinforcement task) outcomes will also be evaluated to better characterize potential treatment mechanisms.

The extinction model predicts that the cessation benefits of extended run-in varenicline will be explained by greater pre-quit reductions in smoking. We will also test whether this mechanism is particularly strong among women (i.e., moderated mediation).

2.0 Scientific Endpoints

2.1 Describe the scientific endpoint(s), the main result or occurrence under study.

NOTE: Scientific endpoints are outcomes defined before the study begins to determine whether the objectives of the study have been met and to draw conclusions from the data. Include primary and secondary endpoints. Some example endpoints are: reduction of symptoms, improvement in quality of life, or survival. Your response should ***not*** *be a date.*

Response:

Our primary outcome measure (for Aims 1 and 3) is bio-verified self-report of continuous abstinence from smoking during the final four weeks of treatment (Weeks 8-11 post-quit, the typical primary outcome in varenicline trials; e.g., Gonzales et al., 2006; Hawk et al., 2012; Jorenby et al., 2006). Continuous abstinence will also be evaluated for weeks 8-26 and 8-26 post-quit (with bio-verification at 6M visits, respectively). Secondary clinical outcomes include rates of side effects and pill count measures of adherence.

Our primary mediator of interest will be smoking behavior (cigarettes smoked per day, or CPD) during the pre-quit phase of the study (Weeks -5 through -1), as reported during daily morning EMA assessments. Secondary measures for understanding the causal process include expired-air CO and varenicline levels will also be examined, as will laboratory measures of reinforcement, craving, withdrawal, subjective effects of smoking, and nausea.

3.0 Background

3.1 Provide the scientific or scholarly background, rationale, and significance of the research based on the existing literature and how it will contribute to existing knowledge. Describe any gaps in current knowledge. Include relevant preliminary findings or prior research by the investigator.

Response: Cigarette smoking remains the leading preventable cause of death in the US, killing an estimated 480,000 people per year and linked to 1 in 3 cancer deaths. The 2014 Surgeon General's Report (US DHHS, 2014) suggests that the consequences of smoking are even worse than previously thought. Cigarette design changes have increased the risk smoking poses for lung cancer, and smoking is now linked to an even larger number of cancers (including colorectal and liver). Moreover, emerging evidence suggests that smoking increases the risk for cancer recurrence and treatment toxicity (US DHHS, 2014).

Although quitting smoking markedly lowers disease risk and improves health (US DHHS 2014), there are a limited number of effective smoking cessation therapies, and long-term cessation rates remain low. Three evidence-based pharmacotherapies are available for smoking cessation in the US: nicotine replacement therapy (NRT), bupropion, and varenicline. Both NRT and bupropion approximately double the odds of long-term cessation (6 months or more) when compared to placebo (e.g., Fiore et al., 2008). Varenicline, approved by the FDA in 2006, triples the odds of quitting compared to placebo and outperforms single-NRT and bupropion (for a review, see Cahill et al., 2013; c.f. Baker et al., 2016). Nevertheless, long-term abstinence rates remain low, with only 1 in 4 varenicline users smoke free 6+ months after quitting (Cahill et al., 2013).

Given the tremendous costs of smoking and the benefits of quitting, the development of cessation approaches with greater efficacy is critical. The typical drug discovery path is unlikely to generate an answer. Despite enormous investments in evaluating numerous medications in the decade since varenicline

reached the US market, "no new smoking cessation aid is nearing…FDA approval" (e.g., Rigotti, 2015). Certainly, there is nothing looking likely to do better than the treatments we already have – but that is exactly what we need.

To advance clinical practice, we build on basic research, theory, and compelling preliminary data from small-scale RCTs to evaluate a treatment approach that appears likely to beat our current "best in class" cessation approach, standard varenicline therapy. We take the perspective that improved understanding and targeting of treatment mechanisms is the best path forward (e.g., Kraemer et al. 2006; MacKinnon, 2008; Rigotti, 2015; TRIP, 1988). Varenicline binds to the alpha-4 beta-2 receptor subunit of nicotinic acetylcholine receptors (nAChR), exerting effects as both a partial nicotine agonist, by stimulating dopamine release, and as an antagonist, by blocking the binding of nicotine to this site. In clinical trials, varenicline robustly decreased post-cessation smoking cravings and satisfaction with cigarettes during lapses (e.g., Gonzales et al., 2006; Jorenby et al., 2006). For varenicline, which is typically administered for a week prior to quitting and is hypothesized to work partly by reducing the reinforcing effects of smoking (e.g., Rollema et al., 2007), it is also critical to examine pre-quit treatment mechanisms (e.g., Cummings & Mahoney, 2008; Fiore et al., 2008; Hawk et al., 2015; Rose, 2009, 2011; Rose & Levin, 1991).

From a learning perspective, when favorable consequences of a behavior are removed, the behavior decreases in frequency, or is extinguished. Consistent with the hypothesis that varenicline diminishes the reinforcing value of smoking in humans, varenicline dose-dependently reduces self-administration of nicotine in rats (O'Connor et al., 2010; Rollema et al., 2007). For varenicline to promote extinction in human smokers, they must continue smoking while taking the drug in order to learn that the reinforcing effects are attenuated. The typical one-week run-in period for varenicline is likely insufficient, as extinction requires numerous "trials" (see Bouton et al. 2012) and does not generalize well from one situation or context to another (Bouton, 2000, 2004a; Collins & Brandon, 2002; see also Conklin & Tiffany, 2002).

How can we effectively promote such extinction in smokers? The pioneering work of Rose et al. (1988) with the nicotine patch suggested a straightforward method: extend the pre-quit run-in medication period to allow greater repeated natural exposure to attenuated reinforcement from smoking prior to the target quit date (TQD). We recently tested this approach in small-RCTs of both bupropion (Hawk et al., 2015 /Prelim Study 1) and varenicline (Hawk et al., 2012 / Prelim Study 2; see also Ashare et al., 2012, Gass et al., 2012); treatment-seeking smokers were randomized to either a Standard run-in group (3 weeks of placebo, 1 week of pre-TQD medication) or an Extended run-in group (4 weeks of pre-TQD medication); all participants received counseling and typical regimens of post-TQD medication. Hajek et al. (2011) conducted an independent but very similar small-scale RCT of varenicline. In all three pilot RCTs, the results were consistent with an extinction hypothesis: the extended run-in resulted in greater reductions in smoking rate (and tended to reduce craving and smoking satisfaction) prior to the TQD and improved abstinence rates at short-term follow-up. (In both Hawk et al. studies, there was evidence that the extended run-in had a greater impact among women more than men; Hajek et al. did not examine gender effects). Overall, the results of these three studies are promising, particularly because the control condition was an approved cessation medication; in the case of varenicline, the extended run-in beat the current "best in class" treatment (standard run-in varenicline). However, the studies were limited by their small sample sizes (Ns=60-100) and short-term follow-up (4 weeks – 3 months).

The proposed study takes the next critical step in evaluating the degree to which extended-run in varenicline will set a new efficacy standard in smoking cessation, a large-scale RCT with long-term follow-up. Thus, the primary significance of the proposed RCT is that it will, if successful, provide a marked advance in the treatment of cigarette smoking, the leading preventable cause of death in the US. Moreover, it would do so at far less cost and far more quickly than traditional pharmaceutical development pathways (see Chong & Sullivan, 2007; Collins, 2011). Although every innovation in treatment faces challenges in bridging the science-practice gap, the proposed treatment and RCT are designed to facilitate and inform widespread dissemination and implementation (see the Introduction to the revision), further enhancing the significance of the work.

The proposed RCT will also evaluate the mechanisms by which extended run-in varenicline exerts its clinical effects. This addresses a critical split in our field and gap in our knowledge – whereas most varenicline RCTs have been weak in testing mechanisms with anything other than retrospective self-report at clinic visits (e.g., Gonzales et al., 2006; Jorenby et al., 2006; Ebbert et al., 2015), most laboratory

behavioral pharmacology studies of varenicline mechanisms have focused on participants who are not actively trying to quit (Mostchman et al., 2014). As in our pilot RCT with varenicline (Hawk et al., 2012; Prelim Study 2), we will obtain biochemical measures of changes in smoking behavior and real-world, real-time measures of smoking reinforcement and related constructs via ecological momentary assessment (EMA). In addition, we have adapted laboratory paradigms from behavioral pharmacology to more thoroughly evaluate changes in reinforcement in the pre-quit period (see Prelim Study 4). Most importantly, we will use these data to directly evaluate the degree to which extinction of reinforcement accounts for, or mediates, the effect of extended run-in varenicline therapy. Our evaluation of putative treatment mechanisms will both advance knowledge and provide clearer targets for subsequent treatment development and personalization (e.g., Kraemer et al., 2006). Additional details are provided in the attached grant application and administrative supplement application.

3.2 Include complete citations or references.

Response: All references are included in the attached grant application (App00) and administrative supplement application.

4.0 Study Design

4.1 Describe and explain the study design (e.g. case-control, cross-sectional, ethnographic, experimental, interventional, longitudinal, observational).

Response: This study, which will be conducted over a 5-year period, will employ a two-group, balanced, randomized, double-blind, placebo-controlled parallel-group design. The research design is summarized in the Figure at right.

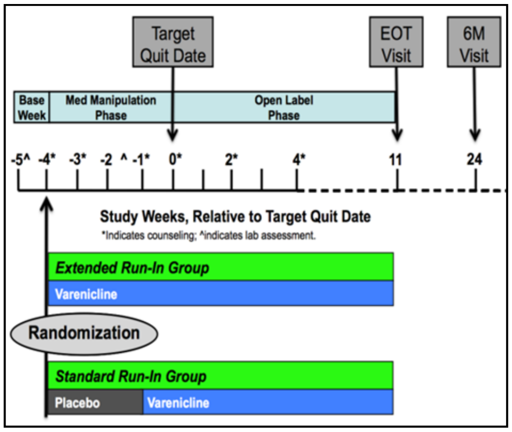

5.0 Local Number of Subjects

5.1 Indicate the total number of subjects that will be enrolled or records that will be reviewed locally.

Response: Participants will be 320 treatment-seeking adult smokers (160 female).

UPDATE 2020-06-30 – OPTIONAL SALIVA SAMPLE FOR GENETIC ANALYSIS: An optional saliva sample for genetic analysis will be obtained from up to 200 of the 320 participants.

5.2 If applicable, indicate how many subjects you expect to screen to reach your target sample (i.e. your screen failure rate).

Response: To accrue 320 ITT participants across 44 months, we have developed conservative projections for accruing 9 ITT participants per month: screening approximately 55 potential participants by phone each month, with 50% (28) of those eligible after phone prescreen, 50% (14) of those attending the intake visit, 75% (10) of those at intake consenting and remaining eligible after full screening, and 90% (9) of those eligible ultimately achieve ITT status.

2019-11 Update (approved/implemented early 2020-01): In recent months, we have extensively reviewed our accrual process. Accrual flow projections that we made in the grant proposal (e.g., 50% eligible on phone screen, 75% eligible at intake visit) have been accurate, with one critical exception. We

predicted that we would lose no more than 10% of prospective participants between the intake visit and beginning treatment 1-2 weeks later (and counting as one of our 320 intent-to-treat, or ITT, participants). In reality, we have lost more than twice that many people (21%) at this stage. Importantly, the loss of potential participants between the intake visit and ITT disproportionately affects the representation of racial/ethnic minorities. That is, participant loss at this stage is 36% for people from minorities compared to 15% for non-Hispanic Caucasians.

In an effort to improve the proportion of intake-eligible participants who ultimately achieve ITT status, we made the following changes with the 2019-11 IRB modification:

1. Increase remuneration for the lab visits (see Section 26.1), which provide no clinical benefit and last longer than clinic visits.

2. Eliminate the 50% adherence requirement for the device-initiated assessments in the Ecological Momentary Assessment (EMA; see section 11.1).

5.3 Justify the feasibility of recruiting the proposed number of eligible subjects within the anticipated recruitment period. For example, how many potential subjects do you have access to? What percentage of those potential subjects do you need to recruit?

Response: Roughly 20% of adults in NY smoke, and most smokers say they would like to quit. Thus, there is a large number of eligible participants in WNY. Indeed, our success enrolling 12 ITTs/month in a more demanding and restrictive trial (see Lerman et al., 2015) from 2011-2015 supports the feasibility of meeting our accrual target.

6.0 Inclusion and Exclusion Criteria

6.1 Describe the criteria that define who will be ***included*** *in your final study sample.*

NOTE: This may be done in bullet point fashion.

Response:

- Baseline smoking rate and CO:
    - Prior to 2019-11 modification: Smoking at least 10 cigarettes per day for the past 6 months and CO >7 at intake.
    - Beginning with the **2019-11 modification**: Smoking at least 5 cigarettes per day (CPD) for the past 6 months. (In November 2019, we noted that smoking 5-9 was the most single common reason for exclusion at phone screen. This was particularly notable for people from racial and ethnic minority groups [e.g., 2% of phone screens for non-Hispanic whites vs. 12% of phone screens for Black/African American participants] In light of the growing number of daily smokers who smoke <10 CPD [e.g., Jamal et al., MMWR, 2018) and our desire to increase the diversity and representativeness of the sample, we reduced the CPD criterion from 10 to 5. With lighter smokers, the expired air CO criterion would be less accurate/reliable for indexing daily smoking; therefore, it was eliminated.) Once the modification is approved, we will contact people who were previously deemed ineligible only because they smoked 5-9 CPD and offer to re-screen if they are interested.
- At least moderately motivated to quit smoking (3 or 4 on phone screen; modified MTSS) and intention to make a quit attempt with varenicline 1 month after treatment begins.
- Planning to remain in western NY during the study period (intake)
- Willing to use varenicline and to refrain from other cessation treatments and tobacco products during the study period. (intake)
- Age 18-70 years. (phone screen)
- Fluent English speaker (clinical judgment)
- Capable of providing informed consent, which includes compliance with the requirements and restrictions listed in the combined consent and HIPAA form (clinical judgment)

- To be ITT, the participant must complete Lab Visit 1 and meet minimal completion rate for real-world (EMA) assessments (detailed below; see also Section 6.2).
- Normal or corrected vision required for study.

6.2 Describe the criteria that define who will be ***excluded*** *from your final study sample.*

NOTE: This may be done in bullet point fashion.

Response:

- Use of other tobacco products, including e-cigarettes, in past 7 days (phonescreen)
- Use of smoking cessation medication, including nicotine replacement therapy, in the past 14 days? (phonescreen)
- Prior allergy/hypersensitivity to varenicline (phone screen)
- Pregnancy (phone screen, plus Urine at Intake)
- Substance use:
    - Alcohol: *At phone screen*: "Daily or almost daily" report of drinking 5 (4 for women) or more drinks a day in the past year (Nida Quick Screen after explaining drinks per day). *Intake*: AUDIT score > 15 at intake, suggestive of alcohol dependence and warranting treatment; for those with scores between 8 and 15, we will advise reducing drinking; Babor et al., 2001; see also Rubinsky et al 2010).
    - Medical treatment in past 3 months, including *Suboxone (buprenorphine) and methadone (at* phone screen)
    - Using a combination of the NIDA-modified ASSIST (4-26 = moderate risk; 27+ = high risk) and urine toxicology screen (both at intake):
        - Cannabis: ASSIST=27+ (tox screen not used)
        - Cocaine: ASSIST=7+ OR positive tox screen
        - Methamphetamine: ASSIST=7+ OR positive tox screen
        - Inhalants, hallucinogens: ASSIST score = 7+
        - Prescription stimulants, sedatives, or sleeping pills:
            - With prescription, ASSIST 27+
            - Without prescription, ASSIST 7+
        - Opioids:
            - With prescription, ASSIST 27+ (note ineligible if prescription is for buprenorphine or methadone)
            - Without prescription, ASSIST 7+ OR positive tox screen

- Psychiatric:
    - Antipsychotic medications (phone / intake)
    - Lifetime history of schizophrenia or bipolar disorder (phone)
    - Evidence of *current major depression (per* Patient Health Questionnniare (PHQ-9; Kroenke & Spitzer, 2002) score 12+, see Gilbody & McMillan, 2012; Loewe et al, 2004) at intake
    - Past 10 years suicidal ideation / behavior at intake, using slightly more conservative exclusion criteria than in the EAGLES study of neuropsychiatric events when quitting smoking (see Anthenelli et al., 2016), all of the following are exclusionary on the baseline Columbia-Suicide Severity Rating Scale (Posner et al., 2008):
        - SI without intent (C-SSRS #1, #2, or #3), if any intensity rating (Frequency, Duration, Controllability, Deterrents, or Reasons for Ideation) is > 2.
        - SI with intent (C-SSRS #4, or #5), regardless of intensity ratings.
        - Suicidal Behavior (any suicide attempt, interrupted attempt, aborted attempt, or suicide preparatory acts or behavior on the C-SSRS).
- General Exclusion:
    - Any medical condition, illness, disorder or concomitant medication that compromises participant safety or treatment, as determined by the Principal Investigator and/or Study Physician.

- Inability to provide informed consent or complete any of the study tasks as determined by the Principal Investigator and/or Study Physician.

6.3 *Indicate specifically whether you will include any of the following special populations in your study using the checkboxes below.*

NOTE: Members of special populations may not be targeted for enrollment in your study unless you indicate this in your inclusion criteria.

Response: N/A – We will not include any of the following special populations.

☐ Adults unable to consent
☐ Individuals who are not yet adults (infants, children, teenagers)
☐ Pregnant women
☐ Prisoners

6.4 *Indicate whether you will include non-English speaking individuals in your study.* ***Provide justification if you will exclude non-English speaking individuals.***

In order to meet one of the primary ethical principles of equitable selection of subjects, non-English speaking individuals may ***not*** *be routinely excluded from research as a matter of convenience.*

In cases where the research is of therapeutic intent or is designed to investigate areas that would necessarily require certain populations who may not speak English, the researcher is required to make efforts to recruit and include non-English speaking individuals. However, there are studies in which it would be reasonable to limit subjects to those who speak English. Some examples include pilot studies, small unfunded studies with validated instruments not available in other languages, studies with numerous questionnaires, and some non-therapeutic studies which offer no direct benefit.

Response: We will *not* include non-English speaking individuals because the study focuses on extensive self-report measures, including 35 days of daily assessments with electronic reporting, that have not validated in languages other than English.

7.0 Vulnerable Populations

If the research involves special populations that are considered vulnerable, ***describe the safeguards included to protect their rights and welfare.***

NOTE: You should refer to the appropriate checklists, referenced below, to ensure you have provided adequate detail regarding safeguards and protections. You do not, however, need to provide these checklists to the IRB.

7.1 *For research that involves* ***pregnant women,*** *safeguards include:*
NOTE CHECKLIST: Pregnant Women (HRP-412)

Response:

☒ **N/A**: This research does not involve pregnant women.

7.2 *For research that involves* ***neonates of uncertain viability or non-viable neonates,*** *safeguards include:*
NOTE CHECKLISTS: Non-Viable Neonates (HRP-413), or Neonates of Uncertain Viability (HRP-414)

Response:

☒ **N/A:** This research does not involve non-viable neonates or neonates of uncertain viability.

7.3 *For research that involves* ***prisoners****, safeguards include:*
NOTE CHECKLIST: Prisoners (HRP-415)

Response:

☒ **N/A:** This research does not involve prisoners.

7.4 For research that involves ***persons who have not attained the legal age for consent to treatments or procedures involved in the research ("children")****, safeguards include:*
NOTE CHECKLIST: Children (HRP-416)

Response:

☒ **N/A:** This research does not involve persons who have not attained the legal age for consent to treatments or procedures ("children").

7.5 For research that involves ***cognitively impaired adults****, safeguards include:*
NOTE CHECKLIST: Cognitively Impaired Adults (HRP-417)

Response:

☒ **N/A:** This research does not involve cognitively impaired adults.

7.6 Consider if other specifically targeted populations such as students, employees of a specific firm, or educationally or economically disadvantaged persons are vulnerable. ***Provide information regarding their safeguards and protections, including safeguards to eliminate coercion or undue influence.***

Response:

☒ **N/A** This study does not target vulnerable populations.

8.0 Eligibility Screening

8.1 Describe ***screening procedures*** *for determining subjects' eligibility. Screening refers to determining if prospective participants meet inclusion and exclusion criteria.*

Include all relevant screening documents with your submission (e.g. screening protocol, script, questionnaire).

Response:

After obtaining verbal consent, potential subjects will be screened by telephone for eligibility to attend an overview and intake visit.

EVQ2CRFs - Initial Screen - 2017-08-02.pdf(2017-08-02)

The intake will begin with informed consent. Consented participants will complete the following assessments: vital signs and CO levels, smoking history, concomitant medication review, baseline side effects, urine toxicology screen, urine pregnancy test for women, and brief measures of mood, suicidality, depression, and anxiety. A study clinician will carefully review the medical history and complete a focused physical examination.

Informed consent is App28-InformedConsentWithHIPPA—EvarQuit.

Measures are found in:

- EVQ2CRFs - Intake - Self-Report Measures - 2017-08-02.pdf(2017-08-02)
- EVQCRFs - Intake - Staff Instruments - 2017-08-02.pdf(2017-08-02)

During the baseline lab visit, participants will complete the laboratory reinforcement task (discussed below) and will be trained in completing a baseline week of ecological momentary assessments (EMA; discussed below). Participants must attend the baseline lab visit and complete at least 40% of baseline week (EMAs) in order to continue in the study. (Participants who do not meet these requirements can schedule one additional baseline week.)

9.0 Recruitment Methods

☐ **N/A:** This is a records review only, and subjects will not be recruited. NOTE: If you select this option, please make sure that all records review procedures and inclusion/exclusion screening are adequately described in other sections.

9.1 Describe when, where, and how potential subjects will be recruited.

NOTE: Recruitment refers to how you are identifying potential participants and introducing them to the study. Include specific methods you will use (e.g. searching charts for specific ICD code numbers, Research Participant Groups, posted advertisements, etc.).

Response:

We plan to enroll participants from January 2017 through January 2021.

Community participants will be recruited primarily via radio and television ads, internet (e.g., Craigslist, Facebook), flyers around the community and via email list serves, and newspaper advertisements, as in our recent large-scale trial. We also plan to use researchmatch.org, Urban Family Practice, and the Buffalo Research Registry.

The Urban Family Practice will *send a co-signed letter out to their patients, providing them more information about the EvarQuit Program. Should a person become interested in the program, they could call us for more information. Additionally, names and phone numbers of those to whom letters were sent will be provided to our team. We will call those participants that we have not heard from within 2 weeks of letter postmark date to see if they received the information (following the attached script). We will only make two contact attempts by phone to each person.*

We will also use I2B2 in UB CTSI to recruit participants from the UBMD medical data base. A letter will be sent to the Physician (see attached letter) prior to contacting participants (see attached letter). We will not contact prospective participants by phone, allowing them greater control over their participation.

The New York State Department of Health has approved of the New York State Smokers Quit Line (NYSSQL) sending out letters to smokers in our region who recently contacted the quit line. We will not have access to any names or any contact information. We will not be cosigning the letter. Folks who get the letter will have the option of contacting us for more information.

A project website also allows potential participants to find our information and contact us, if they are interested in being screened for our program (see attached screen shots & http://quitforgoodwny.com/).

9.2 Describe how you will protect the privacy interests of prospective subjects during the recruitment process.

NOTE: Privacy refers to an individual's right to control access to him or herself.

Response: Most importantly, we will recruit via public advertisements so that interested participants self identify, and we will only contact participants and collect study data by methods to which they have requested/consented. To enhance privacy and confidentiality during the phone screen, all phone screens will be conducted from secure offices on the third floor of Diefendorf Hall, and messages will be quite general (see phone script overview, App08-01a).

9.3 Identify any materials that will be used to recruit subjects.

NOTE: Examples include scripts for telephone calls, in person announcements / presentations, email invitations.

For advertisements, include the final copy of printed advertisements with your submission. When advertisements are taped for broadcast, attach the final audio/video tape. NOTE: You may submit the wording of the advertisement prior to taping to ensure there will be no IRB-required revisions, provided the IRB also reviews and approves the final version.

Response:

EvarQuit Study Facebook Recruitment

EVQ Recruitment Letter-Urban Family Practice

- EvarQuit Advertising Flyers- provides several flyer versions for the study.
- Referral Cards
- Website Screen shots- for www.QuitforGoodWNY.com
- Recruitment Letter to Participants from NYSSQL
- App09-01-EvarQuit TV & Radio Advertising 2018-12-13.doc provides the text for radio, craigslist, and newspaper advertisements.
- App09-02a-I2B2 Physician permission Letter Template ver 032417 - EVarQuit2017-06-01.docx
- App09-02b-I2B2 Recruitment letter to participant template ver 032417 - EvarQuit 2017-06-01.docx
- App09-03– Phone and Letter Script for Prior CPD Ineligible.docx

10.0 Procedures Involved

10.1 Provide a description of ***all research procedures or activities*** *being performed and when they are performed once a subject is screened and determined to be eligible. Provide as much detail as possible.*

NOTE: This should serve as a blueprint for your study and include enough detail so that another investigator could pick up your protocol and replicate the research. For studies that have multiple or complex visits or procedures, consider the addition of a schedule of events table in in your response.

Response: Procedures. Study procedures generally follow standard practice and our prior work.

Lab visits. In addition to the measures noted in the table above, the two lab visits include two additional procedures, described below. During the baseline lab visit (L1; Week -5), participants will complete the laboratory reinforcement task (CBUCC; see below) and will be trained in completing a baseline week of EMA assessments (details below). Lab visit 2 (L2; Week -2) is completed in the final week of the medication manipulation phase and offers clear experimental data regarding the impact of varenicline versus placebo on the laboratory task (CBUCC).

- For both CBUCC and EMA procedures, see EvarQuit Lab 1 SOP – 2017-09-19.docx.
- For L2 procedures including the ad-lib period, see EvarQuit Lab 2 SOP – 2019-02-18.docx
- For questionnaires used at the Lab Visits, see the EVQ2 – CRFs – Lab Visit.pdf.

CBUCC. Participants will be instructed to arrive at the lab visits having not smoked since midnight; most sessions will be scheduled in the morning to provide modest overnight abstinence from smoking (expired-air CO must be at least 40% lower than the CO obtained at intake or the session will be rescheduled). During CBUCC (Gass & Tiffany, under review), smokers are exposed to a lit cigarette, a cup of water, or a portion of highly preferred food (12 trials each, counterbalanced). These stimuli are located behind a movable glass door. On each trial, smokers rate their craving in the presence of the cue and then indicate the amount of money they are willing to spend to gain access to the cue. *During the task, they will be video recorded. This video recording will be used to examine behavior during the lab task via coding paradigm..* Participants are given $10 at the beginning of the procedure and told they can keep whatever amount they do not spend during CBUCC. Participants may spend $.01 to $.25 on each trial. The more the participants spend, the greater the probability that the door will be unlocked and they will be able to sample the cue on that trial (probabilities range from 5% to 95%). At each trial, participants are shown the CBUCC choice screen. After deciding how much to spend, participants are told to try to open the door. If the door is unlocked, they can sample the cue (1 cigarette puff, sip of water, or bite of food).

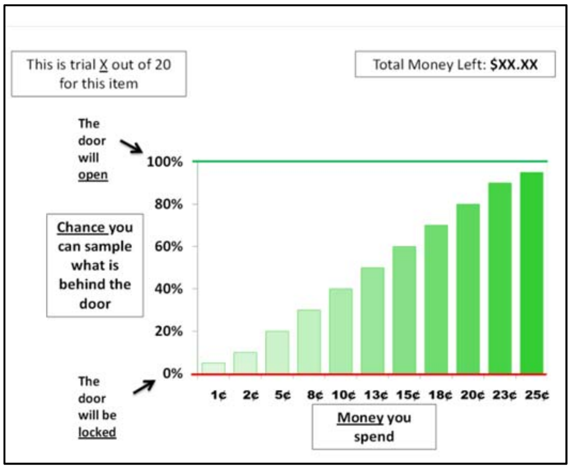

CBUCC generates multiple indices of reinforcement, including self-reported craving, the amount of real money paid for the opportunity to puff a cigarette, latency to attempt to open the door, and actual consumption (observed puff duration). In contrast to conventional laboratory assessments of cigarette choice in which smokers delay smoking a single cigarette in exchange for money (e.g., McKee et al., 2012), CBUCC generates multiple indices of smoking motivation and reinforcement and does so across numerous trials. Thus, CBUCC produces very reliable estimates of these variables within a single laboratory session. Moreover, unlike conventional cigarette choice procedures, CBUCC allows us to examine the relative reinforcing value of consumable reinforcers.

Ad-Lib Cigarette, Food, and Water Period. Following CBUCC administration at L2 (not L1), we will now offer participants a brief ad-lib consumption period. Typically, participants have at least 8 cigarettes left after CBUCC, and there is often leftover food/water from the procedure that is typically discarded. Participants will have 15 minutes and can choose how much, if any, cigarettes, food, and water they wish to consume. During this period, they will be informed that research assistants have tasks to complete in the control room , and that they have 15 minutes to consume as much or as little as they wish. Participants will continue to be passively recorded during this period. We are expecting to use data from this period (i.e., count of cigarettes smoked, weight of food eaten, puff number/puff duration/interpuff interval of cigarettes) as an additional naturalistic measure of consumption reinforcement against which to measure the behaviors obtained during CBUCC. Based on the average times of L2 observed thus far, we do not expect that this will significantly lengthen the session on average from what is described in the consent (i.e., 2 hours). If they smoke or eat at all, they will also complete the Subjective Effects Questionnaire (same questions answered in CBUCC/on EMA).

UPDATE 2020-06-12 - elimination of lab visits for remaining participants:

The COVID lockdown obviously took a toll on all clinical research, and now we are modestly behind in our accrual. Moreover, there is the concern that, as New York slowly reopens, community members will be especially wary of trials such as ours because of the many visits to campus. Finally, there's always the possibility of additional lockdowns if COVID rebounds in the fall. We have reviewed our trial components for opportunities to reduce the number of visits and amount of unnecessary exposure

to procedures participants might find most burdensome or concerning – without affecting the overall design of the study or our ability to realize the specific aims of the project.

With the approval of our funding agency (the National Cancer Institute), this modification requests that we eliminate the two laboratory assessments of smoking reinforcement for the following reasons:

- They are the two longest study visits, and they are purely for research purposes, with no therapeutic component.

- The lab task employed in these visits requires participants to repeatedly receive cigarettes, food samples, and glasses of water from the researcher. Even thought we take all COVID-19-related precautions to reduce the risk of virus transmission, participants may still be concerned/distressed about the possibility of contracting the virus during this procedure.

- Although we value the mechanistic data that comes from the lab visits, these data are secondary to our primary outcomes (smoking cessation outcomes, ecological momentary assessments (EMA), and biochemical measures of smoking exposure. The core study design (a double-blind, placebo-controlled trial with follow-up through 6 months post-quit) is unaffected.

Training for the EMA will be moved from Lab Visit 1 to the end of the Intake Visit.

Ecological Momentary Assessment (EMA). Daily EMA data will be collected for 5 weeks before TQD through 4 weeks post TQD (i.e., Weeks -5 to +4) using an application (app) that can be loaded onto the participants' personal smartphone/tablet (significantly reducing participant burden; e.g., Ginexi et al., 2014) or onto a study cell phone or tablet provided to the participant. The app – mobile EMA (mEMA; http://mobileema.com; ilumivu, Inc.) allows de-identified data (linked only to participant ID) to be synchronized with a secure server. EMA training and assessment procedures will follow our recent work (Gass et al., 2012; Hawk et al., 2012). During the baseline lab visit, participants will be trained on proper use of the app and will demonstrate the ability to complete both self-initiated assessments (questionnaires that the participant initiates, e.g., morning assessment) and device-initiated assessments (alerts to participant are provided on a pseudo-random basis). Participants will be informed that they will receive $1 for completing each morning assessment and $1 for completing each device-initiated assessment. To improve adherence and reduce burden/intrusiveness, participants will select a 12-hour period during which EMA prompts occur each day. Participants will be contacted by research staff ~3 days into the baseline week to review adherence (based on incoming synchronized data) and troubleshoot any problems.

Morning assessment. Participants will be instructed to complete the morning assessment before smoking their first cigarette of the day and within one hour of waking. In addition to reporting total number of cigarettes smoked during the previous day (CPD), participants will report wake time and medication adherence on the previous day. They will also complete a brief measure of craving, withdrawal, or nausea/appetite. Altering the domain of assessment reduces both burden and reactivity, and counterbalancing will ensure adequate coverage of all domains across the pre-quit period. At the end of this assessment, the participants will be thanked for their report and reminded of the remuneration earned. In our pilot work, most participants completed far more (mean=94%) than the 43% (3 days per week) minimum required (Hawk et al., 2012).

Device-initiated assessments. Device-initiated prompts will be delivered 4 times per day (e.g., randomized within 3-hour blocks) to assess time since last cigarette and two of the following four domains: subjective effects of smoking, craving, withdrawal, and nausea/appetite (see Measures for details). Thus, each domain will be assessed up to twice per day. Counterbalanced presentation of domains across device-initiated assessments will maximize coverage of time of day and distribution of domains across each week, while reducing participant burden and reactivity compared to assessing every domain in each device-initiated assessment. Sessions end with a reminder about remuneration earned. In our pilot work, participants completed far more than the 50% minimum required (mean = 89%; Gass et al 2012).

2019-11 update: Note that in the 2019-11 IRB modification, we eliminated the 50% minimum (see also Section 6.2) for the device-initiated prompts. Review of data for participants already screened for the study demonstrated that participants had little trouble completing the morning assessments. This is

important, as the morning assessment contains measures central to the aims of the project. The device-initiated assessments, which are less critical to the aims of the project, were associated with greater challenges for participants, and roughly 10% of participants either had to complete a second week of baseline or never met the 50% baseline adherence threshold and were excluded or withdrew. To more efficiently complete the trial, we eliminated the 50% requirement for device-initiated assessments. We do, of course, continue to encourage and remunerate completion of these assessments.

UPDATE 2020-06-30 – OPTIONAL TROUBLESHOOTING SOFTWARE: As detailed above, participants are required to complete a minimum number of EMA assessments during the baseline week. Even after the baseline week, missed assessments reduce both the data available to the project and remuneration to participants. Consequently, it is important to quickly resolve any problems participants have with the EMA app. To date, troubleshooting has generally required lengthy phone conversations and/or ad hoc participant visits to the clinic for assistance. To minimize participant burden and more quickly address technical problems with the EMA app, participants will be offered (at Intake) the option install a free, HIPPA-compliant app (TeamViewer, https://www.teamviewer.com/en-us/) for troubleshooting. Participants have the option to decline having this app installed on their device and, if it is installed, participants can remove it at any time.

It will be explained to participants that, should they choose to install the TeamViewer app:

- TeamViewer allows research staff to, with the participant's consent, view and remotely control the participant's smartphone for a remote troubleshooting session.
- TeamViewer will only be used for troubleshooting EMA problems. During any troubleshooting session, research staff will use TeamViewer only to navigate into the EMA app and related settings to address problems and demonstrate how to avoid further issues, all while the participant observes the staff member's actions.
- Staff cannot use the app without real-time consent (the participant must click a pop-up to start a session). The participant can continue monitor their phone and view what is being accessed. If the participant chooses, they can end the remote session at any time.
- TeamViewer does not collect or store any information.

For questionnaire items, see App10.3-04-Questionnaires.

Study-Within-A-Trial (SWAT) on EMA Remuneration – added to protocol on 2018-12-06. Pending funding from the Buffalo CTSI Pilot Studies program, we plan to conduct a SWAT to evaluate methods to improve adherence Adherence in the current trial has not been as strong as in the pilot work reported above, likely in part because the pilot study was shorter (only 5 weeks of EMA instead of 9). In the current study, adherence has been strong at the outset but then dropped, with rates at their lowest during the critical 4-week post-quit period. We also observe that adherence is lower among African-Americans than among Caucasians. The goal of the SWAT is to evaluate two potential methods for improving adherence during the post-quit period : increased frequency of payment (from once every two weeks to three times per week) and increased amount of payment (from $1/assessment to $2/assessment.

In order to complete the EMA SWAT, over the course of a one-year period participants (equal numbers of male and female, and of Caucasian and African-American) will be randomly assigned to each of three conditions in advance of the TQD until we accrue 20 participants in each condition. At the TQD, participants in the SWAT will receive a written consent addendum.

For the three versions of the addendum, see EvarQuit Addendum – EMA SWAT – 2018-12-06.doc.

Participants randomly assigned to the standard condition would simply be informed that it is important to continue the EMA over the next four weeks. Participants randomly assigned to the increased frequency of payment and increased amount of payment will have the opportunity to accept or decline the modified payment plan. If they decline, they will simply continue to participate in EVarQuit, but will be not be enrolled in the SWAT.

We chose individualized addenda over a single broad consent form because the SWAT will only pertain to a subset of EVarQuit participants. We considered explaining all three conditions to SWAT

participants, but ultimately decided against it because doing so could cause unnecessary distress to participants randomized to the standard condition (who might feel they were ‘losing out’ even though nothing had changed).

The results of the SWAT will inform our decision of whether to alter remuneration for subsequent participants in the EVarQuit project.

Clinic visits (Weeks -4, -3, -1, 0 [TQD], 2, and 4), randomization, and study medication. At Clinic Visit 1 (Week -4), participants are randomized (within gender) to the extended or standard run-in group, complete study measures (see below), receive brief behavioral counseling (see below) and study medication (details below), and are instructed to begin the medication the next day. Subsequent study visits are similar in process and content. *Randomization.* The study statistician (Co-I Dr. Colder) will implement and monitor the small-urn randomization (within-gender in urns/blocks of 8 [4 extended run-in, 4 standard run-in]), leaving remaining personnel blinded to group membership. Participants are considered part of the intent-to-treat (ITT) sample once they are dispensed medication at Clinic Visit 1 (C1).

Study medication. At visit C1 (Week -4), participants will be provided an initial 1-week supply of study medication (either varenicline or identical appearing placebo) and instructed on use (one 0.5 mg tablet orally daily x 3 days, then one 0.5 mg tablet twice daily x 4 days, then two 0.5 mg tablets twice daily). One week prior to TQD (Visit C3 / Week -1), participants assigned to placebo will be switched over to varenicline with standard dose increases during the initial week of use. During the pre-quit period, all study medication (active & placebo) will be dispensed as 0.5 mg tablets. This approach was successfully used in Hawk et al. (2012) and will facilitate switching over from placebo to active medication while maintaining blinding. From TQD through EOT, all participants will receive open-label varenicline (one 1.0 mg tablet twice per day).

Pfizer will provide varenicline and matching placebo for the study at no cost. Should this change, or should we run low on study medication between shipments from Pfizer, the research pharmacy will produce matching opaque capsules containing varenicline (which they can purchase in bulk) and placebo (methylcellulose), as they have done in many prior studies.

Instructions for medication use will be reviewed at each clinic visit. Subjects will return any unused medication at the following clinic visit and will be dispensed enough medication to last until the next visit.

EVQ2CRFs – Intake (self-report and staff) pdfs include our side effect checklist from our previous trial, supplemented with additional screening using the Columbia Suicide Severity Rating Scale (CSSSRS). Our emphasis will be on detecting, addressing, and reporting symptoms that are new or increase from baseline. Consistent with App10-02, any new or increased suicidal ideation or behavior will be evaluated by Drs. Hawk, Tiffany, or Mahoney (all study PIs are either clinical psychologists or physicians trained in conducting further evaluation); the study PIs will make external (non-study) referrals for additional evaluation or treatment as clinically indicated.

Counseling. As in our prior work (Hawk et al., 2012), participants will receive brief individual behavioral counseling at 6 clinic visits (Weeks -4, -3, -1, 0 [TQD], 2, and 4) from counselors blind to treatment group. Pre-quit sessions focus on topics common in behavioral counseling, including honing the motivation to quit, identification of smoking triggers and trigger management, and social support (e.g., Abrams & Niaura, 2003; Fiore et al., 2008), without explicitly discussing extinction. However, to allow extinction to occur, we will not include active nicotine fading as part of the counseling. Instead, participants will be asked to follow their smoking urges, smoking at least 25% of their baseline rate to allow their bodies time to adjust to the medication, as in prior extended pre-quit work (Hawk et al., 2012, 2015; Rose et al., 1998). In response to feedback from participants in prior studies, we will also offer brief counseling “check-ins” by phone 1 and 7 weeks post-TQD.

App10-03a-EVQ2 Counseling SOP 2017-09-21.docx provides the Counselor Manual and App10-03b-EVQ2 Counseling Handouts 2017-09-21.docx provides the participant workbook.

End-Of-Treatment (EOT) and 6-months post-TQD (6M) visits allow for biochemical verification of self-reported abstinence, the primary outcome measure. Retention has been strong in our prior varenicline studies (Hawk et al., 2012; Lerman et al., 2015). Clear explanations of the importance of

follow-up data for clinical application, ongoing contact with participants (including reminder calls), and increased remuneration for attendance at EOT and 6M visits (when medication is no longer being provided) bolster retention, which is expected to be 95% at TQD, 82% at EOT, and ~75% at 6M (based on Hawk et al., 2012 and the Buffalo varenicline arm data from Lerman et al, 2015).

Update 2019-11: Retention at EOT and 6M follow-up has been substantially lower than anticipated. We believe this is related to several inter-related factors. First, because of the EMA remuneration (up to $35/week), remuneration at Clinic Visits is much higher than in our prior studies. Second, remuneration at EOT and 6M is actually somewhat lower than in our prior work – we did this in an effort to stay under the $600 threshold at which a 1099 would have to be issued, requiring participants to provide us with their SSN. We now re-balance the remuneration by shifting some remuneration from Clinic visits (which already have greater value because of the treatment received and the EMA remuneration) to the follow-up period (see Section 26).

In addition, we have observed that many participants do not answer the reminder calls for follow-up visits, and when they do not answer the phone call they are very likely (70-90%) to miss the subsequent follow-up. After extensive discussion with staff, and consideration of anecdotal information from participants, we will replace the reminder calls with brief (1-2 questions) REDCap surveys (see App 11.1 – Template for REDCap Follow-Up Survey) delivered via a text message link to the participant's phone. We have designed a plan we believe offers multiple advantages:

1) Rather than requiring that a participant answer our phone call at a specific time, participants can respond to a REDCap survey whenever they are available. Because participants have already completed 9 weeks of electronic assessments, this should be convenient and low-burden.

2) REDCap allows us to automate reminders to participants' who do not respond to the initial text of a survey link. We will send up to 3 reminders for each survey.

3) In contrast to phone calls, which could be associated with shame or embarrassment for participants who report relapsing to smoking, the REDCap surveys allow participants to report electronically without direct mention of the behavior to study staff.

4) Because the surveys are so brief, we can actually have more frequent contact with participants during the follow-up period (1, 3, and 5 weeks before EOT and 4 and 8 weeks before 6M follow-up), which should enhance retention rates.

Participant Satisfaction Surveys (C4 and EOT) Participants will be given satisfaction surveys at Clinic visit #4 (C4) and the End of treatment (EOT) visits. These surveys will be distributed in an unsealed enveloped. The participant will be asked to complete the survey on paper, in private, to answer honestly, and to seal the survey into the envelope after completion. Surveys will be delivered to Dr. Hawk; research assistants will not read the surveys of their participants. The C4 survey was not completed by those who had C4 before the 2019-05 modification. The EOT survey was mailed to participants who completed EOT prior to approval of the 2019-05 modification. Please see Satisfaction for EVQ 2019-05-02.docx.

Update 2020-04 – COVID-19 impact: Anecdotally, our smoking cessation trial participants have reported varied impact of COVID-19 on their quit smoking efforts. To more formally collect qualitative and quantitative data regarding the impact of COVID-19, we added a questionnaire (App - COVID-19 Quitting Questions_v3.5 - EVarQuit 2020-04-20.docx) to be administered once per participant. The COVID-19 questionnaire will be assessed at each participant's end-of-treatment (EOT) (remote) visit. If the person has already passed the EOT appointment, but has not yet reached the 6-month visit, we will ask them to complete it at the 6-month (remote) visit. The questionnaire is completely optional. The questionnaire would be employed until the stay-at-home order is lifted or all currently enrolled participants reach the 6-month milestone or government-mandated social distancing measures are eliminated in New York, whichever is later.

Update 2020-5-21 – Further assessing COVID-19 impact: The EvarQuit project was forced to implement changes to the provision of treatment due to the COVID-19 pandemic. One of the primary changes involved the transition from in-person counseling to remote counseling via Zoom software (when possible) or phone calls. In order to understand the opinions and experiences of participants in the EvarQuit project who completed at least one at-home counseling session as a result of the COVID-19

pandemic, we will be conducting voluntary individual interviews with about 25 currently enrolled participants. The goal of these one-on-one structured interviews is to improve the quality of the remote visits and enhance the subjective experience of our participants. Trained research assistants will contact participants by phone as close as possible following the Clinic 6 visit to introduce the interview. If the participant agrees to the procedures and provides verbal consent, the interview will be audio recorded so it can be coded by independent staff members. Audio recordings, labeled only with a participant number, will be stored on our secure server, and will be used to generate written transcripts for qualitative analyses. We will keep the audio recording for up to 6 months as they will be used to clarify information in the transcripts and will help to clarify the context of information obtained during interviews. Participant responses will remain anonymous.

Update 2020-05-29: COVID-19-related procedural changes: Per the UB Human Studies guidance 05212020.docx, the following procedural changes will be implemented in an effort to minimize transmission of the virus:

Engineering Measures:

- The clinic hallway (3rd floor in Diefendorf Hall) is approximately 11 ft wide; taped lines will be placed 2.5 ft from either wall all of the way down the hallway. People moving west to east will walk down one side of the hallway and those moving east to west will walk down the other side of the hallway. This will ensure that a distance of > 6ft can be maintained in the hallway at all times.
- Participants typically sit in chairs located in the main hallway of the clinic to wait for their appointment to start. The chairs will be removed from the hallway and participants will be escorted to a private interview room upon arrival to the clinic.

Administrative Measures:

- Staff will be asked to enter the clinic using the elevator OR the stairwell on the east end of the building and to leave using the stairwell on the west end. Staff arrival and departure times will be staggered as well to minimize stairwell traffic.
- Staff will be asked to wash their hands thoroughly and often, including immediately upon arrival, using CDC guidelines and to avoid touching their face.
- Room occupancy will be limited to maintain distances of at least 6 feet between staff and research participants except for brief procedures (such as blood pressure), during which staff will wear gloves, face mask, and eye protection (consistent with UB Human Studies guidance 05212020.docx).
- Signage outside the elevator and in the hallway will inform participants regarding the above measures as well as the need to have only one person on the elevator at a given time.

Prescreening of Research Participants:

As per the UB Human Studies guidance 05212020.docx, the following will be done prior to all participant visits:

During the reminder call the day before a visit:

- Participants will be asked to take their temperature; if they don't have a thermometer, they will be asked whether they feel feverish.
- Participants will be asked about the presence of any COVID-19 symptoms including: fever, cough, shortness of breath, sore throat, muscle aches, headache, new loss of taste or smell, and repeated or shaking chills (as noted on page 2 of the UB Human Studies Guidance 05212020.docx).
- Anyone known to be COVID-19 positive or who exhibits COVID-19 symptoms will be restricted from enrollment / attending in-person visits until symptom free and at least 14 days since date of diagnosis. For enrolled participants, remote visits (telemedicine) will be scheduled in the interim as the health of the participant allows.

Before leaving home on the day of a visit

- Participants will be asked to take their temperature at home.
- Participants will be asked to report any new symptoms on the day of the visit to the project coordinator prior to coming to the clinic/lab.
- Participants will be asked to wear a face covering prior to entering the building; if they arrive without a face covering, a mask will be provided.

Revised Visit Scheduling Enhances Social Distancing:

- To support physical distancing and prevent congestion, intake appointment times will be arranged so that no more than 4 participants are present on site at any one time. There is ample space in Diefendorf to assure appropriate physical distancing with up to 8 private office spaces for participant interviews/counseling sessions.
- Clinic visits will be scheduled with at least 15 minutes staggering of arrivals and departures of other participants and clinic staff, and allocated duration of visits will be increased by 15 minutes to ensure time for disinfection of hard surfaces at the conclusion of each visit.

Consent Addendum: A consent addendum will be employed that advises participants of all COVID-related requirements and procedures. See Section 29.0: Process to Document Consent.

Disinfection of Shared Equipment and Spaces:

- Before and after each in-person appointment or use of a shared room or piece of equipment, all hard surfaces such as equipment (including the shared copier), countertops, keyboards, computer mice, office chair arms, and doorknobs will be disinfected with EPA-approved disinfectant wipes or spray.
- A disinfecting checklist will be placed on the door of each participant room or shared space; staff will provide the date, time, and staff initials after each disinfection.
- Participants will be given a pen to use during their visit that then will then take with them so multiple people aren't using the same pen.

Remote Study Visits:

Study visits will be conducted remotely, rather than in person at UB, under the following circumstances.

- If a participant reports COVID-19 symptoms or diagnosis, then remote visits will be scheduled at least until the participant is symptom-free and it has been at least 14 days since the date of diagnosis.
- If UB determines that research projects cannot have in-person visits for a period of time (for example, if there were a surge of COVID-19 cases in the area), all appointments will be conducted remotely during that period of time.
- Other circumstances in which study staff and the participant agree that one or more remote visits are appropriate in order to ensure uninterrupted smoking-cessation treatment
- To enhance compliance with follow-up appointments at which primary outcome measures are collected, these visits may be conducted remotely as well

Participants will be instructed regarding the details of remote study visits, including the need for privacy, the methods for delivery and return of study materials, and the technology (Zoom or telephone; REDCap) for completing study visits. Informed consent for remote study visits will be obtained with the aid of the attached *EVarQuit Consent Addendum – COVID-19.docx.*

UPDATE 2020-06-12 - ELIMINATION OF LAB VISITS FOR REMAINING PARTICIPANTS. As noted above, due to concerns about enrollment, participant burden, and perceived ppt risks, we are eliminating the lab visits for the remaining participants.

UPDATE 2020-06-30 – OPTIONAL SALIVA SAMPLE FOR GENETIC ANALYSIS. Consistent with the administrative supplement submitted to NCI, ITT participants will be asked to provide an optional additional saliva sample for genetic analysis. In brief, 30 minutes after eating, drinking, or smoking,

participants who consent to provide a genetics sample will provide a 2 mL saliva sample using an Oragene kit (DNA Genotek, Inc.).

For participants enrolled after approval of the genetics sample, an overview and the consent addendum (see EVarQuit Genetics Sub-Study - Consent Addendum - onsite - 2020-06-30.docx) will be presented at Clinic 2 (future participants). Reasons for waiting until Clinic 2 (as opposed to including with the initial study consent at intake) include: a) Because the major focus of these analyses is to better understand variability in response to varenicline, and varenicline is first measurable at Clinic 2, we do not wish to obtain samples from participants who are found ineligible or withdraw prior to Clinic 2, and b) Providing the consent addendum in close temporal proximity to obtaining the sample, rather than adding the information to the already lengthy consent form at intake, should enhance participant understanding of what is required versus optional and improve understanding of the specific issues related to the optional genetics sample.

For participants who have already completed Clinic 2, an overview and the consent addendum will be presented at their next study visit (see EVarQuit Genetics Sub-Study - Consent Addendum - onsite - 2020-06-30.docx). If the participant has already completed all required study visits, study staff will attempt to reach the participant by phone and/or email (see EVarQuit Genetics Sub-Study Initial Contact – Phone and Email Scripts 2020-06-30.docx). No more than two attempts with each method will be made, to avoid "hounding" the participant. If the participant is reached and expresses interest, or if the participant was not reached by phone/email, the following will be sent by postal mail:

- EVarQuit Genetics Sub-Study – Addendum Cover Letter – 2020-06-30.docx

- EVarQuit Genetics Sub-Study - Consent Addendum - home - 2020-06-30.docx

Participants who return written consent to providing the sample will be mailed standard DNA Genotek Oragene saliva sample kit and pre-paid return mailer. Participants will be remunerated upon receipt of the sample (see Remuneration).

Regarding issues relevant to Section 2 of HRP-399 (WORKSHEET: Additional Requirements for Genetic Testing (NY State)):

- Consent will be obtained directly from the participant (no samples taken from deceased individuals).
- As described in the consent addendum, samples will be stored independent of other participant information, making it impossible that genetic information would ever be incorporated into the records of a nonconsenting individual.
- Consent for banking and additional genetic testing are explicitly obtained in the consent addendum.
- As explicitly stated in the consent addendum: "If you say yes now, but you change your mind later, it will not be held against you or affect your participation in EVarQuit. You can always call (716-829-2323) or email us (EVarQuit@buffalo.edu) to say that you have changed your mind, and the DNA sample will be destroyed." In such an event, the PI (Dr. Hawk) will contact Dr. Tyndale at the University of Toronto to ensure the deidentified sample is destroyed.
- "Family members of an individual who provided a stored tissue sample will NOT be contacted for clinical, research, or other purposes without consent from the individual who provided the tissue sample with respect to the specific family members who will be contacted and the specific purpose of the contact." (HRP-399) As of this version of the protocol, we do not anticipate ever contacting family members of participants and would submit a modification in advance of any such contact.
- "Information about an individual derived from genetic tests performed on stored human tissue or information linking an individual with specific results of genetic tests will NOT be released to any organization or person without the explicit written consent of the individual who donated the stored tissue to release of the information for the purposes set forth in the written consent document" (HRP-399).

- "DNA samples will be stored for no more than ten years in the absence of genetic testing, if authorized in writing by the subject. If genetic testing will be performed on the stored samples or samples will be stored for more than 10 years, informed consent will be obtained" (HRP-399).

10.2 Describe what data will be collected.

NOTE: For studies with multiple data collection points or long-term follow up, consider the addition of a schedule or table in your response.

Response: Measures reflect the aims of the project: evaluating the efficacy of a promising approach to smoking cessation (Aim 1, with a focus on abstinence at EOT and 6M), and gaining insight into the mechanisms and moderators of treatment effects (Aims 2 and 3; with an emphasis on measures obtained between Intake and TQD). Please see the table of assessments and measures in Section 11.1

10.3 List any instruments or measurement tools used to collect data (e.g. questionnaire, interview guide, validated instrument, data collection form). Include copies of these documents with your submission.

Response:

Smoking rate – How many cigarettes did you smoke yesterday?

Expired-air CO – Biochemical verification obtained with a hand-held carbon monoxide (CO) meter (see CRF – e.g. EVQCRFs - Intake - Staff Instruments).

Cotinine/3-hydroxy-cotinine – Biochemical verification and rate of metabolism – patients will provide saliva samples at all Clinic visits as well as follow-up visits, (see App10.3-03).

Varenicline levels – patients will provide saliva samples at all Clinic visits, EOT and 6 month follow-up.

Validated Questionnaires that assess the following are included in App10.3-04- Questionnaires-2016-09-23.docx:

- Craving
- Withdrawal
- Subjective effects of smoking
- Nausea
- Treatment expectancies

The urine collection and drug testing procedure is described in App10.3-05-UrineToxProcedure.

Our standard side effects assessment (e.g., Hawk et al., 2012; Lerman et al., 2015) has been updated to include the CSSRS and is included on the CRFs for intake.

2020-04: App - COVID-19 Quitting Questions_v3.5 - EVarQuit 2020-04-20.docx

2020-05-19: App – COVID-19 Structured Interview-EVarQuit 2020-05-19.docx

10.4 Describe any source records that will be used to collect data about subjects (e.g. school records, electronic medical records).

Response: N/A, no external records will be used to collect data about subjects.

N/A. We will not obtain external source records.

10.5 Indicate whether or not ***individual*** *subject results, such as results of investigational diagnostic tests, genetic tests, or incidental findings will be shared with subjects or others (e.g., the subject's primary care physician) and if so, describe how these will be shared.*

Response: Individual subject results will not be shared with participants or others.

10.6 Indicate whether or not ***study*** *results will be shared with subjects or others, and if so, describe how these will be shared.*

Response: We will maintain a list of participants who would like to be notified of study results and will provide those participants with brief summaries of project results in short newsletters in the Fall of 2020, 2022, and 2024. These summaries will be shared via email or post based on participant preferences.

11.0 Study Timelines

11.1 Describe the anticipated duration needed to enroll all study subjects.

Response: 48 months

11.2 Describe the duration of an individual subject's participation in the study. Include length of study visits, and overall study follow-up time.

Response:

Participants will complete screening, active treatment, and long-term (6-month) follow-up, with an estimated total time commitment of ~~~26.5~~ 23 hours.

Phone screen	~ 0.5 hours
Intake	~ 2.0 hours
~~Lab Visit 1~~	~~~ 2.0 hours~~ Eliminated 2020-06-12
~~Lab Visit 2~~	~~~ 1.5 hours~~ Eliminated 2020-06-12
Clinic visits 1-6 @~1 hour each	~ 6.0 hours
Brief counseling check-ins 1-2	~ 0.5 hours
EMA @0.33 hours/day X @5 days/wk X 9 wks	~15.0 hours
5 1-minute follow-up surveys	~ 0.1 hours
EOT/6M/ follow-ups @ .5 hrs each	~ 1.0 hour

11.3 Describe the estimated duration for the investigators to complete this study (i.e. all data is collected and all analyses have been completed).

Response: 5.5 years (begin accrual in month 9; enroll last subject in month 51; complete 6-month follow-up in month 57; begin primary analyses)

12.0 Setting

12.1 Describe all facilities/sites where you will be conducting research procedures. Include a description of the security and privacy of the facilities (e.g. locked facility, limited access, privacy barriers). Facility, department, and type of room are relevant. Do not abbreviate facility names.

NOTE: Examples of acceptable response may be: "A classroom setting in the Department of Psychology equipped with a computer with relevant survey administration software," "The angiogram suite at Buffalo General Medical Center, a fully accredited tertiary care institution within New York State with badge access," or, "Community Center meeting hall."

Response:

The proposed clinical trial will take place in Dr. Hawk's lab on the third floor of Diefendorf Hall at the State University of New York at Buffalo. The third floor is secured with swipe card access and video-enabled two-way intercoms for enhanced security, privacy, and confidentiality. Within the lab are a range of individual rooms, each of which can be locked independently. Clinical assessments and cessation counseling are easily accommodated in the five interview rooms. Two medical exam rooms allow a range

of health-related assessments. A room dedicated to phlebotomy and urine toxicology is located adjacent to the lab restrooms; the room is equipped with a -5C freezer for short-term storage prior to transfer of samples to a 17-foot -80C freezer in the adjacent room for long-term storage. The research pharmacy has an on-site, alarmed room for storage of medications, reconciliation and randomization procedures, and relevant documentation. A white noise system enhance confidentiality between assessment rooms. A waiting room and kitchen with refreshments provide a welcome environment for participants, and a large seminar room provides ample space for study overview sessions.

Dr. Hawk and project staff will have access to approximately 15 PC computers data-entry, word processing, and clerical activities. All computers are on a network with centrally-maintained backups on a secure server that is accessible through Citrix software; the server is maintained by the Office of Medical Computing.

Laboratory assessments of reinforcement will take place in specialized research space dedicated to Dr. Hawk in Farber Hall (Rooms 155 and 157); backup smoking labs dedicated to Co-I Dr. Tiffany on the third floor of Park Hall may be used as a backup. This separation of laboratory assessments is by design; it separates the clinical smoking cessation and the lab assessments that involve smoking in a controlled environment. Drs. Hawk and Tiffany have offices on both campuses, allowing frequent interaction on the project. Swipe card (Hawk lab) and punch locks (Tiffany lab) separates the lab from hallway traffic, and each room within the lab is also secured with a standard door lock. Each lab consists of 500+ square feet of testing space, including two subject rooms and a master control room outfitted with equipment for complete CBUCC testing (test apparatus, computers, monitors, modified response boxes, keyboards, mouse for measuring response times with millisecond accuracy), refrigerators, high definition cameras in subject rooms, and secure access to the UB Box server that will maintain all study data. This test space is customized with ventilation and air handling systems that isolate the rooms from the rest of the building and allow for smoking in the test rooms with very high turnover air exchange ventilate directly to the exterior of the building.

12.2 For research conducted outside of UB and its affiliates, describe:

- *Site-specific regulations or customs affecting the research*
- *Local scientific and ethical review structure*

NOTE: This question is referring to UB affiliated research taking place outside UB, i.e. research conducted in the community, school-based research, international research, etc. It is not referring to multi-site research. UB affiliated institutions include Kaleida Health, ECMC, and Roswell Park Cancer Institute.

Response:

☒ **N/A:** This study is not conducted outside of UB or its affiliates.

13.0 Community-Based Participatory Research

13.1 Describe involvement of the community in the design and conduct of the research.

NOTE: Community-Based Participatory Research (CBPR) is a collaborative approach to research that equitably involves all partners in the research process and recognizes the unique strengths that each brings. CBPR begins with a research topic of importance to the community, has the aim of combining knowledge with action and achieving social change to improve health outcomes and eliminate health disparities.

Response:

☒ **N/A:** This study does not utilize CBPR.

13.2 Describe the composition and involvement of a community advisory board.

Response:

☒ **N/A:** This study does not have a community advisory board.

14.0 Resources and Qualifications

*14.1 Describe the qualifications (e.g., education, training, experience, expertise, or certifications) of the Principal Investigator **and** staff to perform the research. When applicable describe their knowledge of the local study sites, culture, and society. Provide enough information to convince the IRB that you have qualified staff for the proposed research.*

NOTE: If you specify a person by name, a change to that person will require prior approval by the IRB. If you specify a person by role (e.g., coordinator, research assistant, co-investigator, or pharmacist), a change to that person will not usually require prior approval by the IRB, provided that the person meets the qualifications described to fulfill their roles.

Response:

Co-PIs Hawk, Mahoney, and Tiffany will share leadership of the study, as described in the attached grant proposal (see Shared Leadership Plan in App00). This team has worked together on previous clinical trials, including the EvarQuit pilot study that led to the current trial (e.g., Hawk et al., 2012).

Dr. Hawk is a Professor of Psychology. His doctoral training in clinical health psychology provided him with an excellent background in theory, methods, and interventions for a range of health behaviors, and this expertise was enhanced by his post-doctoral fellowship in the Division of Behavioral Oncology at the University of Pittsburgh Cancer Institute. Over the past decade, he has developed expertise in smoking behavior and clinical cessation trials; he has conducted numerous smoking studies, including four randomized clinical trials (RCTs; two as Co-I, one as PI, one as site PI), all of which included pharmacotherapy and counseling. The two most recent trials (with Co-PI Mahoney) focused on an extinction-based model, using extended pre-quit pharmacotherapy to enhance smoking cessation; they provide the foundation for the proposed RCT. Dr. Hawk has provided and supervised delivery of cessation counseling and developed the treatment materials for the present study. Beyond RCTs, Dr. Hawk has published mechanism-oriented experimental work on the effects of nicotine, varenicline (the medication employed in the current proposal), and other drugs on basic reinforcement, cognitive, and subjective processes, as well as the role of these basic processes on the development of substance use. Overall, he is well-suited to serve as PI on the current proposal to evaluate the efficacy and mechanisms of extended pre-quit run-in varenicline for smoking cessation.

Dr. Mahoney is a Professor of Oncology and Staff Physician at Roswell Park Cancer Insitute (and has an appointment at UB). As PI or co-investigator, Dr. Mahoney has played key roles in the design, successful implementation and analyses of multiple smoking cessation clinical trials which have relied upon a variety of pharmacotherapies/interventions including: nicotine free cigarettes, St. John's Wort, bupropion, a nicotine conjugate vaccine, a nicotine liquid delivery system and varenicline. Together with Dr. Hawk, he recently participated in a multi-site cessation trial which used nicotine metabolism ratios (NMR) to randomize 1400+ smokers to either varenicline + placebo NRT, NRT + placebo varenicline or placebo.

Dr. Tiffany is an Empire Innovation Professor in Psychology. He brings considerable expertise derived from his ongoing research on the assessment of smoking and craving using ecological momentary assessment (EMA) technology (including work with Drs. Hawk and Mahoney; e.g., Gass et al., 2012; Hawk et al., 2012), processes of drug craving, the causes of drug dependence, the diagnosis of dependence, adolescent drug use, and the interaction of biological and psychological factors in the control of addictive behaviors. Dr. Tiffany's craving work focuses on understanding the role of drug craving in addiction. One of his longstanding interests is on the development and validation of instruments to sensitively and accurately measure drug craving; he has led development of widely used measures of alcohol, cigarette, cocaine, and heroin craving. Dr. Tiffany has also developed and validated multiple methods to study cue-specific craving and, of particular relevance to this research, have conducted research on the assessment of cue-reactivity in the natural environments of cigarette smokers. Dr. Tiffany was awarded the American Psychological Association Distinguished Scientific Award for Early Career Contribution to Psychology in 1993, and he has served as a member of several NIH scientific review panels.

Dr. Colder, who will handle the biostatistics and randomization for the current project, is a Professor of Psychology at UB. He has actively studied developmental models of psychopathology and adolescent

substance user for over 20 years. Dr. Colder has been Principal and Co-Investigator on multiple NIH funded longitudinal studies that span infancy to young adulthood. Dr. Colder's background also includes extensive training in quantitative methods, such as hierarchical linear models, structural equation modeling, growth modeling, mixture modeling, and testing moderation and mediation. Dr. Colder and Dr. Hawk have co-authored numerous publications from several collaborative studies at UB over the past 10 years.

Jennifer Adams, M.S.W., Research Coordinator, has worked for several years with Drs. Hawk and Mahoney on another large smoking cessation trial. Ms. Adams is familiar with the proposed assessments and procedures and will oversee all day-to-day aspects of the proposed study. She is already assisting with the development of the current IRB proposal and is familiar with UB IRB procedures, monitoring/reporting of side effects and adverse events. Ms. Adams, an M.S.W. with extensive experience in smoking cessation trials, will assist with implementation and training on psychiatric screening and cessation counseling. She will work with the research nurse to oversee sample collection and shipping, as per our standard protocols. Ms. Adams will work closely with Drs. Hawk and Colder to maximize retention at follow-up and interface between research pharmacist and project staff regarding medication disbursement and reconciliation. Ms. Adams will oversee recruitment, data collection and data entry to ensure all study activities are done according to GCP and within the appropriate timeframe. Ms. Adams will work with Drs. Hawk and other study investigators to refine all study protocols, respond to data management queries, review study charts to ensure the quality of the data captured.

Details for additional staff will be provided once the project has begun and we begin hiring.

Describe other resources available to conduct the research.

14.2 Describe the time and effort that the Principal Investigator and research staff will devote to conducting and completing the research.

NOTE: Examples include the percentage of Full Time Equivalents (FTE), hours per week. The question will elicit whether there are appropriate resources to conduct the research.

Response: Larry Hawk, Ph.D., Principal Investigator, (1.8 academic months and 1.8 summer months in all years). Dr. Hawk will be responsible for the scientific and technical direction of the proposed research. He will supervise most aspects of the project (see leadership plan), including hiring and training staff, supervising data collection, verification, and analysis, and leading manuscript and report preparation. Dr. Hawk will meet at least weekly with Research Coordinator and co-lead biweekly staff meetings and monthly calls with study investigators. Dr. Hawk will supervise the provision of behavioral counseling and lead the development of conference presentations and publications.

Stephen Tiffany, Ph.D., Principal Investigator, (1.2 academic months and 0.6 summer months in all years). Dr. Tiffany will take primary responsibility for the laboratory assessments of reinforcement during the pre-quit period. He will work closely with Dr. Hawk to coordinate clinical and laboratory assessments and Dr. Tiffany will assist in the management, reduction, and analysis of the laboratory data. Dr. Tiffany will also provide leadership and oversight on the ecological momentary Dr. Tiffany will contribute actively to work with all study investigators to interpret and disseminate results.

Craig Colder, Ph.D., Co-Investigator, (1.8 academic months and 0.6 summer months in YR01 and YR05; 0.9 academic months and 0.3 summer months in YR02-04). Dr. Colder will oversee the randomization procedures for the trial. Dr. Colder will also assist with tracking and maintaining retention during follow-up period, work with Dr. Hawk to coordinate integrated data management procedures, and lead data analysis as the project statistician. He will work with all study investigators to interpret and disseminate results.

Project Manager, TBN (1.2 calendar months in all years). The PM will consult with and assist PIs Hawk and Mahoney and Project Coordinator on high-level implementation and administration, as well as coordination of the proposed study with other projects in the CCF. As needed, she will lead intake visits and assist with staff training. She will also conduct protocol fidelity checks and provide an independent auditor of financial records, as required by institutional policy.

Jennifer Adams, MSW, Project Coordinator (12 calendar months in all years). The PC will assist the Co-PIs in submitting and maintaining IRB materials and monitoring/reporting side effects and adverse events.

The PC will oversee recruitment, data collection and data entry to ensure all study activities are done according to GCP and within the appropriate timeframe.

Nurse/Phlebotomist, TBN (3.6 calendar months in YR01, 4.8 calendar months in YR02-04, 2.4 calendar months in YR05). As in our recent multi-site cessation trial, the Nurse/Phlebotomist will assist the study MD and staff during the medical screening process. She will also conduct saliva samples and oversee urine toxicology and pregnancy screening at intake visits. She will assist with sample processing, storage, and shipping.

TBN, Research Support Specialists, (4@6.0 calendar months in YR01, 5@6 calendar months in YR02-04, 4@6.0 calendar months in YR05). The RSSs will aid in recruitment and retention efforts by conducting initial screenings, placing reminder phone calls, sending mail outs, and scheduling visits, under the supervision of the Project Coordinator. RSSs will be trained to conduct most study assessments per rigorous, detailed protocols, including the collection of lab reinforcement data and training participants in use of ecological momentary assessment. RSSs will work with the Coordinator to implement the daily operations of the study, including data entry, maintaining supply levels, and responding to data queries.

14.3 Describe the availability of medical or psychological resources that subjects might need as a result of anticipated consequences of the human research, if applicable.

NOTE: One example includes: on-call availability of a counselor or psychologist for a study that screens subjects for depression.

Response: Study staff will be available by phone during normal business hours, and they will have prompt access to Co-PIs Hawk (a clinical psychologist) and Mahoney (a physician) for clinical issues that arise in the course of smoking cessation with varenicline, as in our prior trials. As in our prior work, we will provide participants with contact information and reminder cards and, when appropriate, referrals for resources external to the focus of the project.

14.4 Describe your process to ensure that all persons assisting with the research are adequately informed about the protocol, the research procedures, and their duties and functions.

Response: All staff will be provided with copies of the grant proposal. Study protocols are provided in binders in the relevant rooms, and training of study staff will include direct observation of mock procedures, followed by supervision in real patient interactions. Duties will be documented in a continuously updated delegation log that will be signed by the staff member whenever there is a change.

15.0 Other Approvals

15.1 Describe any approvals that will be obtained prior to commencing the research (e.g., school, external site, funding agency, laboratory, radiation safety, or biosafety).

Response:

☒ **N/A:** This study does not require any other approvals.

16.0 Provisions to Protect the Privacy Interests of Subjects

16.1 Describe how you will protect subjects' privacy interests during the course of this research.

NOTE: Privacy refers to an individual's right to control access to him or herself. Privacy applies to the person. Confidentiality refers to how data collected about individuals for the research will be protected by the researcher from release. Confidentiality applies to the data.

Examples of appropriate responses include: "participant only meets with a study coordinator in a classroom setting where no one can overhear", or "the participant is reminded that they are free to refuse to answer any questions that they do not feel comfortable answering."

Response: Above (sections 9 and 12) we describe how we will protect subjects' privacy interests during the recruitment and consent phases. Throughout the research process, we respect participant's rights to refuse to complete any assessment and to withdraw from the study at any time, thus giving them control of

information access to themselves. (When appropriate, we will remind participants that refusal to complete assessments may lead the study investigators to withdraw the participant from the trial.)

Many of the measures will be self-administered, so participants will directly enter their responses into a computer/tablet/smartphone without the interviewer seeing their responses. This increases privacy and reduces potential discomfort.

Participants will meet individually with study investigators and staff in private offices; privacy is enhanced by the swipe card security system (limiting access) and the white noise system (reducing concerns about a conversation being overheard).

UPDATE 2020-05-29: COVID-19:

For remote visits during the COVID-19 pandemic, the use of telemedicine technology (Zoom; telephone calls), we will advise participants to attend the visit in a private setting. Zoom meetings will be password-protected, and a telephone (audio-only) option will be available.

UPDATE 2020-06-30 – OPTIONAL TROUBLESHOOTING SOFTWARE: As noted above, participants will have the option to download the TeamViewer app to assist with EMA app troubleshooting. The purpose of this software is to allow research staff to navigate to the EMA app and its settings to address problems more quickly and remotely so participants are less likely to miss assessments which could negatively impact their study eligibility and payment. The TeamViewer software will not be used for any other purposes beyond addressing problems with the EMA app. Staff will not access private information on the participant phone, such as photos, email, or texts messages, nor will they access functions such as the phone's camera. Participants have the option to decline having this app installed on their device. Additionally, the TeamViewer app requires the participant to actively consent to a troubleshooting session by clicking a pop-up to allow access each time research staff requests a remote access session. The participant can also end the remote session at any time.

16.2 Indicate how the research team is permitted to access any sources of information about the subjects.

*NOTE: Examples of appropriate responses include: school permission for review of records, consent of the subject, HIPAA waiver. This question **does apply** to records reviews.*

Response: Consent of the subject.

17.0 Data Management and Analysis

17.1 Describe the data analysis plan, including any statistical procedures. This section applies to both quantitative and qualitative analysis.

Response:

Our primary outcome of interest (Aims 1 and 3) is smoking cessation, a dichotomous variable indicating bio-verified (cotinine ≤15 ng/ml) self-report (TLFB) of continuous abstinence from smoking assessed at end-of-treatment (weeks 8-11 post-quit) and long-term follow-up (weeks 8-26 post-quit). We also propose to examine potential mechanisms of treatment effects, and our primary mediator of interest will be a continuous variable representing percent reduction in smoking behavior (CPD from daily EMA assessments) during the pre-quit phase of the study ([Week -5 CPD minus Week -1 CPD] / Week -5 CPD). Our proposed analyses and power estimates focus on these primary measures.

We focus above on percent reduction in CPD during the pre-quit period (from Week -5 to Week -1) because this measure is both feasible to assess in clinical practice and is emphasized in prior work on pre-quit pharmacotherapy (Hajek et al., 2011; Hawk et al., 2012, 2015; Rose et al., 1998). Indeed, some have suggested that achieving a 50% pre-quit reduction in smoking may be a clinically useful target (e.g., Rose & Behm, 2013), a hypothesis that could be evaluated in supplementary analyses.

In addition, our assessment strategy will allow us to examine the time course of changes during the pre-quit period in ways that may inform both theory and practice. This is true for reductions in CPD as well as the other proposed mediators. In prior work with small samples, extended run-in varenicline had, on average, a gradual impact on smoking and craving (Ashare et al., 2012; Hajek et al., 2011; Hawk et al., 2012; Poling et al. 2010). We will explore these trajectories at the group level but also consider individual differences in

change. For example, even among participants with comparable overall reductions in smoking, it is important to determine the degree to which abstinence is associated with a marked early decline in smoking (which may reflect a stronger blockade of reinforcement by pre-quit varenicline) or a more gradual reduction in smoking across the pre-quit period (which would allow more extinction “trials” to occur).

Consideration of additional measures of key constructs. Similarly, we plan to explore models of patterns of change across multiple pre-quit variables that are proposed mediators and their relation to smoking outcome. Of particular interest is whether we can identify a group characterized by a decline in smoking during the pre-quit period that is accompanied by declines in smoking satisfaction and/or craving. If the mechanism of the extended run-in operates as we hypothesize, this pattern should be more likely in the extended run-in than the standard run-in treatment group and be predictive of abstinence. Such analysis would involve growth mixture modeling to identify groups based on trajectories of smoking, smoking satisfaction, and craving during the pre-quit phase. Our team has extensive experience to extend our proposed analysis to growth modeling and growth mixture modeling (Colder et al., 2002, 2006, 2013, 2014; Trucco, Wright, & Colder, 2014).

In addition, we will have a rich data set to examine a variety of alternative outcomes and potential mediators and moderators; examples are provided below. An advantage of our study is that it includes multiple measures of smoking intake (CPD, CO, COT and 3HC) and additional measures relevant to reinforcement and extinction (subjective effects of smoking; laboratory-based indices of the relative reinforcement from cigarettes, food, and water), and measures of alternative (though not mutually incompatible) mechanisms of treatment effects such as craving, withdrawal, and nausea. This provides broad coverage of the variables that are both theoretically relevant and empirically supported as key processes in abstinence and relapse. Another advantage of our study is that we have assessed many of our variables using multiple methods. To maximize conceptual clarity and reduce the number of statistical tests, we will use confirmatory factor analysis, including Multitrait-Multimethod Measurement Models (MTMM, Kenny & Kashy, 1992) when appropriate, to inform construction of within-domain composites and/or selection of a subset of secondary measures for analysis. For each of the aims, it will also be important to consider a range of potential moderators and covariates, such as degree of nicotine dependence, NMR, age, education, and treatment outcome expectancies.

Primary analyses. Most of our analyses will be done in Mplus and SAS (Proc Mixed and Proc Glmmix), both of which are very flexible and can handle continuous, categorical, and non-normal data, and allow for the inclusion of cases with missing data.

Aim 1. We hypothesize that bio-verified continuous abstinence rates at end-of-treatment and at long-term follow-up will be greater in the extended run-in group compared to the standard run-in group. Logistic regression will be used to test this aim. Abstinence will be regressed on treatment group (a binary indicator). Given evidence that extended pre-quit varenicline will be particularly helpful for women (Hawk et al., 2012), we will include gender and the gender x treatment interaction to evaluate the hypothesis that gender moderates the impact of varenicline run-in duration.

Aim 2. We hypothesize that the extended run-in group will exhibit greater pre-quit reductions in smoking (percent reduction in CPD, as well as decreases in biochemical measures), as predicted by an extinction-of-reinforcement framework. Each hypothesized mediator will be regressed on treatment group (a binary indicator) using regression models appropriate for the nature of the mediator. As in Aim 1, gender and the gender x treatment group interaction will be included in the model. Comparable models will evaluate pre-quit changes in other candidate mediators (withdrawal, craving, subjective effects of smoking, nausea, and behavioral measures of smoking, food, and water reinforcement from the laboratory CBUCC paradigm). Together, these analyses provide critical information about the degree to which run-in group differences in smoking reduction reflect smoking-specific changes in reinforcement and related constructs, such as reinforcer devaluation (i.e., from nausea). In addition to their conceptual and theoretical significance, a clearer understanding of treatment mechanism may be important for predicting success prior to quitting, thereby allowing adjustments to treatment to prevent patients from experiencing a failed quit attempt (e.g., Rose et al., 2013) and providing precise targets to further enhance treatment effectiveness.

Aim 3. We hypothesize that changes in pre-quit smoking behavior (i.e., percent reduction in CPD during the pre-quit period) will mediate the effect of extended pre-quit varenicline on smoking cessation. This aim will be tested with a path model whereby treatment will predict pre-quit reduction in smoking (a continuous

variable), which in turn, will predict smoking abstinence. Separate models will be estimated for abstinence at EOT and 6M follow-up. Baseline levels of smoking will be included as a statistical control variable. Bootstrapped indirect effects with asymmetrical confidence bands will be used to test the proposed mediational path (MacKinnon 2008). We will also evaluate whether gender moderates this mediated path (moderated mediation, Preacher et al., 2007).

17.2 If applicable, provide a power analysis.

NOTE: This may not apply to certain types of studies, including chart/records reviews, survey studies, or observational studies. This question is asked to elicit whether the investigator has an adequate sample size to achieve the study objectives and justify a conclusion.

Response: Although our analyses will utilize methods to handle missing data (e.g., full-information likelihood estimation; Enders & Bandalos, 2001), we conservatively estimated power based on N=320 ITT participants and attrition estimated to be 5% at TQD, 18% at EOT, and 25% at 6-M (based on our varenicline data from Hawk et al., 2012 and Lerman et al., 2015). Our power estimates are based on an alpha level of .05 and adequate power considered ≥ .80. For the main effects of treatment run-in group on outcome (Aim 1) and hypothesized mediators (Aim 2), power was computed using Proc Power in SAS based on effect size estimates from Hawk et al. (2012) and Hajek et al. (2011); treatment run-in group effect sizes are expected to be in the range of small to medium for Aim 1 (Odds ratios 1.3 to 2.3) and Aim 2 (f2 .05 to .12). Our proposed sample will provide adequate power to detect these effects. For gender interactions in Aims 1 and 2 and all effects in Aim 3, we estimated power using Monte Carlo simulations estimated in Mplus (Muthén, & Muthén, 2002) with 10,000 replications and parameters taken from our pilot study (Hawk et al., 2012). Our monte carlo simulation suggested adequate power to detect the proposed gender x treatment interactions, with minimal bias in the regression coefficients and corresponding standard errors (bias < 3%). For Aim 3, our Monte Carlo simulation suggested adequate power to detect the proposed mediational pathway (collapsing across gender) with minimal bias in the estimated indirect effect and corresponding standard error (bias < 2%). Furthermore, our Monte Carlo simulation suggested adequate power to detect the gender x treatment interaction predicting the proposed mediator, and the proposed indirect effect for women with minimal bias for these effects and the corresponding standard errors (bias < 1%). Hence, we have adequate power to detect moderated mediation. Please note that our power calculations are unusually strong in that all effect size estimates were based on existing data, rather than hypothetical estimates, which we believe provides greater confidence in our calculations.

17.3 Describe any procedures that will be used for quality control of collected data.

Response: As part of the data and safety monitoring process, the team will ensure that all fields are completed appropriately, and all corrections are done according to Good Clinical Practice (GCP). Any inconsistencies/deviations will be documented. The Study Physician will review inclusion/exclusion data for each participant, documenting reviews of each report. The Project Manager will conduct quality control reviews of data on an on-going basis.

18.0 Confidentiality

A. Confidentiality of Study Data

Describe the local procedures for maintenance of confidentiality of ***study data and any records that will be reviewed for data collection****.*

18.1 A. Where and how will all data and records be stored? Include information about: password protection, encryption, physical controls, authorization of access, and separation of identifiers and data, as applicable. Include physical (e.g. paper) ***and*** *electronic files.*

Response:

Paper-based records, including source documents and an original consent form, will be maintained in locked filing cabinets in 306/307/308 Diefendorf Hall; keys are maintained in a safe in the office of the project-coordinator.

All participants will be assigned a numeric code. Electronic assessments and data management will be set up on secure web-based programs: https://ilumivu.com/solutions/ecological-momentary-assessment-app/ for daily EMA assessments and REDCap for electronic case report forms and overall project data management system. RedCap will temporarily be implemented through the University of Rochester CTSI. Due largely to the newness of our CTSA, UB does not have extensive RedCap support. UB CTSI COO Mary Sienkiewicz connected us with the U of Rochester CTSI (Carrie Irvine) for support. The websites are all HIPPA-compliant, have an SSL certificate (Secure Sockets Layer, a cryptographic protocol that provides communication security over the Internet), and use *https* (Hypertext Transfer Protocol Secure, a widely used communications protocol for secure communication over a computer network, with especially wide deployment on the Internet). No identifying information will be included in web-based electronic data files.

Local computer files (such as the recruitment database and a file linking identifying information with each participant's unique numeric code) will be maintained on a secure, password-protected UB server subject to regular backup. Files will be accessible only by study investigators and staff. The videos will be stored (labeled only with ID#) in a password protected database, behind an electronic firewall, and will only be accessed by the research team.

UPDATE 2020-05-29 – COVID-19:

For any remote visits, all assessments will be labeled with the numeric code representing the participant ID; they will not be labeled with the participant's name or other PII.

UPDATE 2020-06-30 – OPTIONAL TROUBLESHOOTING SOFTWARE: During remote troubleshooting sessions, research staff will document, for quality improvement purposes, basic smartphone information (e.g., model, operating system), the nature of the problem reported by the participant, steps taken to resolve the problem, and whether they were successful. No other information or data will be collected. According to the TeamViewer privacy policy, during remote access the software uses "…end-to-end encryption technology. This means that TeamViewer will not be aware of the content and subject matter of such exchanges." The TeamViewer software will collect and process information during remote access connection including "a Session ID, a meeting ID, and the start and end times of your session." No personal information is collected by the TeamViewer app during remote access sessions.

18.2 A. How long will the data be stored?

Response: Records containing identifying information will be stored for 3 years after completion of the project; they will then be destroyed.

As described in our grant proposal (App00), we will follow the NIH mandate for data-sharing. Our data-sharing plan is consistent with the 2015 Institute of Medicine (IOM) report, Sharing Clinical Trial Data: Maximizing Benefits, Minimizing Risk (National Academies Press); we plan to make the full de-identified analyzable data set with metadata available through the National Addiction and HIV Data Archive Program (NAHDAP) within 18 months of study completion. NHADAP is a NIDA-funded platform for data sharing. As recommended by NHADAP, we will work with NHADAP staff to begin the data-sharing plan prior to beginning data collection so that maximal study data are available to the public without compromising participant protections. NAHDAP has a standard data deposit form and required list of files (http://www.icpsr.umich.edu/icpsrweb/content/NAHDAP/deposit/index.html). The data deposit includes a standard procedure for ensuring participant protections (including NAHDAP recoding or dropping variables that might compromise confidentiality).

18.3 A. Who will have access to the data?

Response: Access to source documents and identifying information will be limited to project investigators and staff.

18.4 A. Who is responsible for receipt or transmission of the data?

Response: Study investigators and staff.

18.5 A. How will the data be transported?

Response: N/A Data will not be physically transported.

B. Confidentiality of Study Specimens

Describe the local procedures for maintenance of confidentiality of ***study specimens****.*

☐ **N/A:** No specimens will be collected or analyzed in this research. *(Skip to Section 19.0)*

18.6 B. Where and how will all specimens be stored? Include information about: physical controls, authorization of access, and labeling of specimens, as applicable.

Response: Urine samples will be used immediately for on-site drug and pregnancy screening and then discarded.

As in our recent trial, and saliva samples will be collected in Diefendorf 329 by the Nurse/Phlebotomist. Samples labeled with unique identifiers will be stored in Diefendorf 330 (which has both a key lock and an alarm), in a -80C freezer until batch shipped to the University of Toronto for analysis, as detailed in App00.

UPDATE 2020-06-30 –OPTIONAL SALIVA SAMPLE FOR GENETIC ANALYSIS:

As stated in the consent addendum: "To protect your confidentiality, the DNA sample will be stored without any identifying information, just an identification number. That number will be different from the code used to identify other data you provide in the EVarQuit program. An electronic master list linking your DNA identification number to your EVarQuit participation number will be password-protected and stored securely, separate from your other information."

As with our other saliva samples (for cotinine and varenicline concentrations), the saliva sample for genetic analysis will be assayed in the laboratory of Rachel Tyndale, Ph.D., at the University of Toronto. To protect participant confidentiality, Dr. Tyndale will not have access to any participant data except the uniquely coded samples.

Samples are obtained, labeled, transferred, stored, and shipped according to detailed protocols. see App10.3-03- Saliva_Collection_PNAT_120111-1_UB.pdf.

18.7 B. How long will the specimens be stored?

Response: All specimens will be destroyed after completion of assays described in the protocol/grant or within one year of completion of the project, whichever comes first.

UPDATE 2020-06-30 –OPTIONAL SALIVA SAMPLE FOR GENETIC ANALYSIS: Dr. Tyndale's lab will store the genetic samples for up to 10 years for future analysis for all participants who consent to banking.

18.8 B. Who will have access to the specimens?

Response: PIs, project coordinator, study nurse/phlebotomist, and any other staff member trained in sample acquisition or shipping.

18.9 B. Who is responsible for receipt or transmission of the specimens?

Response: PIs, project coordinator, study nurse/phlebotomist, and any other staff member trained in sample acquisition or shipping.

18.10 B. How will the specimens be transported?

Response: Specimens will be shipped via overnight on dry ice, as in our previous trials.

19.0 Provisions to Monitor the Data to Ensure the Safety of Subjects

☐ **N/A:** This study is not enrolling subjects, or is limited to records review procedures only. This section does not apply.

NOTE: Minimal risk studies may be required to monitor subject safety if the research procedures include procedures that present unique risks to subjects that require monitoring. Some examples include: exercising to exertion, or instruments that elicit suicidality or substance abuse behavior. In such cases, N/A is not an acceptable response.

19.1 Describe the plan to periodically evaluate the data collected regarding both harms and benefits to determine whether subjects remain safe.

Response: In this single-site trial, all participants receive varenicline (Chantix) for its approved use, smoking cessation.

Participant safety is a priority. The safety of individual participants will be assessed at each clinic visit, and aggregate data will be reviewed annually by the study team and by the IRB.

We will use a two-tiered system to assess potential side effects and adverse events. At each clinic visit, participants will complete an established checklist of symptoms and an open-ended evaluation for potential adverse events. Study staff will be trained to follow the procedures in App10-02 to trigger timely reporting of side effects and potential AEs to the Study Physician. Participants will also be given information (verbally and in a reminder wallet card) regarding how to contact the study personnel and under what circumstances to proceed to the emergency department. At any time, participants will have the option to stop taking the study medication and can drop out of the study if they desire. In addition, if any adverse event requires treatment and follow-up, participants will be provided with appropriate referrals. The Study Physician will determine the course of action for the subject reporting a serious adverse event (e.g., discontinuing medication, dose adjustment). The PI or Study Physician/Clinical Research Nurse will clinically follow all subjects who are discontinued due to a serious adverse event until the event is resolved.

In accordance with NIH and IRB guidelines, this study will employ the following mechanisms for adverse event reporting: 1) alert the site IRBs of any and all reports of serious adverse events; 2) inform all members of the study team of any and all reports of serious adverse events; and 3) notify NIH of any actions taken by IRBs with regard to data safety monitoring. Detailed procedures are formalized in App10-02.

Although we considered establishing a formal DSMB, this does not appear to be warranted for the current single-site trial that employs varenicline for its approved indication, smoking cessation. However, we do plan to summarize and review rates of side effects, adverse events, and efficacy data – all blind to treatment condition – as part of each renewal application to the IRB.

19.2 Describe what data are reviewed, including safety data, untoward events, and efficacy data.

Response: Standard assessments of side effects, detailed records of adverse events, and overall rates of smoking cessation.

19.3 Describe any safety endpoints.

Response: Standard assessments of side effects, detailed records of adverse events.

19.4 Describe how the safety information will be collected (e.g., with case report forms, at study visits, by telephone calls with participants).

Response: With case report forms at study visits.

19.5 Describe the frequency of safety data collection.

Response: At all 6 clinic visits, which occur at 1- to 2-week intervals in the first 2 months of treatment.

19.6 Describe who will review the safety data.

Response: As detailed in App10-02, study staff will review side effect reports at each study visit; standard decision rules are used to trigger reporting to the PI/Study Physician within 24 hours (often within minutes).

19.7 Describe the frequency or periodicity of review of cumulative safety data.

Response: Annually.

19.8 Describe the statistical tests for analyzing the safety data to determine whether harm is occurring.

Response: N/A. The base rates of serious adverse events are too small to be detected by statistical tests in the proposed sample size.

19.9 Describe any conditions that trigger an immediate suspension of the research.

Response: Given the nature of the trial, it is hard to foresee conditions that would lead to immediate suspension of the research. However, we would seek IRB input to consider immediate suspension if there were more than 2 SAEs (Codes 3 and 4 in App10-02) determined to be probably/definitely related to study participation during a single calendar month.

20.0 Withdrawal of Subjects

☐ **N/A:** This study is not enrolling subjects. This section does not apply.

20.1 Describe ***anticipated*** *circumstances under which subjects may be withdrawn from the research without their consent.*

Response: As described in the informed consent document:

Can I be removed from the research without my OK?

The principal investigator of the study can remove you from the research study without your approval. Possible reasons for removal include:

- The Principal Investigators feel it is necessary for your health or safety. Such an action would not require your consent, but you would be informed if such a decision was made and the reason for this decision.
- You have not followed program requirements.
- The Sponsor, University, or Investigators have decided to stop the program.

20.2 Describe any procedures for orderly termination.

NOTE: Examples may include return of study drug, exit interview with clinician. Include whether additional follow up is recommended for safety reasons for physical or emotional health.

Response: PI or designee will attempt to inform participants (by phone; if unable to contact, then by postal service) of the reason for withdrawal. No additional follow-up is necessary; however, in some situations it may be reasonable to provide alternative referral information, as discussed in other sections of the protocol.

20.3 Describe procedures that will be followed when subjects withdraw from the research, including retention of already collected data, and partial withdrawal from procedures with continued data collection, as applicable.

Response: As described in App10-02: "For all side effects that require attention, the site physician, qualified medical staff and PI will determine a course of action (i.e., continuation and monitoring, dose reduction, subject withdrawal). All side effects that are considered a Serious Adverse Event (see below) will be reported to PIs and IRBs …, as well as to the FDA and NIH (see below for protocol for adverse event reporting)… PIs and Study Physicians will determine if any serious adverse event requires additional care. Such events may be referred to the out-patient department … or to the emergency department … (…have access to 24-hour emergency services, including extensive in-patient and out-patient services for psychiatric conditions)."

UPDATE 2020-06-30 – OPTIONAL SALIVA SAMPLE FOR GENETIC ANALYSIS: As also noted in Procedures and explicitly stated in the consent addendum: "If you say yes now, but you change your

mind later, it will not be held against you or affect your participation in EVarQuit. You can always call (716-829-2323) or email us (EVarQuit@buffalo.edu) to say that you have changed your mind, and the DNA sample will be destroyed." In such an event, the PI (Dr. Hawk) will contact Dr. Tyndale at the University of Toronto to ensure the deidentified sample is destroyed.

21.0 Risks to Subjects

21.1 List the reasonably foreseeable risks, discomforts, hazards, or inconveniences to the subjects related to their participation in the research. Consider physical, psychological, social, legal, and economic risks. Include a description of the probability, magnitude, duration, and reversibility of the risks.

NOTE: Breach of confidentiality is always a risk for identifiable subject data.

Response: The potential risks to participants, and their likelihood and seriousness, are described below. Participants can choose, as an alternative, to not enroll in this study. Overall, there is minimal risk for serious adverse reactions as a consequence of enrolling in this study.

<u>Assessments</u>. Subjects may experience emotional distress during assessments from discussing feelings and attitudes about smoking or from learning about the risks from smoking. These events happen very rarely and in almost all cases are short-lived and of low intensity, lasting for 1-2 weeks. Study personnel will be alerted to expect this from a small number of subjects and will be trained to make referrals for mental health services as needed. Personnel will be trained to query for adverse emotional reactions during assessments and will be trained to deal with such reactions and to provide additional referrals if needed. In addition, if assessments indicate psychiatric concerns, referrals to appropriate psychological services will be provided.

<u>Withdrawal symptoms following cessation.</u> Most participants will experience some nicotine withdrawal upon quitting. Symptoms include craving, anxiety, irritability, problems concentrating, appetite change and weight gain, and insomnia. Because all subjects will use varenicline, withdrawal severity should be reduced. Moreover, withdrawal symptoms typically decrease markedly within 1-2 weeks. Study counseling will advise participants of these symptoms and discuss methods to cope with them.

<u>Varenicline</u>. In clinical trials, the most common side effects of Chantix include: nausea, sleep problems (trouble sleeping, changes in dreaming), constipation, gas, and vomiting. Chantix may also contribute to difficult sleeping, vivid, unusual, or strange dreams. Participants will be informed of the need to use caution driving or operating machinery until they are comfortable with how Chantix might affect them. Chantix should not be used with other quit-smoking products.

Some people have had reported changes in behavior, including hostility, agitation, depressed mood, suicidal thoughts or actions while using Chantix to help them quit smoking, with these symptoms developing when they began taking Chantix, and on occasion after several weeks of treatment or even after stopping Chantix. Participants will be counseled on these potential risks and encourage to contact us if they and/or their family/friends notice agitation, hostility, depression, or changes in behavior, thinking, or mood that are not typical, or if they develop suicidal thoughts or actions, anxiety, panic, aggression, anger, mania, abnormal sensations, hallucinations, paranoia, or confusion.

Varenicline also carries "warnings and precautions" regarding cardiovascular events, interactions with alcohol, seizures, and accidental injury. Varenicline may be associated with an increased risk of certain cardiac and vascular side effects, including chest pain, heart attack, and stroke. These risks are rare and are still being studied to determine how real they are. However, our study staff follows strict procedures to monitor for the presence of these side effects, including monitoring blood pressure at each in person visit and asking specific side effect questions related to cardiovascular events (e.g. chest pain, weakness on one side, etc) during each telephone session.

Because varenicline safety for an unborn baby is unknown, participants who are pregnant or nursing a baby, or planning to become pregnant, will be excluded from participation. All women of childbearing potential must agree to use an adequate form of contraception throughout the study and will be asked to take a pregnancy test at study intake. Women who become pregnant during the study will be removed from varenicline therapy but may still participate in counseling and study follow-up.

Threats to privacy/confidentiality. Since self-report and biological data will be collected and stored as part of this study, it is possible that subject privacy or confidentiality can be threatened.

UPDATE 2020-05-29 - COVID-19:

In the context of the pandemic, any interaction with another person or objects they have touched carries some risk of transmission of SARS-CoV-2, the virus that causes COVID-19.

In the case of remote visits, it is possible that subject privacy or confidentiality can be threatened.

21.2 Describe procedures performed to lessen the probability or magnitude of risks, including procedures being performed to monitor subjects for safety.

Response:

To further minimize the likelihood and severity of the aforementioned risks of varenicline:

1. We will employ select exclusionary criteria. For example, potential participants will be screened for current suicidal behavior and severe mood disorder and for very high levels of alcohol consumption.

2. We will administer the standard varenicline dose run-in and will not exceed the standard dose of 1 mg B.I.D.

3. We will discuss the potential risks of varenicline with prospective participants, including their likelihood. We will monitor self-reported side effects and Adverse Events at each of the clinic visits during the treatment period. Study Physician /PI will be alerted to side effects /Adverse Events, following our standardized protocol (see App10-02). The Study MD/PI will review the information provided by the research staff and if applicable, will contact the study participant directly to gather more information and determine the appropriate course of action for the subject. Ultimately, the Study Physician will decide if the AE is related to study medication and whether the subject should discontinue taking study medication.

To protect privacy and confidentiality, we have several safeguards against unauthorized access to study data – please see the sections of Privacy and Confidentiality sections of this document for details. We have not experienced the unauthorized use of study data.

Procedures for monitoring subjects for safety are presented in detail in Section 19.

UPDATE 2020-05-29: COVID-19:

Please see Section 11.1, above, for our extensive procedures to mitigate risk of transmission of SARS-CoV-2, the virus that causes COVID-19, as well as our monitoring for COVID-19 symptoms.

21.3 If applicable, indicate ***which procedures*** *may have risks to the subjects that are currently unforeseeable.*

Response: There may be risks that we do not know about at this time. We will notify participants of any new information that may affect their willingness to continue participation in this study.

21.4 If applicable, indicate which research procedures may have risks to an embryo or fetus should the subject be or become pregnant.

Response: Because varenicline safety for an unborn baby is unknown, participants will be told that they should not become pregnant while on this study. Women on the study should not nurse a baby. If the woman is of childbearing potential, she must use an adequate form of contraception while study medication is being taken and for at least one month after the end of the trial. If the woman is pregnant or breast feeding, she may not participate in this study, and if she becomes pregnant during the study, she will be removed from the study. Women will be asked to take a pregnancy test before starting the study.

21.5 If applicable, describe risks to others who are not subjects.

Response: We are not aware of any risks of this research to others who are not subjects.

22.0 Potential Benefits to Subjects

22.1 Describe the potential benefits that individual subjects may experience by taking part in the research. Include the probability, magnitude, and duration of the potential benefits. Indicate if there is no direct benefit.

*NOTE: Compensation **cannot** be stated as a benefit.*

Response: As described in the consent form –

"Will being in this study help me in any way?

We cannot promise any benefits to you or others from your taking part in this research. However, possible benefits include learning more about your smoking habit and quitting smoking. All participants receive free smoking cessation counseling and the most effective smoking cessation medication currently available (varenicline).

This clinical research study may show us how to make it easier for other smokers to quit smoking with varenicline in the future."

23.0 Compensation for Research-Related Injury

☐ **N/A:** The research procedures for this study do not present risk of research related injury (e.g. survey studies, records review studies). This section does not apply.

*23.1 **If the research procedures carry a risk of research related injury,** describe the available compensation to subjects in the event that such injury should occur.*

Response: As described in the consent form, under What else do I need to know?:

It is important that you tell your study doctor if you feel that you have been injured because of taking part in this study. You can tell the doctor in person or call. You will get medical treatment if you are injured as a result of taking part in this study. Your study doctor will explain the treatment options to you and tell you where you can get treatment. Generally, this care will be billed to you, your insurance or other third party. The University at Buffalo has no program to pay for medical care for research-related injury.

23.2 Provide a copy of contract language, if any, relevant to compensation for research related injury.

*NOTE: If the contract is not yet approved at the time of this submission, submit the current version here. If the contract is later approved with **different language regarding research related injury**, you must modify your response here and submit an amendment to the IRB for review and approval.*

Response: N/A – There is no contract other than the consent form.

24.0 Economic Burden to Subjects

24.1 Describe any costs that subjects may be responsible for because of participation in the research.

NOTE: Some examples include transportation or parking.

Response: Free parking will be made available, so that is not an issue.

As described in the consent form: "Neither you nor your insurance provider will be charged for costs of any of the procedures performed for the purpose of this research study (e.g., screening procedures, experimental procedures, medication, counseling, monitoring/follow-up procedures described above)."

☐ **N/A:** This study is not enrolling subjects, or is limited to records review procedures only. This section does not apply.

25.0 Compensation for Participation

25.1 Describe the amount and timing of any compensation to subjects, including monetary, course credit, or gift card compensation.

Response:

As detailed in the consent form, under What else do I need to know:

Prior to 2019-11 modification approval in early 2020-01:

If you agree to take part in this research study, we will pay you up to $599 for your time and effort. Although you will not receive any compensation for the initial health screening, we will pay you $15 for completing each of the 6 clinic visits in which you receive treatment. For each of the two lab visits, we will pay you up to $47. In return for completing electronic daily assessments on a phone or tablet, you can earn up to $315 over a 9-week period.

Because it is particularly important for us to know how you are doing after the treatment ends, we will pay you $15 for completing a brief phone call at 3 months after the planned quit date, and $15 for a 6- month call. During each of those 2 calls, we may ask you to come back to UB to provide a saliva sample and complete a few additional measures, for which you would receive an additional $35 each time.

Beginning with 2019-11 modification submission (implemented early 2020-01):

If you agree to take part in this research study, we will pay you up to $598 for your time and effort. Although you will not receive any compensation for the initial health screening, we will pay you for your time and effort in completing other project requirements. For each of the two 2-hour lab visits, we will pay you up to $54. In return for completing electronic daily assessments on a phone or tablet, you can earn up to $315 over a 9-week period ($1 per completion for each of the 35 assessments per week).

Because it is particularly important for us to know how you are doing over time, we will pay you up to $25 for completing a 1-minute computerized survey sent to your phone at 6, 8, 10, 18, and 22 weeks after your Target Quit Date ($5 for each of the 5 surveys). You may also be asked to come back to Diefendorf Hall to provide breath and saliva samples and complete a few surveys at 11 and 26 weeks after your Target Quit Date; you would receive $75 for each of those visits.

UPDATE 2020-06, to reflect elimination of lab visits:

If you agree to take part in this research study, we will pay you up to $490 for your time and effort. Although you will not receive any compensation for the initial health screening, we will pay you for your time and effort in completing other project requirements. In return for completing electronic daily assessments on a phone or tablet, you can earn up to $315 over a 9-week period ($1 per completion for each of the 35 assessments per week).

Because it is particularly important for us to know how you are doing over time, we will pay you up to $25 for completing a 1-minute computerized survey sent to your phone at 6, 8, 10, 18, and 22 weeks after your Target Quit Date ($5 for each of the 5 surveys). You may also be asked to come back to Diefendorf Hall to provide breath and saliva samples and complete a few surveys at 11 and 26 weeks after your Target Quit Date; you would receive $75 for each of those visits.

Payments will be made with a reimbursable Mastercard (ClinCard), typically at the end of each visit. Although we will not require you to complete tax forms, federal law requires that you report all income to the Internal Revenue Service (IRS).

UPDATE 2020-06-30 – OPTIONAL SALIVA SAMPLE FOR GENETIC ANALYSIS: As stated in the consent addendum, participants who can provide the optional sample on site will receive $25. Participants who complete and mail the sample from home will receive a higher amount of remuneration, $50, because of the additional requirements for collection, preparing the package to mail, and mailing the package.

☐ **N/A:** This study is not enrolling subjects, or is limited to records review procedures only. This section does not apply.

☐ **N/A:** There is no compensation for participation. This section does not apply.

26.0 Consent Process

26.1 Indicate whether you will be obtaining consent.

NOTE: This does not refer to consent documentation, but rather whether you will be obtaining permission from subjects to participate in a research study.
Consent documentation is addressed in Section 27.0.

☒ ***Yes*** *(If yes, Provide responses to each question in this Section)*
☐ ***No*** *(If no, Skip to Section 27.0)*

26.2 Describe where the consent process will take place. Include steps to maximize subjects' privacy.

Response: Consenting will take place in Diefendorf Hall, Rooms 307/308. An individual overview will be followed by consenting of individual participants.

***UPDATE 2020-06-30 – OPTIONAL SALIVA SAMPLE FOR GENETIC ANALYSIS*:** Participants will be consented individually either in a private interview room on the third floor of Diefendorf Hall or, if all study visits are complete, will have the consent addendum mailed to them for their review at home; questions will be addressed in person, by email, and/or by phone, as needed and preferred by the participant.

26.3 Describe how you will ensure that subjects are provided with a sufficient period of time to consider taking part in the research study.

NOTE: It is always a requirement that a prospective subject is given sufficient time to have their questions answered and consider their participation. See "SOP: Informed Consent Process for Research (HRP-090)" Sections 5.5 and 5.6.

Response: After the overview, participants will be invited to take as much time as they like to read the consent form and to ask any questions that they may have. Participants may also take the protocol home with them to review and/or discuss with family, physician, etc.; in this case, the participant would contact us to schedule their intake visit, where we would complete the consent process. Data collection will not begin until the participant has agreed to participate and signed the consent form.

26.4 Describe any process to ensure ongoing consent, defined as a subject's willingness to continue participation for the duration of the research study.

Response: Although we will obtain written consent only once in this relatively short term study (each participant is active in the study for ~1 year), participants who raise concerns about continuing participation will always be reminded that they are free to withdraw from the study at any time.

26.5 Indicate whether you will be following "SOP: Informed Consent Process for Research (HRP-090)." If not, or if there are any exceptions or additional details to what is covered in the SOP, describe:

- *The role of the individuals listed in the application who are involved in the consent process*
- *The time that will be devoted to the consent discussion*
- *Steps that will be taken to minimize the possibility of coercion or undue influence*
- *Steps that will be taken to ensure the subjects' understanding*

Response: Yes, we will follow SOP HRP-090. In particular, we draw attention to sections 4, 5.1, 5.4, 5.5, 5.6, 5.7, 5.8, 5.9, 5.10.3, and 6.1, 6.5.

☒ We have reviewed and will be following "SOP: Informed Consent Process for Research (HRP-090)."

Non-English Speaking Subjects

☒ **N/A:** This study will not enroll Non-English speaking subjects.
(Skip to Section 26.8)

26.6 Indicate which language(s) other than English are likely to be spoken/understood by your prospective study population or their legally authorized representatives.

NOTE: The response to this Section should correspond with your response to Section 6.4 of this protocol.

Response:

26.7 If subjects who do not speak English will be enrolled, describe the process to ensure that the oral and written information provided to those subjects will be in that language. Indicate the language that will be used by those obtaining consent.

NOTE: Guidance is provided on "SOP: Informed Consent Process for Research (HRP-090)."

Response:

Cognitively Impaired Adults

☒ **N/A**: This study will not enroll cognitively impaired adults.
(Skip to Section 26.9)

26.8 Describe the process to determine whether an individual is capable of consent.

Response:

Adults Unable to Consent

☒ **N/A**: This study will not enroll adults unable to consent.
(*Skip to Section 26.13)*

When a person is not capable of consent due to cognitive impairment, a legally authorized representative should be used to provide consent (Sections 26.9 and 26.10) ***and, where possible, assent of the individual should also be solicited*** *(Sections 26.11 and 26.12).*

26.9 Describe how you will identify a Legally Authorized Representative (LAR). Indicate that you have reviewed the "SOP: Legally Authorized Representatives, Children, and Guardians (HRP-013)" for research in New York State.

NOTE: Examples of acceptable response includes: verifying the electronic medical record to determine if an LAR is recorded.

Response:

☐ We have reviewed and will be following "SOP: Legally Authorized Representatives, Children, and Guardians (HRP-013)."

26.10 ***For research conducted outside of New York State****, provide information that describes which individuals are authorized under applicable law to consent on behalf of a prospective subject to their participation in the research. One method of obtaining this information is to have a legal counsel or authority review your protocol along with the definition of "legally authorized representative" in "SOP: Legally Authorized Representatives, Children, and Guardians (HRP-013)."*

Response:

26.11 Describe the process for ***assent of the adults****:*

- *Indicate whether assent will be obtained from all, some, or none of the subjects. If some, indicate which adults will be required to assent and which will not.*

Response:

- *If assent will not be obtained from some or all subjects, provide an explanation of why not.*

Response:

*26.12 Describe whether **assent of the adult** subjects will be documented and the process to document assent.*

NOTE: The IRB allows the person obtaining assent to document assent on the consent document using the "Template Consent Document (HRP-502)" Signature Block for Assent of Adults who are Legally Unable to Consent.

Response:

Subjects who are not yet Adults (Infants, Children, and Teenagers)

☒ **N/A**: This study will not enroll subjects who are not yet adults.
(Skip to Section 27.0)

*26.13 Describe the criteria that will be used to determine **whether a prospective subject has not attained the legal age for consent to treatments or procedures involved in the research** under the applicable law of the jurisdiction in which the research will be conducted **(e.g., individuals under the age of 18 years)**. For research conducted in NYS, review "SOP: Legally Authorized Representatives, Children, and Guardians (HRP-013)" to be aware of which individuals in the state meet the definition of "children."*

NOTE: Examples of acceptable responses include: verification via electronic medical record, driver's license or state-issued ID, screening questionnaire.

Response:

*26.14 **For research conducted outside of New York State**, provide information that describes which persons have not attained the legal age for consent to treatments or procedures involved the research, under the applicable law of the jurisdiction in which research will be conducted. One method of obtaining this information is to have a legal counsel or authority review your protocol along the definition of "children" in "SOP: Legally Authorized Representatives, Children, and Guardians (HRP-013)."*

Response:

26.15 Describe whether parental permission will be obtained from:

Response:

☐ One parent even if the other parent is alive, known, competent, reasonably available, and shares legal responsibility for the care and custody of the child.

☐ Both parents unless one parent is deceased, unknown, incompetent, or not reasonably available, or when only one parent has legal responsibility for the care and custody of the child.

☐ Parent permission will not be obtained. A waiver of parent permission is being requested.

NOTE: The requirement for parent permission is a protocol-specific determination made by the IRB based on the risk level of the research. For guidance, review the "CHECKLIST: Children (HRP-416)."

*26.16 Describe whether permission will be obtained from individuals **other than parents**, and if so, who will be allowed to provide permission. Describe your procedure for determining an individual's authority to consent to the child's general medical care.*

Response:

*26.17 Indicate whether assent will be obtained from all, some, or none of the **children**. If assent will be obtained from some children, indicate which children will be required to assent.*

Response:

26.18 When assent of children is obtained, describe how it will be documented.

Response:

27.0 Waiver or Alteration of Consent Process

Consent will not be obtained, required information will not be disclosed, or the research involves deception.

☒ **N/A:** A waiver or alteration of consent is not being requested.

27.1 If the research involves a waiver or alteration of the consent process, please review the "CHECKLIST: Waiver or Alteration of Consent Process (HRP-410)" to ensure that you have provided sufficient information for the IRB to make the determination that a waiver or alteration can be granted.

NOTE: For records review studies, the first set of criteria on the "CHECKLIST: Waiver or Alteration of Consent Process (HRP-410)" applies.

Response:

27.2 If the research involves a waiver of the consent process for planned emergency research, please review the "CHECKLIST: Waiver of Consent for Emergency Research (HRP-419)" to ensure you have provided sufficient information for the IRB to make these determinations. Provide any additional information necessary here:

Response:

28.0 Process to Document Consent

☐ **N/A:** A Waiver of Consent is being requested.
(Skip to Section 29.0)

28.1 Indicate whether you will be following "SOP: Written Documentation of Consent (HRP-091)." If not or if there are any exceptions, describe whether and how consent of the subject will be obtained including whether or not it will be documented in writing.

NOTE: If your research presents no more than minimal risk of harm to subjects and involves no procedures for which written documentation of consent is normally required outside of the research context, the IRB will generally waive the requirement to obtain written documentation of consent. This is sometimes referred to as 'verbal consent.' Review "CHECKLIST: Waiver of Written Documentation of Consent (HRP-411)" to ensure that you have provided sufficient information.

1901 1902 *If you will document consent in writing, attach a consent document with your submission. You may use "TEMPLATE CONSENT DOCUMENT (HRP-502)". If you will obtain consent, but not document consent in writing, attach the script of the information to be provided orally or in writing (i.e. consent script or Information Sheet).*

Response:

Informed consent is App28-InformedConsentWithHIPPA—EvarQuit-2016-09-26

☒ We will be following "SOP: Written Documentation of Consent" (HRP-091).

Update 2020-5-21 – Further assessing COVID-19 impact: Verbal consent will be obtained for newly implemented procedures for this survey only.

Update 2020-5-29 - COVID-19-related procedural changes: A consent addendum (EVarQuit Consent Addendum – COVID-19.docx) to be read, discussed, and signed at the Intake Visit, describes all COVID-related requirements and procedures.

UPDATE 2020-06-30 – OPTIONAL SALIVA SAMPLE FOR GENETIC ANALYSIS: As described in Procedures, an optional consent addendum (EVarQuit Genetics Sub-Study - Consent Addendum - onsite - 2020-06-30.docx OR EVarQuit Genetics Sub-Study - Consent Addendum - home - 2020-06-30.docx) will be read, discussed, and signed at Clinic 2 or later (and always prior to obtaining the optional saliva sample for genetic analysis). The consent addendum addresses the requirements of Section 4 of HRP-399 – Additional Requirements for Genetic Testing (NY State) and the procedure for withdrawal of consent and destruction of samples (see Section 2 of HRP-399).

29.0 Multi-Site Research (Multisite/Multicenter Only)

☒ **N/A:** This study is not an investigator-initiated multi-site study. This section does not apply.

29.1 If this is a multi-site study ***where you are the lead investigator****, describe the processes to ensure communication among sites, such as:*

- *All sites have the most current version of the IRB documents, including the protocol, consent document, and HIPAA authorization.*
- *All required approvals have been obtained at each site (including approval by the site's IRB of record).*
- *All modifications have been communicated to sites, and approved (including approval by the site's IRB of record) before the modification is implemented.*
- *All engaged participating sites will safeguard data as required by local information security policies.*
- *All local site investigators conduct the study appropriately.*
- *All non-compliance with the study protocol or applicable requirements will be reported in accordance with local policy.*

Response:

29.2 Describe the method for communicating to engaged participating sites:

- *Problems*
- *Interim results*
- *Study closure*

Response:

29.3 Indicate the total number of subjects that will be enrolled or records that will be reviewed across all sites.

Response:

29.4 If this is a multicenter study for which UB will serve as the IRB of record, and subjects will be recruited by methods not under the control of the local site (e.g., call centers, national advertisements) describe those methods.

Response:

30.0 Banking Data or Specimens for Future Use

☐ **N/A:** This study is not banking data or specimens for future use or research outside the scope of the present protocol. This section does not apply.

30.1 If data or specimens will be banked (stored) for ***future use, that is, use or research outside of the scope of the present protocol****, describe where the data/specimens will be stored, how long they will be stored, how the data/specimens will be accessed, and who will have access to the data/specimens.*

NOTE: Your response here must be consistent with your response at the "What happens if I say yes, I want to be in this research?" Section of the Template Consent Document (HRP-502).

Response:

UPDATE 2020-06-30 – OPTIONAL SALIVA SAMPLE FOR GENETIC ANALYSIS: With participant consent (see consent addendum), the DNA saliva sample will be banked in the lab of Dr. Rachel Tyndale at the University of Toronto (Room 4326, 1 King's College Circle, University of Toronto, Toronto, Ontario M5S 1A8, Canada) for up to 10 years for additional genetic analysis. Dr. Tyndale is a Professor of Pharmacology and Toxicology and the Head of Pharmacogenomics at the Centre for Addiction and Mental Health. As noted above, the sample will be stored without any additional information. No one will have access to the genetic data/specimens without permission of Dr. Tyndale, with prior approval from EVarQuit PIs Hawk/Mahoney.

30.2 List the data to be stored or associated with each specimen.

Response:

UPDATE 2020-06-30 – OPTIONAL SALIVA SAMPLE FOR GENETIC ANALYSIS: None. No data will be stored or associated with each sample.

30.3 Describe the procedures to release banked data or specimens for future uses, including: the process to request a release, approvals required for release, who can obtain data or specimens, and the data to be provided with specimens.

Response:

UPDATE 2020-06-30 – OPTIONAL SALIVA SAMPLE FOR GENETIC ANALYSIS: No banked sample will be released/analyzed without prior IRB approval. As noted in 31.2, no data are stored with each sample.

31.0 Drugs or Devices

☐ **N/A:** This study does not involve drugs or devices. This section does not apply.

31.1 If the research involves drugs or devices, list and describe all drugs and devices used in the research, the purpose of their use, and their regulatory approval status.

Response: All participants will receive varenicline (Chantix) for its FDA-approved indication, smoking cessation.

31.2 Describe your plans to store, handle, and administer those drugs or devices so that they will be used only on subjects and be used only by authorized investigators.

Response: Pfizer will provide all study medication for the trial. As in our previous trials, the UB Research Pharmacy (in the school of Pharmacy and Pharmaceutical Sciences) will receive all study medication from Pfizer and will oversee medication packaging, dispensing, accountability logs at study visits, and eventual destruction of unused medication. The Pharmacy has an alarmed room on site (Diefendorf Hall, Room 330) for on-site storage (in a locked cabinet within the alarmed room) and documentation.

If the drug is investigational (has an IND) or the device has an IDE or a claim of abbreviated IDE (non-significant risk device), include the following information:

31.3 Identify the holder of the IND/IDE/Abbreviated IDE.

Response: N/A – Study medication is not investigational.

31.4 Explain procedures followed to comply with FDA sponsor requirements for the following:

	Applicable to:		
FDA Regulation	***IND Studies***	***IDE studies***	***Abbreviated IDE studies***
21 CFR 11	***X***	***X***	
21 CFR 54	***X***	***X***	
21 CFR 210	***X***		
21 CFR 211	***X***		
21 CFR 312	***X***		
21 CFR 812		***X***	***X***
21 CFR 820		***X***	

Response: N/A – Study medication is not investigational.

32.0 Humanitarian Use Devices

XX **N/A:** This study does not involve humanitarian use devices. This does not apply.

32.1 For Humanitarian Use Device (HUD) uses provide a description of the device, a summary of how you propose to use the device, including a description of any screening procedures, the HUD procedure, and any patient follow-up visits, tests or procedures.

Response:

32.2 For HUD uses provide a description of how the patient will be informed of the potential risks and benefits of the HUD and any procedures associated with its use.

Response:
