# Supplementary material for: Effect of Extending the Duration of Prequit Treatment With Varenicline on Smoking Abstinence: A Randomized Clinical Trial
Source: JAMA Netw Open. 2022 Nov 11;5(11):e2241731. doi: 10.1001/jamanetworkopen.2022.41731 (PMC9652761; doi:10.1001/jamanetworkopen.2022.41731)
Supplement: Supplement 2. — eAppendix 1. Supplemental Information on Categorical Participant Characteristics eAppendix 2. Supplemental Abstinence Analyses eAppendix 3. Supplemental Analyses of Reduction in Smoking Exposure Across the Prequit Period eAppendix 4. Craving and Withdrawal eAppendix 5. Pill Count Medication Adherence eAppendix 6. Symptom Report Data [file jamanetwopen-e2241731-s002.pdf]

Supplementary Online Content

Hawk LW Jr, Tiffany ST, Colder CR, et al. Effect of extending the duration of prequit treatment with varenicline on smoking abstinence: a randomized clinical trial. *JAMA Netw Open*. 2022;5(11):e2241731. doi:10.1001/jamanetworkopen.2022.41731

eAppendix 1. Supplemental Information on Categorical Participant Characteristics
eAppendix 2. Supplemental Abstinence Analyses
eAppendix 3. Supplemental Analyses of Reduction in Smoking Exposure Across the Prequit Period
eAppendix 4. Craving and Withdrawal
eAppendix 5. Pill Count Medication Adherence
eAppendix 6. Symptom Report Data

This supplementary material has been provided by the authors to give readers additional information about their work.

© 2022 Hawk LW Jr et al. *JAMA Network Open*.

eAppendix 1. Supplemental Information on Categorical Participant Characteristics

eTable A1. Detailed Presentation of Categorical Baseline Participant Characteristics

**Gender**<br>**Run-In Group**	**Male**<br>**Standard**<br>**(n = 68)**	**Male**<br>**Extended**<br>**(n = 73)**	**Female**<br>**Standard**<br>**(n = 89)**	**Female**<br>**Extended**<br>**(n = 90**	**Total**<br>**Standard**<br>**(n = 157)**	**Total**<br>**Extended**<br>**(n = 163)**
Race, n (%)						
White / Caucasian	55 (80.9%)	59 (80.8%)	63 (70.8%)	63 (70.8%)	118 (75.2%)	122 (75.3%)
Black / African American	10 (14.7%)	9 (12.3%)	24 (27.0%)	24 (27.0%)	34 (21.7%)	33 (20.4%)
American Indian / Alaska Native	1 (1.5%)	1 (1.4%)	1 (1.1%)	0 (0.0%)	2 (1.3%)	1 (0.6%)
Other	2 (2.9%)	2 (2.7%)	1 (1.1%)	2 (2.2%)	3 (1.9%)	4 (2.5%)
More than 1 race	0 (0.0%)	2 (2.7%)	0 (0.0%)	0 (0.0%)	0 (0.0%)	2 (1.2%)
Total	68 (100%)	73 (100%)	89 (100%)	89 (100%)	157 (100%)	162 (100%)
Education, n (%)						
9th or 10th grade	0 (0.0%)	1 (1.4%)	1 (1.1%)	0 (0.0%)	1 (0.7%)	1 (0.6%)
11th or 12th grade, no diploma	2 (3.1%)	2 (2.9%)	2 (2.3%)	3 (3.4%)	4 (2.6%)	5 (3.2%)
High school graduate	9 (13.8%)	11 (15.7%)	12 (13.8%)	15 (17.0%)	21 (13.8%)	26 (16.5%)
GED or equivalent	7 (10.8%)	9 (12.9%)	5 (5.7%)	2 (2.3%)	12 (7.9%)	11 (7.0%)
Some college, no degree	23 (35.4%)	23 (32.9%)	17 (19.5%)	24 (27.3%)	40 (26.3%)	47 (29.7%)
Associate degree	15 (23.1%)	13 (18.6%)	28 (32.2%)	18 (20.5%)	43 (28.3%)	31 (19.6%)
Bachelors degree (example: BA, BS)	9 (13.8%)	9 (12.9%)	13 (14.9%)	21 (23.9%)	22 (14.5%)	30 (19.0%)
Masters degree (example: MA, MS)	0 (0.0%)	1 (1.4%)	7 (8.0%)	5 (5.7%)	7 (4.6%)	6 (3.8%)
Doctoral degree (example: PhD, EdD)	0 (0.0%)	1 (1.4%)	0 (0.0%)	0 (0.0%)	2 (1.3%)	0 (0.0%)
Professional degree (example: MD, DDS)	0 (0.0%)	0 (0.0%)	2 (2.3%)	0 (0.0%)	0 (0.0%)	1 (0.6%)
Total	65 (100%)	70 (100%)	87 (100%)	88 (100%)	152 (100%)	158 (100%)
Employment, n (%)						
Working full time	34 (52.3%)	33 (48.5%)	37 (42.5%)	38 (43.2%)	71 (46.7%)	71 (45.5%)
Working part-time	4 (6.2%)	6 (8.8%)	8 (9.2%)	13 (14.8%)	12 (7.9%)	19 (12.2%)
Only temporarily laid off, sick	2 (3.1%)	2 (2.9%)	1 (1.1%)	1 (1.1%)	3 (2.0%)	3 (1.9%)
Looking for work, unemployed	4 (6.2%)	1 (1.5%)	5 (5.7%)	2 (2.3%)	9 (5.9%)	3 (1.9%)
Retired	11 (16.9%)	19 (27.9%)	15 (17.2%)	16 (18.2%)	26 (17.1%)	35 (22.4%)
Disabled, permanently or temporarily	10 (15.4%)	7 (10.3%)	18 (20.7%)	14 (15.9%)	28 (18.4%)	21 (13.5%)
Homemaker	0 (0.0%)	0 (0.0%)	2 (2.3%)	2 (2.3%)	2 (1.3%)	2 (1.3%)
Student	0 (0.0%)	0 (0.0%)	1 (1.1%)	1 (1.1%)	1 (0.7%)	1 (0.6%)
Other	0 (0.0%)	0 (0.0%)	0 (0.0%)	1 (1.1%)	0 (0.0%)	1 (0.6%)
Total	65 (100%)	68 (100%)	87 (100%)	88 (100%)	152 (100%)	156 (100%)

© 2022 Hawk LW Jr et al. *JAMA Network Open*.

eTable A1. Detailed Presentation of Categorical Baseline Participant Characteristics
(Continued)

Gender<br>Run-In Group	Male<br>Standard<br>(n = 68)	Male<br>Extended<br>(n = 73)	Female<br>Standard<br>(n = 89)	Female<br>Extended<br>(n = 90	Total<br>Standard<br>(n = 157)	Total<br>Extended<br>(n = 163)
Income, n (%)						
Less than $15,000	2 (3.3%)	2 (3.0%)	8 (9.6%)	7 (8.6%)	10 (6.9%)	9 (6.1%)
$15,000-$24,999	8 (13.1%)	10 (15.2%)	9 (10.8%)	7 (8.6%)	17 (11.8%)	17 (11.6%)
$25,000-$34,999	6 (9.8%)	5 (7.6%)	7 (8.4%)	11 (13.6%)	13 (9.0%)	16 (10.9%)
$35,000-$49,999	6 (9.8%)	9 (13.6%)	16 (19.3%)	16 (19.8%)	22 (15.3%)	25 (17.0%)
$50,000-$74,999	16 (26.2%)	18 (27.3%)	20 (24.1%)	16 (19.8%)	36 (25.0%)	34 (23.1%)
$75,000-$99,999	12 (19.7%)	12 (18.2%)	15 (18.1%)	13 (16.0%)	27 (18.8%)	25 (17.0%)
$100,000-$149,999	8 (13.1%)	5 (7.6%)	6 (7.2%)	9 (11.1%)	14 (9.7%)	14 (9.5%)
$150,000-$199,999	2 (3.3%)	3 (4.5%)	2 (2.4%)	1 (1.2%)	4 (2.8%)	4 (2.7%)
More than $200,000	1 (1.6%)	2 (3.0%)	0 (0.0%)	1 (1.2%)	1 (0.7%)	3 (2.0%)
Total	61 (100%)	66 (100%)	83 (100%)	81 (100%)	144 (100%)	147 (100%)
Relationship Status, n (%)						
Married	27 (42.2%)	34 (47.9%)	36 (41.4%)	35 (40.2%)	63 (41.7%)	69 (43.7%)
Widow/Widower	3 (4.7%)	4 (5.6%)	10 (11.5%)	7 (8.0%)	13 (8.6%)	11 (7.0%)
Divorced	13 (20.3%)	13 (18.3%)	16 (18.4%)	15 (17.2%)	29 (19.2%)	28 (17.7%)
Separated	0 (0.0%)	2 (2.8%)	4 (4.6%)	2 (2.3%)	4 (2.6%)	4 (2.5%)
Single, Never married	8 (12.5%)	7 (9.9%)	9 (10.3%)	18 (20.7%)	17 (11.3%)	25 (15.8%)
Living with a partner	10 (15.6%)	7 (9.9%)	11 (12.6%)	10 (11.5%)	21 (13.9%)	17 (10.8%)
Other	3 (4.7%)	4 (5.6%)	1 (1.1%)	0 (0.0%)	4 (2.6%)	4 (2.5%)
Total	64 (100%)	71 (100%)	87 (100%)	87 (100%)	151 (100%)	158 (100%)
Longest Quit, n (%)						
Never	4 (6.2%)	2 (2.8%)	3 (3.4%)	5 (5.7%)	7 (4.6%)	7 (4.4%)
< 1 week	7 (10.8%)	12 (16.9%)	14 (16.1%)	7 (8.0%)	21 (13.8%)	19 (11.9%)
1-2 weeks	7 (10.8%)	11 (15.5%)	8 (9.2%)	10 (11.4%)	15 (9.9%)	21 (13.2%)
3-4 weeks	6 (9.2%)	5 (7.0%)	6 (6.9%)	8 (9.1%)	12 (7.9%)	13 (8.2%)
5-8 weeks	4 (6.2%)	6 (8.5%)	5 (5.7%)	5 (5.7%)	9 (5.9%)	11 (6.9%)
3-6 months	11 (16.9%)	8 (11.3%)	12 (13.8%)	7 (8.0%)	23 (15.1%)	15 (9.4%)
7-11 months	7 (10.8%)	10 (14.1%)	11 (12.6%)	17 (19.3%)	18 (11.8%)	27 (17.0%)
1-2 years	6 (9.2%)	10 (14.1%)	11 (12.6%)	16 (18.2%)	17 (11.2%)	26 (16.4%)
3-4 years	7 (10.8%)	1 (1.4%)	9 (10.3%)	3 (3.4%)	16 (10.5%)	4 (2.5%)
5 years+	6 (9.2%)	6 (8.5%)	8 (9.2%)	10 (11.4%)	14 (9.2%)	16 (10.1%)
Total	65 (100%)	71 (100%)	87 (100%)	88 (100%)	152 (100%)	159 (100%)

© 2022 Hawk LW Jr et al. *JAMA Network Open*.

eAppendix 2. Supplemental Abstinence Analyses

Supplemental outcomes: 7-day point prevalence (7DPP) abstinence. In addition to continuous abstinence, we evaluated 7DPP abstinence at Week 6, 8, 15 (EOT [End of Treatment[), and 28 (6M [6 months]), with abstinence operationalized as no self-reported smoking in the past 7 days and cotinine of ≤15 ng/mL (see Supplemental Figure B1). Neither the run-in treatment group effect nor the Gender x Group interaction was significant at any point. Though there was no evidence of a gender difference in 7DPP abstinence at Week 6 or 8, 7DPP was modestly lower among women compared to men at Weeks 15 and 28. Post-hoc tests of the run-in group effect among women only were non-significant at all time points, for C5 (Clinic 5), C6, EOT, and 6M, respectively (see Table B1 for descriptive and inferential statistics).

Supplemental covariate-adjusted analyses. To reduce the number of potential covariates to those that accounted for unique variance in our primary abstinence outcome, continuous abstinence at EOT, we conducted a series of logistic regressions in Mplus (Muthén & Muthén, 1998-2017), using MLR (Robust Maximum Likelihood) estimation to account for missingness. The initial logistic regression model included the following baseline characteristics: self-reported age, education, marital status, race, employment, income, average CPD (Cigarettes Per Day), FTCD (Fagerstrom Test of Cigarette Dependence), age began smoking daily, years smoked daily, menthol vs. non-menthol cigarettes smoked, presence of others who smoke in the home, and average cigarette pack cost, number of previous quit attempts, and longest previous quit duration, as well as weight, expired-air CO (carbon monoxide), COT (cotinine), NMR (Nicotine Metabolite Ratio), and COVID-19 context (whether any visit window occurred on or after March 20, 2020, the date on which the COVID-19 shutdown began in New York state). A backward elimination approach was taken in which the covariate with the largest non-significant *p* value was eliminated in each successive model until only significant predictors of abstinence remained. The final covariate model included only marital status (married or living with a partner vs. all other categories; the former was associated with greater odds of abstinence) and cotinine (the higher the baseline cotinine concentration, the lower odds of abstinence), ORs = 0.57 [.35,.92] and 1.24 [1.05,1.47],*P*s = .02 and .01, respectively. Next, gender, group, and the Gender x Group interaction were added to the final covariate-adjusted model for continuous abstinence at EOT. The results were comparable to the primary analysis without covariates: group and Gender x Group were non-significant, *p*s = .17 and .16, ORs = 1.6 [.83,2.92] and 0.51 [.20,1.3], and there was a trend for higher rates of abstinence among men, *p*=.08, OR = 1.81 [.92,3.54]. Supplemental covariate-adjusted models for continuous abstinence at 6M and for 7DPP abstinence at C5, C6, EOT, and 6M all failed to yield significant group, gender, or Gender x Group effects, all *P*s>.09.

Supplemental covariate-adjusted moderator analyses. Finally, we extended the covariate-adjusted model in Mplus to evaluate a small set of candidate moderators of the run-in treatment group effect: age, FTCD, NMR, menthol vs. non-menthol cigarettes, self-reported race (only black vs. white was included due to small cell sizes in other racial categories), and participation during COVID-19, as operationalized above. In each model, the candidate moderator and its' interaction with run-in group were added to the base model that always included: marital status, cotinine, gender, group, and Gender x Group. The Group x Moderator interaction was of primary interest; because Gender x Group x Moderator cell sizes would have been relatively small, the 3-way interaction term was not included. As shown in Supplemental Tables B2 and B3, none of the 6 candidate moderators entered into an interaction with Run-In group for any of the 6 abstinence outcomes we examined, all *P*s>.06.

Supplemental multiple imputation analyses. Multiple imputation was used as an alternative to coding participants with missing data (self-report and/or cotinine) as smoking for the continuous abstinence outcomes (EOT, 6M). The prevalence of missing data was 20.9% (n = 67) at EOT and 23.4% (n = 75) at 6M. The imputation model included our theoretical variables of interest (treatment group, gender, and their interaction) and additional auxiliary variables associated with missingness (age, years smoked daily, Clinic 1 cotinine, Clinic 1 NMR, post-Covid-shutdown enrollment). Imputation was performed using SPSS28 and separate imputation models were run for EOT and 6M. Twenty imputations were specified using the iterative Markov chain Monte Carlo method (FCS: Fully Conditional Specification Method). Candidate imputed values were constrained to their observed range. The relative efficiency statistic was used to evaluate the result of the imputation analyses with larger values close to 1.0 being desired. For both analyses (EOT and 6M), the relative efficiency values were > .98 for the theoretical variables. When we re-ran our models to test study hypotheses with imputed data, results did not change any of our conclusions. This suggests that our findings were robust to how missing data were handled.

© 2022 Hawk LW Jr et al. *JAMA Network Open*.

eTable A2. *P* Values, Odds Ratios (ORs), and 95% CIs for Bioverified 7-Day Point Prevalence Abstinence at Treatment Weeks 6, 8, 15, and 28.

	**Week 6 (Clinic 5)**		**Week 8 (Clinic 6)**		**Week 15 (EOT: End of Treatment)**		**Week 28 (6M)**	
**Effect**	***p* value**	**OR (95%CI)**	***p* value**	**OR (95%CI)**	***p* value**	**OR (95%CI)**	***p* value**	**OR (95%CI)**
Treatment Group	.62	1.13 (0.70,1.81)	.35	1.24 (0.79,1.93)	.54	1.15 (0.73,1.80)	.34	1.28 (0.77,2.14)
Gender	.98	1.00 (0.62,1.60)	.56	1.14 (0.73,1.79)	.08	1.50 (0.96,2.35)	.18	1.42 (0.85,2.36)
Group x Gender	.19	0.53 (0.20,1.38)	.26	0.60 (0.24,1.46)	.19	0.54 (0.22,1.34)	.19	0.51 (0.18,1.42)
Treatment Group for Males only	.52	0.79 (0.39,1.61)	.83	0.93 (0.48,1.81)	.58	0.83 (0.43,1.61)	.79	0.91 (0.44,1.88)
Treatment Group for Females only	.22	1.49 (0.79,2.83)	.15	1.56 (0.86,2.84)	.18	1.52 (0.82,2.81)	.11	1.79 (0.87,3.71)

eTable A3. *P* Values, Odds Ratios (ORs), and 95% 95% CIs for Run-In Group × Candidate Moderator and Gender × Run-In Group × Candidate Moderator Interaction Tests, Presented for Bioverified Continuous Abstinence at End of Treatment (EOT) and 6-Month Follow-up (6M).

**Candidate Moderator**	**EOT**		**6M**	
	***p* value**	**OR (95%CI)**	***p* value**	**OR (95%CI)**
Group x Moderator				
Age	0.55	0.99 (0.94,1.03)	0.96	1.00 (0.95,1.05)
FTCD	0.38	1.11 (0.88,1.41)	0.30	1.17 (0.87,1.58)
NMR	0.63	0.77 (0.27,2.23)	0.07	0.37 (0.13,1.06)
Smokes menthol	0.43	1.48 (0.56,3.87)	0.13	0.41(0.13,1.32)
Race: B vs W	0.84	0.89 (0.29,2.76)	0.80	0.84 (0.22,3.16)
COVID context	0.86	1.09 (0.43,2.78)	0.37	1.67 (0.55,5.12)
Gender x Group x Moderator				
Age	0.64	1.02 (0.93,1.13)	0.90	1.01 (0.90,1.12)
FTCD	0.62	0.89 (0.55,1.43)	0.62	0.86 (0.48,1.56)
NMR	0.27	0.24 (0.02,3.04)	0.80	0.76 (0.06,10.17)
Smokes menthol	0.95	0.94 (0.13,6.61)	0.44	2.56 (0.22,29.39)
Race: B vs W	0.10	7.90 (0.68,91.76)	0.40	3.49 (0.19,63.32)
COVID context	0.23	3.15 (0.48,20.66)	0.35	2.93 (0.31,28.13)

Notes. B=Black; FTCD=Fagerstrom Test of Cigarette Dependence; NMR=Nicotine Metabolite Ratio; W=White. Given that most participants self-identified as black or white, race was analyzed as Black vs. White, with other race categories and combinations excluded.

© 2022 Hawk LW Jr et al. *JAMA Network Open*.

eTable A4. *P* Values, Odds Ratios (ORs), and 95% 95% CIs for Run-In Group × Candidate Moderator and Gender × Run-In Group × Candidate Moderator Interaction Tests, Presented for Bioverified 7-Day Point Prevalence at Clinic 5 (C5), Clinic 6 (C6), End of Treatment (EOT), and 6-Month Follow-up (6M)

**Candidate Moderator**	**C5**		**C6**		**EOT**		**6M**	
	p value	OR(95%CI)	*p* value	OR(95%CI)	*p* value	OR(95%CI)	*p* value	OR(95%CI)
Group x Moderator								
Age	0.18	1.04(0.98,1.10)	0.88	1.00(0.96,1.05)	0.88	1.00(0.95,1.04)	0.96	1.00(0.95,1.05)
FTCD	0.26	1.15(0.90,1.48)	0.73	1.04(0.82,1.32)	0.23	1.16(0.91,1.46)	0.22	1.19(0.90,1.57)
NMR	0.47	1.50(0.50,4.48)	0.49	0.67(0.22,2.05)	0.82	1.14(0.37,3.48)	0.61	0.74(0.24,2.31)
Smokes menthol	0.75	0.85(0.30,2.39)	0.74	0.85(0.32,2.25)	0.46	1.43(0.55,3.72)	0.19	0.48(0.16,1.43)
Race: B vs W	0.74	1.23(0.36,4.19)	0.39	1.64(0.53,5.04)	0.86	0.91(0.30,2.73)	0.87	0.90(0.27,3.05)
COVID context	0.42	0.66(0.25,1.80)	0.10	0.45(0.18,1.16)	0.89	1.07(0.42,2.71)	0.85	1.11(0.39,3.20)
Gender x Group x Moderator								
Age	0.27	1.07(0.95,1.19)	0.79	0.99(0.89,1.09)	0.71	1.02(0.93,1.12)	0.67	0.98(0.88,1.09)
FTCD	0.27	0.76(0.46,1.24)	0.24	0.75(0.47,1.21)	0.66	0.90(0.56,1.45)	0.65	0.88(0.51,1.52)
NMRC1	0.38	0.32(0.03,4.05)	0.61	1.93(0.16,23.95)	0.56	0.43(0.03,7.29)	0.84	1.32(0.09,19.30)
Smokes menthol	0.89	0.86(0.10,7.15)	0.51	1.94(0.27,14.03)	0.55	0.55(0.08,3.82)	0.18	4.52(0.50,41.36)
Race: B vs W	0.98	0.96(0.08,11.62)	0.14	5.96(0.57,61.93)	0.09	7.86(0.71,87.14)	0.51	2.41(0.18,32.41)
COVID context	0.05	7.42(0.98,56.45)	0.07	5.92(0.88,40.03)	0.11	4.60(0.71,29.94)	0.09	6.49(0.75,56.48)

Notes. B=Black; FTCD=Fagerstrom Test of Cigarette Dependence; NMR=Nicotine Metabolite Ratio; W=White. Given that most participants self-identified as black or white, race was analyzed as Black vs. White, with other race categories and combinations excluded.

© 2022 Hawk LW Jr et al. *JAMA Network Open*.

eFigure A1. Bioverified 7-Day Point Prevalence (7DPP) Abstinence Rates at Treatment Weeks 6, 8, 15, and 28 (2, 4, 11, and 24 Weeks After Target Quit Date [TQD]), Presented for all Run-In Group × Gender Conditions

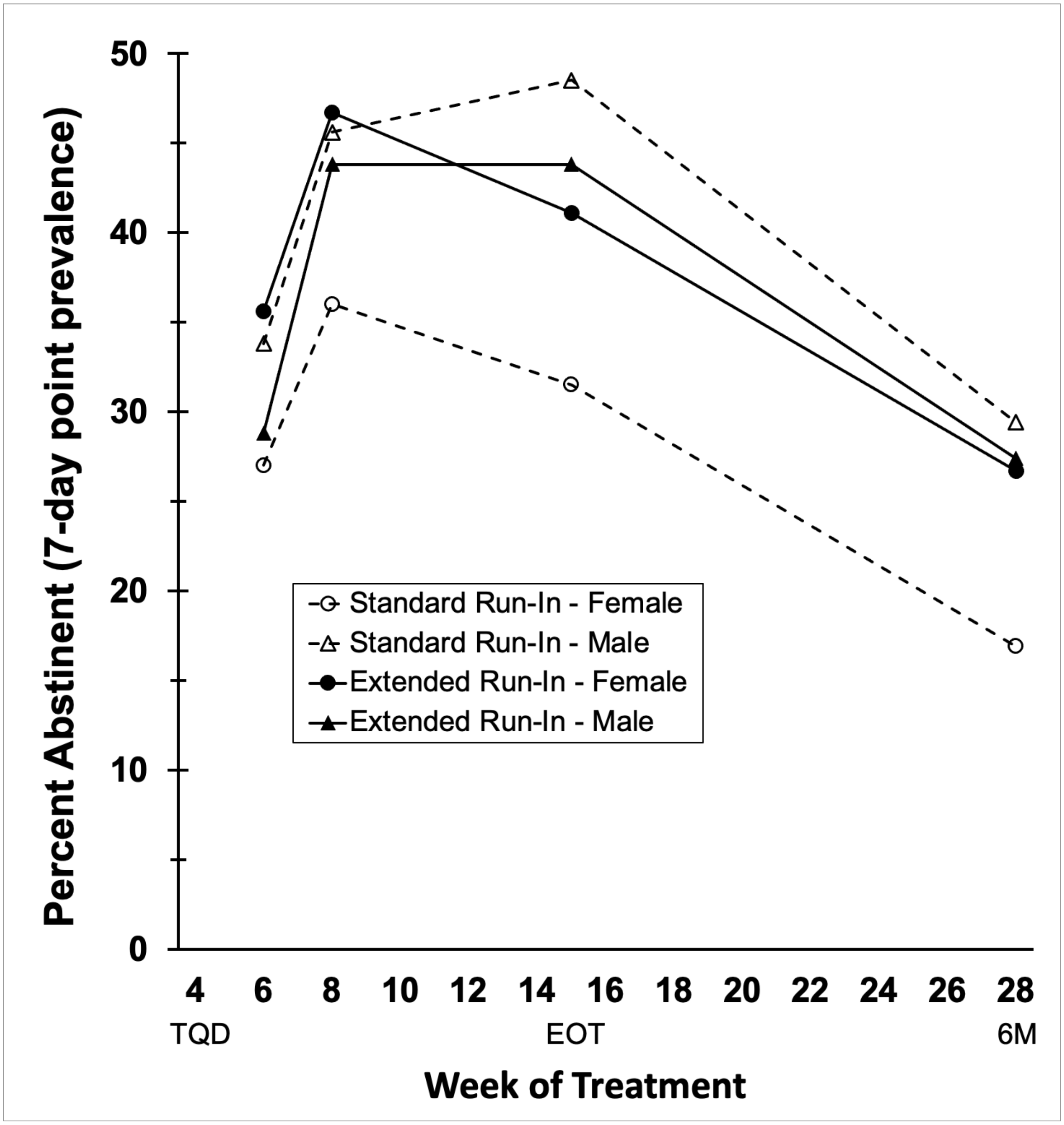

© 2022 Hawk LW Jr et al. *JAMA Network Open*.

eAppendix 3. Supplemental Analyses of Reduction in Smoking Exposure Across the Prequit Period

Percent reduction in cotinine during the pre-quit period ([Week 0 – Week 4]/Week 1 x 100) was, on average, greater among the extended (mean=54.1%; 95%CI=48.7%-59.5%) compared to the standard run-in group (mean=33.5%, 95%CI=28.2%-38.9%), group *P*<.001. Gender was unrelated to percent pre-TQD cotinine reduction, *P*s>.51.

Supplemental analysis of pre-TQD changes in smoking exposure across weeks employed multi-level models to accommodate the nested data structure. Treatment week was examined with linear and quadratic contrasts, with Week 4 (which ended on TQD) as the intercept. Significant interactions involving week were followed-up with models respecifying the intercept at each week.

As shown in Supplemental Figure C1, self-reported smoking rate (cigarettes per day [CPD] from time-line follow-back (TLFB) was comparable for the Extended and Standard Run-In Groups during the initial week of treatment, *P*=.80. Smoking rate declined across weeks, linear and quadratic *P*s<.001 and .01, respectively. However, the rate of decline was somewhat stronger in the Extended Run-In Group, Group x Week quadratic *P*=.07, with significantly lower CPD reported by the Extended Run-In Group compared to the Standard Run-In group during the latter half of the pre-quit period, *P*s=.25, .05, and .02, for Treatment Weeks 2, 3, and 4, respectively. Self-reported smoking rate was greater among men compared to women, at intercept (week 4) *P*<.01; all interactions of gender with week and/or run-in condition *P*s>.51.

Similarly, decline in cotinine across the pre-TQD period was greater among the extended compared to the standard run-in group (see Supplemental Figure C2), Group × Week linear and quadratic, *P*s=.04 and <.001. The groups were equivalent at baseline, Week 0, *P*=.87, but differed at Weeks 1, 3, and 4, *P*s<.02, .001, and .001, respectively. Pre-TQD changes in salivary cotinine were unrelated to gender, all *P*s>.23.

© 2022 Hawk LW Jr et al. *JAMA Network Open*.

eFigure A2. Changes in Self-Reported Smoking Rate Across the Prequit Period, Presented for All Run-In Group × Gender Conditions

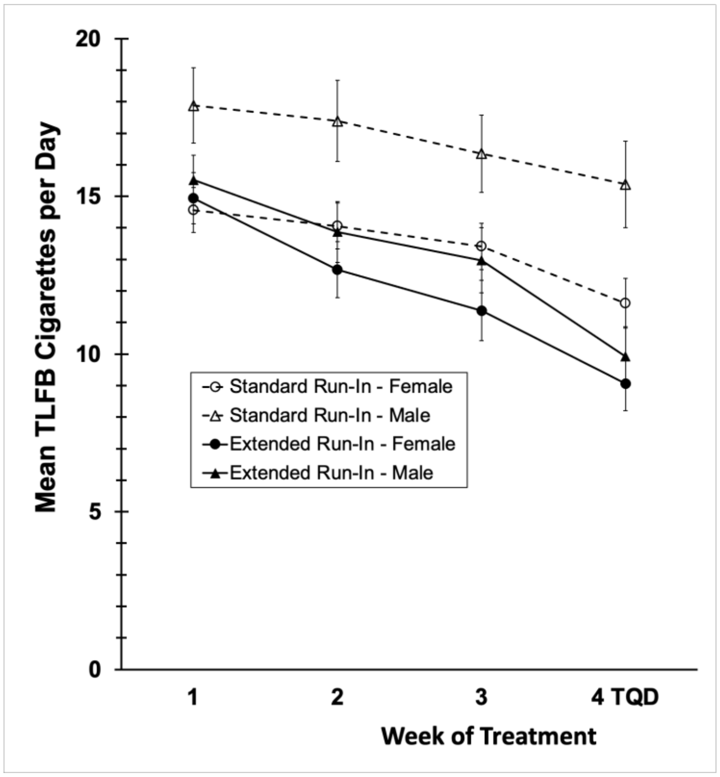

Error bars are +/-1 standard error.

© 2022 Hawk LW Jr et al. *JAMA Network Open*.

eFigure A3. Changes in Cotinine Across the Prequit Period, Presented for all Run-In Group × Gender Conditions

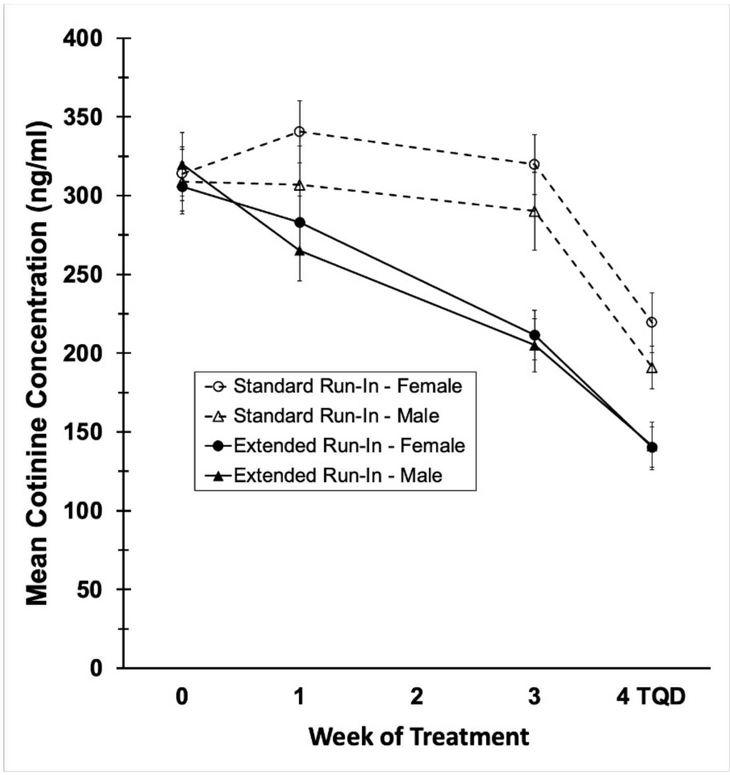

Error bars are +/-1 standard error.

© 2022 Hawk LW Jr et al. *JAMA Network Open*.

eAppendix 4. Craving and Withdrawal

Self-reported craving and withdrawal were analyzed via multi-level models to accommodate the nested data structure. Treatment week was examined with linear and quadratic contrasts, with Week 4 (which ended on TQD) as the intercept. Significant interactions involving week were followed-up with models respecifying the intercept at each week. Piece-wise growth models evaluated craving and withdrawal in the pre-TQD (Weeks 0-4) and post-quit (Weeks 4-8) periods.

On average, craving decreased from baseline through the first 3 weeks of treatment, increased at TQD (Target Quit Date), Weeks 0-4 linear and quadratic, *P*s<.001, and then decreased further across the first month post-TQD (see Supplemental Figure D1, Panel A), Weeks 4-8 linear and quadratic, *P*s<.001. At Week 4/TQD, craving was greater in the Standard compared to the Extended Run-In Group, *P*<.001, a difference that was stronger among women than men, *P*=.015. The group difference in craving was also evident at Week 3, *P*=.003, but not at other weeks, all *P*s >.07, Group x Weeks 0-4 linear *P*=.03; Group x Weeks 4-8 linear *P*<.001.

As can be seen in Supplemental Figure D1 (Panel B), withdrawal was mild and did not significantly change from Week 0 to Week 4/TQD, *P*s>.18. On average, withdrawal at Week 4/TQD was greater among women than men, *P*=.036, but did not vary between run-in groups, *P*s>.95.

In the 4 weeks following TQD, withdrawal tended to increase in the Standard Run-In Group but decline in the Extended Run-In group, a pattern that tended to be stronger among women, Gender x Group x Weeks 4-8 linear and quadratic, *P*s=.073 and .067.

eFigure A4. Time Course of Craving (A) and Smoking Withdrawal (B), Presented for all Run-In Group × Gender Conditions

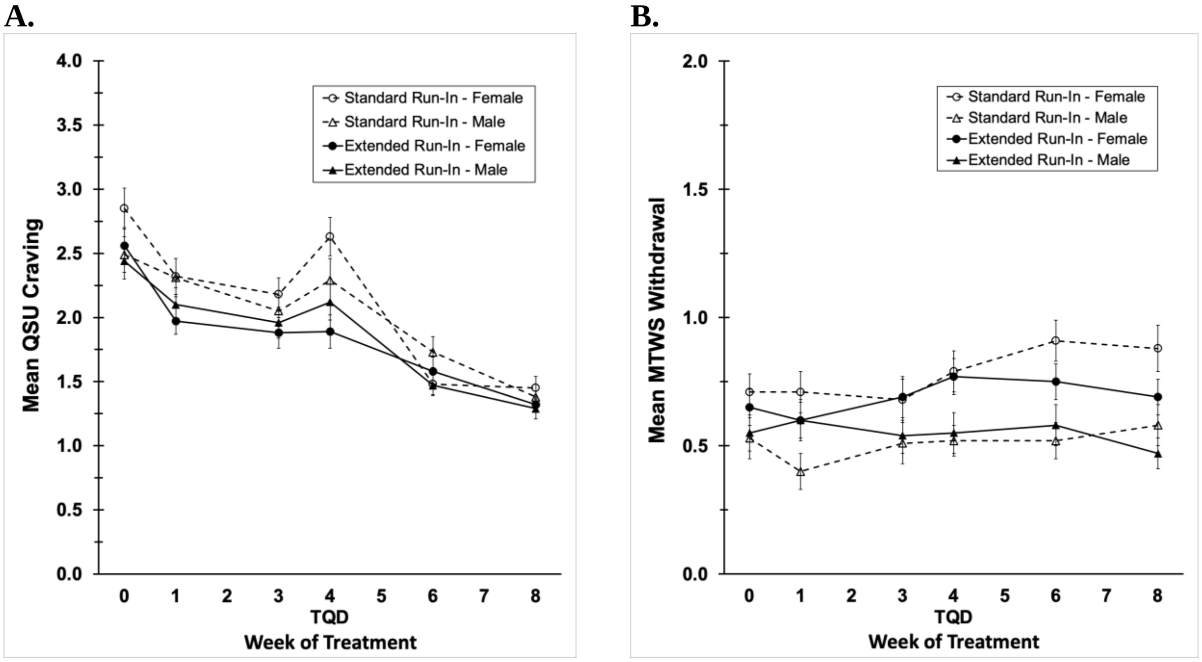

Error bars are +/-1 standard error.

© 2022 Hawk LW Jr et al. *JAMA Network Open*.

eAppendix 5. Pill Count Medication Adherence

Pill count adherence. Pill counts were conducted by a staff member at each clinic visit and EOT (End of Treatment); when a participant failed to return their medication container (which was common), participant self-report was used to assess the number of remaining pills. As for varenicline concentrations, the initial instance of missing pill count data (i.e., because of a missed visit) was treated as missing, but subsequent missing data were assigned a value of 0 (because no medication was dispensed if a participant missed a visit). Percent pill count adherence was computed for each visit and aggregated across the entire 15-week treatment period. Because the resulting distribution was extremely non-normal (bimodal), pill count adherence was dichotomized, with $\geq$80% treated as adherent.

Pill count adherence was analyzed by logistic regression, with gender, group, and their interaction as predictors; none of these effects was statistically significant, *P*s = .71, .62, and .90, ORs = .86, .83, and .94, respectively. The majority of participants in each of the Gender x Group cells were pill-count adherent (79.8% and 76.7% for females and 77.3% and 72.6% for males, in the Standard and Extended Run-In Groups, respectively).

© 2022 Hawk LW Jr et al. *JAMA Network Open*.

eAppendix 6. Symptom Report Data

At intake and each clinic visit, participants completed a 32-item symptom checklist, as in prior work.[21,33,42] Participants then rated endorsed items as mild (does not interfere with daily activities), moderate (interferes with some activities), or severe (no normal activities are possible). Because most severity ratings were mild (e.g., >60% of reports of insomnia and abnormal dreams, >70% of reports of nausea, >80% of flatulence/gas), symptom data were re-coded as absent or present for each of three 3-week study phases: pre-treatment (reported at the Intake Visit and/or treatment Week 0), the pre-quit run-in manipulation phase (reported at Week 1 and/or 3), and the early quit phase (reported at Week 4 and/or 6). Treatment group differences for each potential side effect were evaluated with chi-square tests, separately for each of the three study phases.

Data for all symptoms are presented for all Run-In Group x Study Phase cells in Supplemental Table F1. As expected during the pre-quit run-in manipulation, nausea and abnormal dreams were more commonly reported among the Extended Run-In Group compared to the Standard Run-In Group; these group differences were not statistically significant in the early quit phase. Similar patterns were observed for a range of gastrointestinal symptoms, as well as for other sleep problems and dizziness. During the early quit phase only, agitation, disturbance in attention, and skin swelling were more frequently reported in the Standard Run-In Group and depression tended to be more frequently reported in the Extended Run-In Group.

Suicidality was assessed at each visit with the Columbia Suicide Severity Rating Scale[49] and coded as absent or present for each study phase. No suicidal behavior was reported during the trial, and suicidal ideation was rare: One participant (in the extended run-in group) reported non-specific suicidal thoughts during the pre-quit run-in manipulation and early quit phases of the study.

Symptom occurrence was not generally evaluated as a function of gender (to avoid compounding the number of statistical tests). However, based on our prior work[21], we hypothesized that the run-in group difference in nausea during the pre-quit run-in manipulation phase would be greater among women compared to men. Although the predicted interaction was not statistically significant, Gender x Group p=.36 (see Supplemental Table F2 for details), rates of nausea were in the predicted direction. Post-hoc comparisons were consistent with an increased incidence of nausea among women in the extended run-in group (34%) compared to the standard run-in group (18%), P=.02; for males, rates of nausea were not significantly different between run-in groups (male standard = 17%, male extended = 22%).

Serious adverse events (SAEs). Seven SAEs (pyelonephritis/nephrolithiasis, urinary tract infection, rash/scabies, bilateral hand swelling, bowel obstruction/laparotomy for lysis of adhesions, urinary frequency and incisional pain 3 days after elective inguinal hernia repair, leg pain/DVT) were reported by 6 participants (6/320=1.9%; n=3 in the extended run-in group, n=3 in the standard run-in group). Each SAE was deemed unexpected and unrelated to the study medication by the research team.

© 2022 Hawk LW Jr et al. *JAMA Network Open*.

eTable A5. Percentage of Participants Who Reported Past-Week Presence of Diverse Symptoms, Presented Separately by Treatment Group, During 3 Assessment Phases

**Symptom Cluster** Symptom	**Pre-Treatment Phase (Intake Visit / Clinic 1)**				**Pre-Quit Run-In Manip Phase (Clinic 2 / Clinic 3)**				**Early Quit Phase (Clinic 4 / Clinic 5)**		
	**Run-In Group**		**p-value**		**Run-In Group**		**p-value**		**Run-In Group**		**p-value**
	**Standard** (n = 157)	**Extended** (n = 163)			**Standard** (n = 157)	**Extended** (n = 163)			**Standard** (n = 157)	**Extended** (n = 163)	
**Gastrointestinal**											
Nausea	8.28%	7.98%	0.46		17.69%	29.03%	0.009		28.77%	32.21%	0.26
Vomiting	1.27%	0.61%	0.27		3.40%	7.14%	0.07		6.16%	6.76%	0.42
Abdominal pain	5.73%	4.91%	0.37		5.44%	12.34%	0.02		8.22%	8.78%	0.43
Indigestion	8.28%	10.43%	0.25		4.08%	8.44%	0.06		6.16%	6.76%	0.42
Diarrhea	8.28%	7.36%	0.38		4.76%	8.44%	0.10		3.42%	8.16%	0.04
Constipation	7.01%	9.82%	0.18		15.65%	14.19%	0.36		19.18%	16.89%	0.31
Dry mouth	23.57%	15.34%	0.03		29.25%	21.43%	0.06		25.34%	19.59%	0.12
Flatulence	9.55%	1.84%	0.001		10.20%	7.10%	0.17		6.85%	8.16%	0.34
Gas	14.65%	11.66%	0.21		13.61%	18.06%	0.14		15.75%	17.01%	0.39
**Mood**											
Agitation	19.75%	22.70%	0.26		17.69%	20.65%	0.26		28.08%	19.33%	0.04
Depressed mood	17.20%	12.27%	0.11		10.88%	13.64%	0.23		9.59%	15.44%	0.06
Irritability	31.85%	35.58%	0.24		31.97%	35.03%	0.29		38.36%	32.67%	0.15
Hostility	1.27%	5.52%	0.02		3.40%	6.49%	0.11		4.11%	7.48%	0.11
Anxiety	21.66%	22.09%	0.46		17.01%	16.77%	0.48		21.92%	16.56%	0.12
**Sleep**											
Insomnia	18.47%	15.34%	0.23		18.24%	18.83%	0.45		19.86%	22.30%	0.30
Abnormal dreams	5.73%	3.68%	0.19		10.20%	26.62%	<0.001		16.44%	23.49%	0.06
Other sleep problems	18.47%	14.11%	0.15		9.52%	16.03%	0.04		10.27%	14.86%	0.12
Sleepwalking	0.00%	0.00%	----		0.00%	0.65%	0.16		0.68%	0.68%	0.50

Assessment phases include pre-treatment (assessments occurring before the first distribution of study medication; intake visit and Clinic 1), pre-quit run-in manipulation (when the extended run-in group was taking varenicline and the standard run-in group was taking placebo; Clinic 2 and 3), and the early quit phase (Clinic 4 and 5, which correspond to the target quit date [TQD] and 2 weeks post-TQD).

© 2022 Hawk LW Jr et al. *JAMA Network Open*.

eTable A5 (continued)**.**

**Symptom Cluster**<br>Symptom	**Pre-Treatment Phase (Intake Visit / Clinic 1)**				**Pre-Quit Run-In Manip Phase (Clinic 2 / Clinic 3)**				**Early Quit Phase (Clinic 4 / Clinic 5)**		
	**Run-In Group**		**p-value**		**Run-In Group**		**p-value**		**Run-In Group**		**p-value**
	**Standard** (n = 157)	**Extended** (n = 163)			**Standard** (n = 157)	**Extended** (n = 163)			**Standard** (n = 157)	**Extended** (n = 163)	
**Cardiovascular**											
Increased heart rate	1.91%	6.75%	0.02		4.76%	5.19%	0.43		2.74%	3.38%	0.38
Palpitations	1.27%	3.68%	0.08		2.04%	4.55%	0.11		2.05%	0.68%	0.16
Chest pain	3.82%	5.52%	0.24		3.40%	5.81%	0.16		2.74%	4.73%	0.18
Irregular heartbeat	1.91%	3.68%	0.17		2.04%	3.25%	0.26		0.00%	0.68%	0.16
**Neurological**											
Headache	29.94%	23.93%	0.11		26.53%	20.13%	0.09		19.86%	17.45%	0.30
Dizziness	6.37%	7.98%	0.29		6.12%	14.29%	0.009		6.16%	8.05%	0.26
Disturbed attention	6.37%	2.45%	0.04		4.08%	6.49%	0.17		6.85%	2.72%	0.05
**Neurological**											
Headache	29.94%	23.93%	0.11		26.53%	20.13%	0.09		19.86%	17.45%	0.30
Dizziness	6.37%	7.98%	0.29		6.12%	14.29%	0.009		6.16%	8.05%	0.26
Disturbance in attention	6.37%	2.45%	0.04		4.08%	6.49%	0.17		6.85%	2.72%	0.05
**General**											
Fatigue	27.39%	23.93%	0.24		22.30%	18.06%	0.18		21.92%	18.79%	0.25
Weakness	5.73%	8.59%	0.16		4.76%	5.19%	0.43		6.85%	5.44%	0.31
Other	2.55%	2.45%	0.48		2.04%	1.95%	0.48		3.42%	2.04%	0.23

© 2022 Hawk LW Jr et al. *JAMA Network Open*.

eTable A6. *P* Values, Odds Ratios (ORs), and 95% CIs for Nausea, Presented for the Pretreatment Phase (Weeks −1 and 0), Prequit Run-In Manipulation Phase (Weeks 1 and 3), and Early Quit Phase (Weeks 4 and 6)

Nausea	**Pre-Treatment Phase**		**Pre-Quit Run-In Manipulation Phase**		**Early Quit Phase**	
	***p* value**	**OR (95%CI)**	***p* value**	**OR (95%CI)**	***p* value**	**OR (95%CI)**
Treatment Group	.93	0.97 (0.43,2.16)	.02	1.91 (1.10,3.31)	.55	1.16 (0.70,1.92)
Gender	.32	0.65 (0.28,1.51)	.19	0.60 (0.40,1.20)	.01	0.51 (0.30,0.86)
Group x Gender	.62	0.65 (0.12,3.51)	.36	0.59 (0.19,1.82)	.32	1.70 (0.60,4.86)
Treatment Group for Males only	.65	0.73 (0.19,2.84)	.46	1.39 (0.58,3.31)	.25	1.63 (0.71,3.77)
Treatment Group for Females only	.82	1.13 (0.41,3.06)	.02	2.35 (1.15,4.78)	.89	0.96 (0.51,1.81)

© 2022 Hawk LW Jr et al. *JAMA Network Open*.
