# Supplementary material for: Effect of Extending the Duration of Prequit Treatment With Varenicline on Smoking Abstinence: A Randomized Clinical Trial
Source: JAMA Netw Open. 2022 Nov 11;5(11):e2241731. doi: 10.1001/jamanetworkopen.2022.41731 (PMC9652761; doi:10.1001/jamanetworkopen.2022.41731)
Supplement: Supplement 3. — Data Sharing Statement [file jamanetwopen-e2241731-s003.pdf]

Data Sharing Statement

Hawk, Jr. Effect of Extending the Duration of Prequit Treatment With Varenicline on Smoking Abstinence. *JAMA Netw Open*. Published November 11, 2022.
doi:10.1001/jamanetworkopen.2022.41731

Data
Data available: Yes
Data types: Deidentified participant data, Data dictionary
How to access data: Data will be shared via the National Addiction and HIV Data Archive Program (https://www.icpsr.umich.edu/web/pages/NAHDAP/index.html).
When available: beginning date: 01-31-2023

Supporting Documents
Document types: Informed consent form
How to access documents: By request to lhawk@buffalo.edu
When available: With publication

Additional Information
Who can access the data: Any, provided they meet the standard requirements of the National Addiction and HIV Data Archive Program
Types of analyses: Any
Mechanisms of data availability: Standard agreement with the National Addiction and HIV Data Archive Program
